# Functionalized Gold Nanoparticles and Halogen Bonding Interactions Involving Fentanyl and Fentanyl Derivatives

**DOI:** 10.3390/nano14110917

**Published:** 2024-05-23

**Authors:** Molly M. Sherard, Jamie S. Kaplan, Jeffrey H. Simpson, Kevin W. Kittredge, Michael C. Leopold

**Affiliations:** 1 Department of Chemistry, Gottwald Center for the Sciences, University of Richmond, Richmond, VA 23173, USA; mackey.sherard@richmond.edu (M.M.S.); jamie.kaplan@richmond.edu (J.S.K.); jsimpson@richmond.edu (J.H.S.); 2 Department of Chemistry, Joan P. Brock School of Math and Natural Sciences, Virginia Wesleyan College, Virginia Beach, VA 23455, USA; kkittredge@vwu.edu

**Keywords:** opioid, fentanyl, halogen bonding, gold nanoparticle, monolayer-protected cluster, cyclic voltammetry

## Abstract

Fentanyl (FTN) and synthetic analogs of FTN continue to ravage populations across the globe, including in the United States where opioids are increasingly being used and abused and are causing a staggering and growing number of overdose deaths each year. This growing pandemic is worsened by the ease with which FTN can be derivatized into numerous derivatives. Understanding the chemical properties/behaviors of the FTN class of compounds is critical for developing effective chemical detection schemes using nanoparticles (NPs) to optimize important chemical interactions. Halogen bonding (XB) is an intermolecular interaction between a polarized halogen atom on a molecule and e^−^-rich sites on another molecule, the latter of which is present at two or more sites on most fentanyl-type structures. Density functional theory (DFT) is used to identify these XB acceptor sites on different FTN derivatives. The high toxicity of these compounds necessitated a “fragmentation” strategy where smaller, non-toxic molecules resembling parts of the opioids acted as mimics of XB acceptor sites present on intact FTN and its derivatives. DFT of the fragments’ interactions informed solution measurements of XB using ^19^F NMR titrations as well as electrochemical measurements of XB at self-assembled monolayer (SAM)-modified electrodes featuring XB donor ligands. Gold NPs, known as monolayer-protected clusters (MPCs), were also functionalized with strong XB donor ligands and assembled into films, and their interactions with FTN “fragments” were studied using voltammetry. Ultimately, spectroscopy and TEM analysis were combined to study whole-molecule FTN interactions with the functionalized MPCs in solution. The results suggested that the strongest XB interaction site on FTN, while common to most of the drug’s derivatives, is not strong enough to induce NP-aggregation detection but may be better exploited in sensing schemes involving films.

## 1. Introduction

Widespread use, addiction, and abuse of opiates continue to ravage societies across the globe with hundreds of thousands of deaths attributed to this class of analgesic drug [1,2,3]. According to the National Center for Drug Abuse Statistics, the twenty-year span from 1999 to 2019 showed over a 500% increase in overdose deaths involving opiates, with 7 in 10 overdose deaths attributable to opioids [4]. Over the last two decades in the United States, the Centers for Disease Control and Prevention has shown an exponential increase in opioid overdose deaths: over 20 per 100,000 people and nearly 107,000 deaths in 2021 [3,5]. In the last five years, illicit use of fentanyl (FTN)-based opioids has drastically exacerbated this rapidly advancing and deadly pandemic [6], a trend which severely worsened during the global COVID-19 crisis [7,8]. Additionally, the consequences of FTN-based opioid abuse and addiction have become increasingly disparate for populations of lower socioeconomic status [9].

**Scheme 1:**
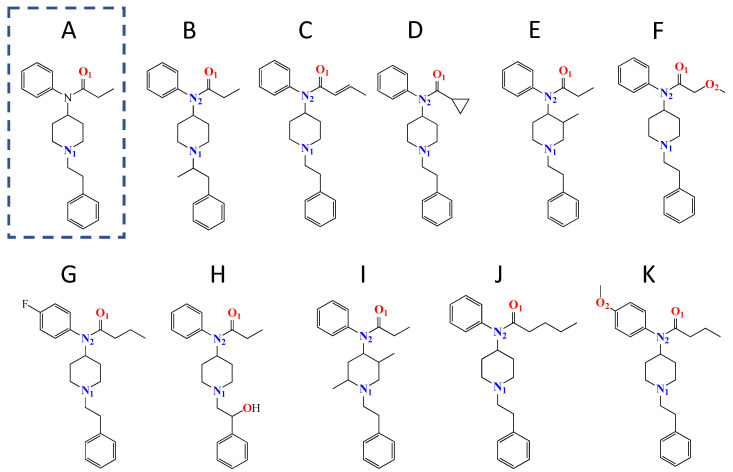
Chemical structures of (**A**) fentanyl (dashed) and select examples of synthetic fentanyl derivatives including (**B**) *a*-mefentanyl, (**C**) crotonylfentanyl, (**D**) cyclopropylfentanyl, (**E**) mefentanyl, (**F**) methoxyacetylfentanyl, (**G**) *p*-fluorobutyrylfentanyl, (**H**) ohmefentanyl, (**I**) phenaridine, (**J**) valerylfentanyl, and (**K**) *p*-methoxybutyrylfentanyl.

FTN-based drugs represent a particularly challenging problem to combat for both society and science due to a number of factors [3,6]. First, as a chemical compound, FTN can be derivatized into a variety of analog structures with equal or greater potency, allowing the drug to have numerous formulations and delivery methods [3]. Scheme 1 highlights the base structure of FTN and shows examples of FTN derivative structures. Second, FTN and many of its derivatives remain highly effective opioids and, in some cases, can be thousands of times more potent than morphine at significantly smaller dosages [10]. The combination of the severe potency of FTN and the ease of structural modifications caused the U.S. Drug Enforcement Agency to classify the entire class of compounds as Schedule 1 [10]. As the illicit use of FTN-related drugs continues to rise, scientific research has sought to develop methodologies to detect the presence of the compounds, a goal made significantly more challenging given the numerous possible structural variations (Scheme 1). Additionally, the latest assessment of illicit FTN drug usage shows that most “street” supplies are now adulterated with drugs designed for animal sedation in veterinary medicine—a development elevating the crisis to a syndemic [11,12] and further complicating the development of effective strategies for fast and accurate detection. FTN-related research is also complicated by the sheer toxicity of the compounds, which makes them dangerous to handle in the lab and expensive to obtain. Given these challenges, any research elucidating the chemical properties, reactivity, or detection of FTN-based compounds represents progress and is of relevance to the field.

As highlighted by recent reviews [13,14,15,16,17], many analytical methods spanning from preliminary screening field tests to more lab-confined, confirmatory methods have been developed for the detection of FTN and/or its analogs. There are recent reports of lab-centered techniques that include the use of lateral flow immunoassays [18], as well as traditional GC-MS characterization [19]. Given the high toxicity of FTN-based drugs [10], however, research has been trending toward preliminary screening tests that can be used by medical first responders, police, and forensic scientists that can deliver fast and accurate assessment of substances at relatively low cost [3,14,18]. As such, there are recent, more specifically focused reviews available summarizing both colorimetric [20] and electrochemical techniques [10,21]—both of which represent methodologies that can be scaled down and/or made portable within a small device [3]. Some of the more notable reports in these areas include work by He and coworkers demonstrating Rose Bengal complexation with FTN for visual detection [22] and reports by Wang et al. demonstrating wearable gloves fixed with microsensor arrays for electrochemical detection of FTN [23]. In 2022, Fulton and coworkers reported a presumptive test for FTN vapor using ion mobility spectrometry with a hand-held monitor [17].

Regardless of the methodology, a common strategy for developing effective chemical sensors, including opioid sensors, has been proposed to incorporate the use of nanomaterials (NMs) within sensing schemes [24,25,26]. Specific examples of this strategy being employed include electrochemical sensors that have electrodes modified with carbon-based NMs such as graphene [27] or carbon nanotubes (CNTs) [28,29,30]. Metallic gold nanoparticles (Au-NPs) have been utilized with guest–host chemistry between FTN and cucurbituril molecules in a fluorescent detection method [31]. One of the issues with the bulk of such studies is that they normally only target either base FTN [22,31] or a specific derivative of FTN (e.g., norfentanyl [29]), frequently also requiring trained personnel, costly instrumentation, and/or pretreatment of samples [19]. As such, the field remains definitively open to investigating new chemical characteristics of this class of molecule with a particular emphasis on molecular properties that may apply to multiple derivatives of the drug and/or be exploited for the development of detection schemes.

Halogen bonding (XB) is a non-covalent intermolecular interaction that develops when a halogen atom on a molecule known as an XB donor develops a localized region of electron deficiency because of nearby electron-withdrawing groups (EWGs). This region of electron deficiency (δ+), known as the “sigma (σ) hole”, enables a largely electrostatic interaction with another molecule that serves as a Lewis base (XB acceptor) with specific regions of high electron density (δ−) [32,33]. Given the positioning of the σ-hole along the internuclear bond axis, XB interactions tend to be more linear than other intermolecular interactions like *hydrogen* bonding. The XB donor system is highly tunable due to the polarizability of different halogen atoms and control over the number, type, and location of nearby EWGs [34]. As such, XB donor systems affixed to substrates with CNTs have been effectively demonstrated as sensors for gas-phase molecules [35,36], including the detection of explosives [37]. Recent work in our laboratory has successfully harnessed XB donor capability to Au-NPs, specifically alkanethiolate-protected gold cores with protruding perfluorinated benzenes featuring an iodine σ-hole [38]. These functionalized Au-NPs were then shown to be able to detect neonicotinoid molecules in solution via XB interactions [39]. Considering the structural motifs present in the FTN and its derivatives (Scheme 1), including the presence of multiple XB acceptor sites for most of the molecules, XB interactions present a significant property for this class of molecule and are worthy of further exploration.

In this work, we explore the strength and utility of XB interactions related to FTN and its derivatives. The overarching goal of this work is to gain a greater understanding of the XB interactions as well as to provide direction and strategies for working with this important class of compounds to eventually develop sensing systems. Density functional theory (DFT), NMR titrations, and electrochemistry are used to evaluate the strength of XB interactions of FTN via a fragmentation strategy. As in prior studies [34,38,39], the XB interactions of FTN are then explored in the context of XB donor-functionalized Au-NPs using transmission electron microscopy (TEM) and UV–Vis spectroscopy. During the execution of our investigation, we became aware of a parallel study by another group exploring similar systems [40]. That study is a purely computational approach, while our work combines *both* theoretical and experimental techniques to explore XB within FTN systems. Additionally, the parallel study focuses on di-halogen XB donor molecules (e.g., 1,4-diiodotetrafluorobenzene), whereas our work builds on our prior findings in which stronger XB donor entities were identified. In any case, it appears that the exploration of XB interactions with regard to FTN and its analogs is of significant interest to the scientific community and is timely for societal needs.

## 2. Experimental Details

### 2.1. Materials and Instrumentation

Chemicals for the study were purchased at the highest available purity from reputable vendors including Millipore-Sigma (Burlington, MA, USA), AmBeed (Arlinton Hts, IL, USA), Oakwood Chemical (Estill, SC, USA), AABlocks (San Diego, CA, USA) and City Chemical (West Haven, CT, USA), including small amounts of FTN from Cerilliant (Round Rock, TX, USA). All chemicals, except where noted, were used without further purification and/or modification. All aqueous solutions were prepared with ultra-purified water measuring 18.02 MΩ∙cm. A high-performance supercomputer (SPY-DUR) was employed for DFT calculations, while experimental procedures were performed using a Bruker Avance III 400 MHz NMR spectrometer, an Agilent 8453 ultraviolet–visible photodiode array spectrophotometer, and CH Instruments multi-channel potentiostats (Models 1000B or 1030C). TEM images were collected with a JEOL 1010 with an Advanced Microscopy Techniques XR-100 CCD image collection system operating at 80–100 kV on 400 mesh carbon-coated copper grids (Electron Microscopy Sciences (Hatfield, PA, USA).

### 2.2. Computational Methodology

As in prior work from our lab [34,37,38,39], Gaussian16 software [41] was used for gas-phase geometry optimizations and single-point calculations of the XB donors, XB acceptors, and XB adducts using the M06 functional [42] with geometry optimization (cc-pVDZ) [43,44] and single-point basis sets (cc-pVTZ) [43] to estimate relevant parameters of interaction energy (ΔE_int_), XB distance (X**··**B), and XB “bond angle” (R-X**··**B). As in other reports, the focus of the computational analyses is to obtain relative comparisons of energies for an assessment of the thermodynamic stability of XB adducts where more negative ΔE_int_ values indicate more strongly interacting adducts. For molecules containing iodine atoms, Dirac–Fock (MDF; small 28 e^−^) effective pseudopotentials and basis sets were employed [45,46]. Frequency analyses were used to reinforce that the geometry-optimized structures corresponded to true minima on the respective potential energy surfaces, and the optimized geometries of all XB adducts were visualized using the GaussView16 program [47].

### 2.3. NMR Titrations

NMR titrations were conducted as described in prior studies [34,38]. Briefly, a titration involved the measurement of sequential samples in which the concentration of the XB donor was held constant (0.0525 M), while the concentration of the XB acceptor was systematically increased from 0.0 M up to as great as 4.0 M in order to saturate the potential interaction being studied. If XB intermolecular interactions create a complex between the donor and acceptor molecules, then significant chemical shifts of the XB donor *ortho*-fluorine are observed. The magnitude of these chemical shifts, which can be plotted as a function of XB acceptor concentration, results in a binding isotherm treated with non-linear regression analysis to yield a binding or association constant (K_a_ value) that is directly proportional to XB interaction strength [38].

### 2.4. Electrochemical Measurements

In these studies, evaporated gold film electrodes (Evaporated Metal Films—Omega Optical Holdings) serve as the working electrode within an inverted sandwich cell that uses an o-ring to define a 0.32 cm^2^ surface area and employs Ag/AgCl (satrd. KCl) reference and platinum wire counter electrodes. Prior to any modification, the gold surface was electrochemically refreshed in a solution of 0.1 M H_2_SO_4_ with 0.01 M KCl by applying cyclic voltammetry (CV) in the consecutive potential windows of 0.2 to 0.9 V (3 cycles), 0.2 to 1.2 V (3 cycles), and 0.2 to 1.35 V (5 cycles) until the voltammogram shape reflected that of a newly exposed gold surface [48,49]. Clean gold substrates were then modified with thiols (ethanol), alkanethiolate-protected Au-NPs (THF or toluene), and/or exposed to solutions of XB acceptor fragments (cyclohexane) while performing specific electrochemical measurements (specific procedures detailed below). As previously described in other work [38], redox probe voltammetry with potassium ferricyanide (K_4_Fe(CN)_6_) and double-layer capacitance (C_dl_) voltammetry were performed at the different stages of electrode modification as well as before and after exposure to solutions containing XB acceptor molecules. As a general protocol used throughout the study, three reproducible voltammetry cycles were necessary before advancing the result. Additionally, electrochemical cells were gently rinsed with the solvent used in the experiment and subsequently gently treated with the next solvent prior to being exposed to the experimental solution containing the molecule of interest. Current measurements for quantifying C_dl_ for individual voltammetry experiments were recorded at 0.250 V (vs. Ag/AgCl, satrd. KCl), a potential window exhibiting no significant Faradaic current in 4.4 mM potassium phosphate-buffered solution (pH = 7.0) [50].

### 2.5. Synthesis, Functionalization, and Characterization of XB Donor SAMs, Au-NPs, and Au-NP Films

Electrodes modified with two-component (two different thiols) mixed self-assembled monolayers (SAMs) were prepared as in prior studies. Briefly, electrodes were exposed to hexane thiol (C6) solutions (EtOH) for 12 h (overnight) for a base SAM formation. These SAM-modified electrodes were subsequently exposed to solutions of XB donor thiols that were synthesized in-house for the study. A saturated, perfluoro-thiolated ligand terminating with iodine (hexadecafluoro-8-iodooctane-1-thiol) was synthesized from the hexadecafluoro-1,8-diiodooctane (Millipore-Sigma, Burlington, MA, USA) precursor with the assistance of the Kittredge research group at Virginia Wesleyan University. Details of the synthetic procedure, purification, and characterization of the final product (Ligand 1) are provided in the Supplementary Materials, including electrochemical evidence of its thiol behavior at the gold electrodes (Figures SI-1 and SI-2) [51]. The other XB donor thiol, 2,3,5,6-tetrafluoro-4-iodo-N-(4-mercaptophenyl)-benzamide (Ligand 2), had been previously synthesized in a prior study and is utilized here again [38]. These ligands were typically allowed to place-exchange in the C6 SAM for 3–4 h unless otherwise noted with the following concentrations: Ligand 1 (0.5 mg/mL in EtOH); Ligand 2 (1.6 mg/mL in MeOH); DT (5 mM in EtOH); MUA (5 mM in EtOH); NDT or UDDT (5 mM in EOH). The resulting mixed SAMs were verified via changes in C_dl_ and/or linear sweep voltammetry (LSV) in basic solution (0.1 M KOH), the latter experiment showing multiple desorption peaks for the mixed SAMs vs. single-component SAMs (results not included) [52]. Analogous procedures and characterizations were used for the formation of mixed SAMs of C6 with control molecules of either 11-mercaptoundecanoic acid (MUA, Millipore-Sigma, Burlington, MA, USA) or decanethiol (DT, Millipore-Sigma, Burlington, MA, USA).

The synthesis, characterization, and usage of Au-NPs, known as monolayer-protected clusters (MPCs) [53], and MPCs functionalized via place exchange with the XB donor ligand (2,3,5,6-tetrafluoro-4-iodo-N-(4-mercaptophenyl)-benzamide, featuring the (–C_6_F_4_I moiety) and noted as *f*-MPCs (i.e., *functionalized* MPCs), has been described in detail in prior work [38]. Analogous, as-prepared MPCs lacking the XB donor ligand were designated as *unfunctionalized* MPCs (*unf*-MPCs). Both materials were consistent with many past reports on the same or similar materials in terms of their characterization with UV–Vis spectroscopy, NMR spectroscopy, and TEM imaging [38]. It is worth cautioning that the cytotoxicity of metallic nanoparticles and their ability to be internalized by cells is not currently well understood. As such, proper PPE should be employed when handling these materials [54]. The *unf*-MPCs showed a typical *average* diameter of 3.35 (±0.05) nm and an estimated NP composition of Au_1289_(C6)_221_, while post-functionalization via exchange reactions with XB donor ligands resulted in *f*-MPCs with an *average* diameter of 3.43 (±0.15) nm and an NMR-estimated ~50% ligand exchange rate that yielded an *average* composition of Au_1289_(C6)_111_(XB donor ligand)_110_ [55,56]. Examples of UV–Vis spectra and TEM imaging (with histogram analysis performed with Image J—64-bit Java 8) are provided in the Supplementary Materials (Figure SI-3).

Film assemblies of both *f*-MPCs and *unf*-MPCs were created using a previously reported procedure modified for the current study [38]. That is, for MPC film assemblies at electrodes, C6-SAM-modified electrodes were first subjected to place-exchange with 5 mM dithiol-linking ligands (EtOH), either nonane dithiol (NDT, Ambeed, Arlinton Hts, IL, USA) or undecanedithiol (UDDT, Asemblon, Seattle, WA, USA), for 20–30 or 10 min, respectively, followed by 1 h immersion in a 1 mg/mL solution of *f*-MPCs (THF) or *unf*-MPCs (toluene). As in prior studies with MPC film assemblies [38,57], this cycle was repeated 2–3 times until definitive increases in the film’s C_dl_ were observed in conjunction with more irreversible FeCN voltammetry—both indicators of an MPC-film-modified interface.

### 2.6. UV–Vis Spectroscopy and TEM Imaging

UV–Vis spectra of *f*-MPC (THF) or *unf*-MPC solutions (toluene) with concentrations yielding Abs_@500nm_ = 0.2 a.u. were achieved with screw-capped, quartz cuvettes (0.75 mL capacity, 10 × 2 mm pathlength, Type 46, FireflySci (Northport, NY, USA). UV–Vis spectra of these materials were obtained before and after the addition of the XB acceptor, including FTN. Cuvettes were routinely cleansed with aqua regia in between samples. It is worth noting that aqua regia, a 3:1 ratio of concentrated HCl/HNO_3_, is extremely dangerous, requires appropriate PPE, and should never be placed in a sealed container. Aliquots (5 μL) of the XB acceptor and MPC mixtures were extracted from the cuvettes for drop-casting on 400 mesh, carbon-coated copper TEM grids (Electron Microscopy Sciences) that were then inverted to dry before imaging. Composite characterization of the MPC materials at the final stage of TEM imaging analysis relied on images from ≥5 different grid locations per sample.

## 3. Results and Discussion

### 3.1. DFT Calculations for Fentanyl and Fentanyl Derivatives

**Figure 1:**
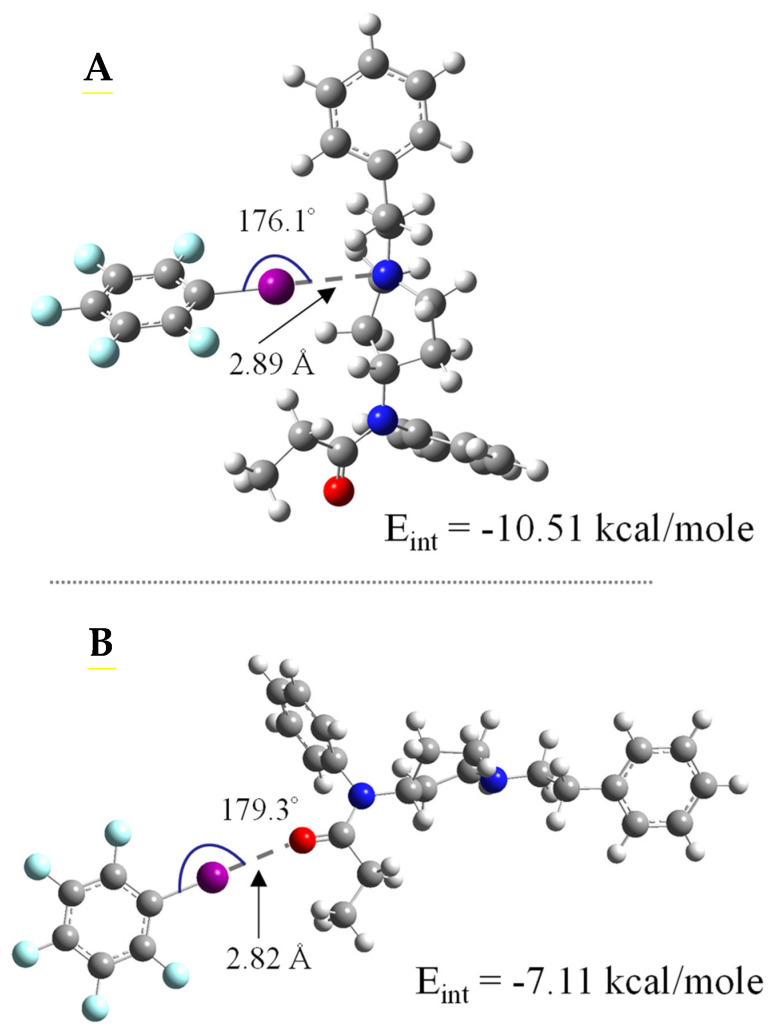
DFT-generated, geometry-optimized structure adducts of IPFB interacting with FTN at the (**A**) N1 and (**B**) O1 XB-accepting sites. Notes: Geometry-optimized XB adducts of IPFB with other FTN derivatives are provided in Supplementary Materials with atomic color codes showing carbon (grey), hydrogen (white), nitrogen (blue), oxygen (red), fluorine (light blue) and iodine (fuchsia).

An investigation of an entire class of molecules can benefit from DFT calculations, as they elucidate structural motifs related to specific intermolecular interactions. In our study, DFT was used with FTN and its structural analogs (Scheme 1) to suggest the number, location, and strength of XB acceptor sites on the molecules. A preliminary scan of this set of molecules suggests that most of the compounds have two to three potential sites of high electron density that may interact with the σ-hole (δ+) of an XB donor molecule: the tertiary nitrogen (N1), the amide oxygen (O1), and the amide nitrogen (N2)—see Scheme 1. In some cases, such as with methoxyacetylfentanyl (Scheme 1, H), there can be a second oxygen site as well (O2). For the majority of the structures, however, there are only two viable XB acceptor sites, given that the electronegative oxygen in the amide draws electron density away from the amide nitrogen. As such, the amide nitrogen (N2) is not considered a significant XB acceptor site [39,58]. To make measurements on the N1, O1, and O2 XB-accepting sites, the interaction with iodopentafluorobenzene (IPFB), an established strong XB donor featuring a large σ-hole, was measured. Strong XB interactions will be characterized by DFT as having more linear (~180°) “bond” angles (R-X**··**B or θ_XB_), “bond” distances (X-B) shorter than van der Waals distances, and more negative interaction energies (−ΔE_int_) [58]. Figure 1 shows an example of the results of this type of DFT calculation between IPFB and FTN at the N1- and O1-accepting sites where the E_int_, θ_XB_, and X-B values calculated from DFT were −10.51 kcal/mole, 176.1°, and 2.89 Å versus −7.11 kcal/mole, 179.3°, and 2.82 Å for the N1- and O1-accepting sites, respectively. Analogous figures for the other FTN derivatives interacting with IPFB are provided in the Supplementary Materials (Figures SI-4–SI-12), and a summary of the DFT calculations is provided in Table 1. These results suggest that the N1 site yields the most negative ΔE_int_ values, typically 9–12 kcal/mole and ~1–3 kcal/mole more negative than the O1 site. For molecules that feature an O2-accepting site (e.g., methoxyacetylfentanyl, ohmefentanyl), the parameters are similar to the N1 site. Collectively, DFT results suggest that FTN and the selected derivatives of FTN each feature at least two XB-accepting sites of significance. That said, translating theoretical calculations of this nature to actual measurements of such interactions in real systems has proven to be non-trivial in prior studies, requiring strategic implementation of experimental design and heavy consideration of solvent effects [34,38,39].

**Scheme 2:**
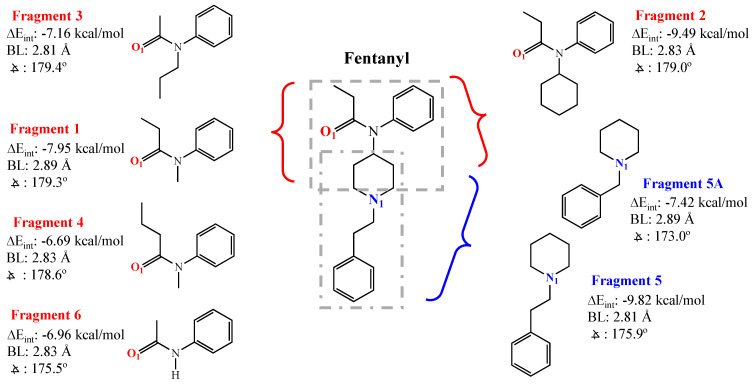
DFT results from analysis of IPFB interacting with specific “fragments” of the FTN structure that emphasize O1- and N1-accepting sites: Fragment 1 (N-methyl-N-phenylpropanamide, not purchased), Fragment 2 (N-cyclohexyl-N-phenylpropanamide, AA Blocks, Inc., San Diego, CA, USA), Fragment 3 (N-propylacetanilide, City Chemical LLC, West Haven, CT, USA), Fragment 4 (N-methyl-N-phenylbutanamide, AA Blocks, Inc., San Diego, CA, USA), Fragment 5 (1-phenyletherpiperidine, not purchased), Fragment 5A (1-benzylpiperidine, Ambeed, Arlinton Hts, IL, USA), and Fragment 6 (Acetanilide, Oakwood Chemical, Estill, SC, USA).

Given the toxicity of FTN and its analogs, DFT was again employed to lend support toward an experimental strategy that alleviates some of the risk of handling large quantities of multiple forms of these dangerous chemicals. To execute this study, the FTN molecule was “fragmented”, as shown in Scheme 2, a strategy in which each XB-accepting site is modeled with a safer, commercially available molecular fragment possessing similar structure and/or chemistry. Analogous fragmentations of select FTN derivatives of interest identified in other studies [40] are included in the Supplementary Materials (Schemes SI-1 and SI-2). DFT calculations were then run for each fragment acting as an XB acceptor with the XB donor IPFB. DFT findings shown within Scheme 2 for FTN indicate that Fragment 2, featuring an O1 site, and Fragment 5, featuring the N1 site, produced the most negative ΔE_int_ values. DFT structure results for all fragments are included in the Supplementary Materials (Tables SI-0 and SI-1, and Figures SI-13–SI-24). Fragment 5, exhibiting the most negative ΔE_int_, is of particular interest given that it is a structural motif common to most FTN derivatives (see Scheme 1, structures A-K). However, while Fragment 5 is commercially available for experimentation, it is prohibitively expensive. As such, Fragment 5A (Scheme 2), herein referred to as Frag 5A, is introduced as a mimic of Fragment 5, as it has similar DFT results (Scheme 2) but is more amenable to experimentation in the lab.

### 3.2. NMR Titrations: Fentanyl Fragments

NMR titration has proven to be an effective experimental tool for assessing the strength of XB interactions for a number of systems [38,39,59,60]. In those studies, for example, the ^19^F NMR resonance of the fluorine adjacent to the halogen (*ortho* position) on the XB donor can be monitored as the system is subjected to increasing concentrations of an XB acceptor molecule. The shift in the ortho-fluorine NMR signal is indicative of XB interaction strength and ultimately translated into a binding isotherm that, when analyzed with non-linear regression, yields an association constant (K_a_) that reflects the strength of XB interaction of the adduct [61]. In prior work by our lab and others [38,60], NMR titrations of this nature, combined with DFT analysis, both established the significantly higher XB donor capability of IPFB (vs. dihaloperflurobenzenes, for example) as well as the strong σ-hole of XB donors having non-fluorine EWGs (e.g., amide) *para*-substituted to the iodine [38]. Herein, we employ the same strategy for the current system of interest.

**Figure 2:**
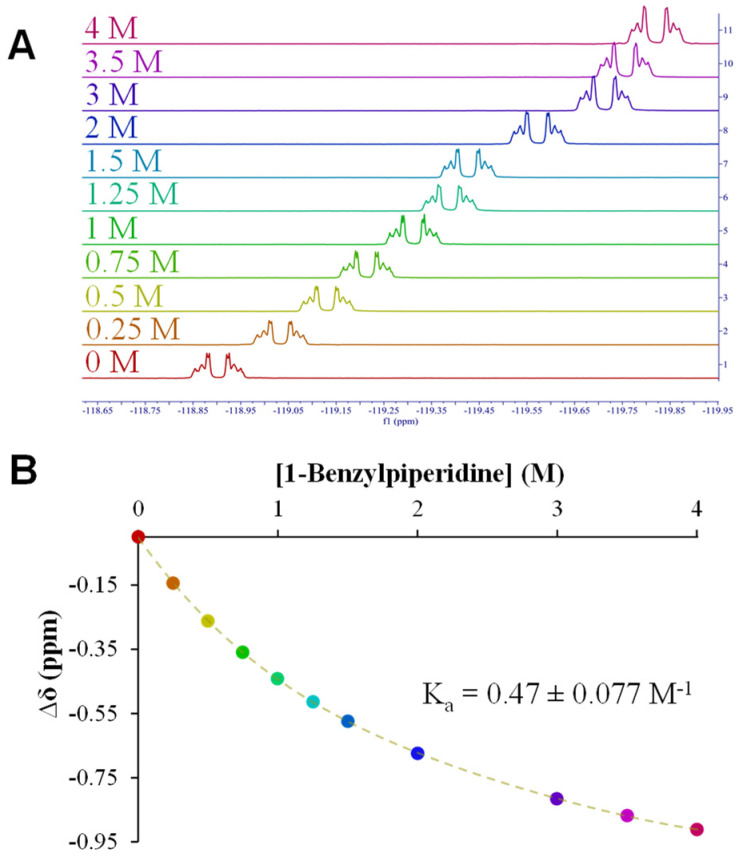
Representative NMR titration for determining XB interaction strength: (**A**) ^19^F NMR spectra of the *o*-fluorine resonance on IPFB (XB donor) when subjected to increasing concentrations of the XB acceptor, 1-benzylpiperidine, or Fragment 5A (Scheme 2); (**B**) the corresponding binding isotherm that yields an association constant (K_a_) indicative of XB interaction strength (solvent: cyclohexane). Note: NMR titration analyses for other fragments are provided in Supplementary Materials.

NMR titrations were used as a measure of the strength of XB interactions between the DFT-identified fragments (Scheme 2) and IPFB as a means of screening the fragments for maintaining the XB acceptor capability in solution. As an example, Figure 2A depicts the ^19^F NMR titration results obtained between IPFB and Frag 5A, showing spectra as the concentration of the fragment is systematically and significantly increased. That information translates to the binding isotherm shown in Figure 2B and the resulting average K_a_ value of 0.47 (±0.08) M^−1^ for the system. NMR titration results for other fragments tested in solution with IPFB are provided in the Supplementary Materials (Figure SI-25–SI-30). Compared to other systems we have tested for XB interactions in solutions, the K_a_ value of 0.47 M^−1^ suggests only a moderate-to-weak interaction. That said, this interaction was found to be the most robust in the titration analysis of the current systems we explored—an experimental result that is consistent with DFT trends (i.e., Fragment 5A showed the most negative ΔE_int_ of all fragments). The impact of having solvent present in the experimental systems that weaken XB interactions is unsurprising and has been observed in prior work as well [34]. Collectively, the titration data suggest that the N1 position on FTN and its derivatives may be the only position capable of promoting XB interactions in a solvent. That said, we also note that the N1 XB acceptor site is common to most forms of the drug (Scheme 1).

### 3.3. Electrochemical Support of XB Interactions Using XB Donor Self-Assembled Monolayers

For this study and others, two types of cyclic voltammetry (CV) experiments have been used in concert to successfully show XB interactions at the interface of an XB donor self-assembled monolayer (SAM)-modified electrode [38]. Double-layer capacitance (C_dl_) measurements coupled with the voltammetry of redox probes, such as potassium ferricyanide (K4Fe(CN)6), are classic examples of electrochemical surface analysis techniques that can yield results supporting the existence of molecules interacting at an interface via electrostatic, covalent, or other intermolecular interactions like XB [38]. C_dl_ measurements take advantage of the sensitivity of the electric double layer or Helmholtz model of the electrode–solution interface under applied potential in a supporting electrolyte. The described experimental system can be modeled as a parallel-plate capacitor where non-Faradaic current flows until there is charge compensation between the electrode and ionic solution. Based on this model, C_dl_ can be qualitatively determined by Equation (1):

(1) $$C_{\mathrm{dl}}\;\approx \;\frac{\varepsilon \cdot \varepsilon_{{}^{\circ}}}{d}$$

where ε_o_ is the permittivity of the free space constant, while ε and d represent the dielectric constant (polarizability) and the distance separating the plane of charge at the electrode surface to the plane of charge in solution [62]. As such, anything that increases that distance and/or decreases the dielectric between the planes of charge, such as an adlayer of interacting molecules, will notably decrease C_dl_, a common phenomenon observed when alkanethiol modification of electrodes to form SAMs is performed [50]. Experimentally, C_dl_ can be quantified by running CV in the absence of a redox species (i.e., only supporting electrolyte) and applying the following equation:

(2) Cdl μFcm2=icathodic+ianodic (amps) 2 · ν(V/sec) · Acm2· 10−6 ,

where the numerator is the absolute value of the total current or the anodic and cathodic current combined (amps), ν is the scan rate, and A is the area of the working electrode.

**Scheme 3:**
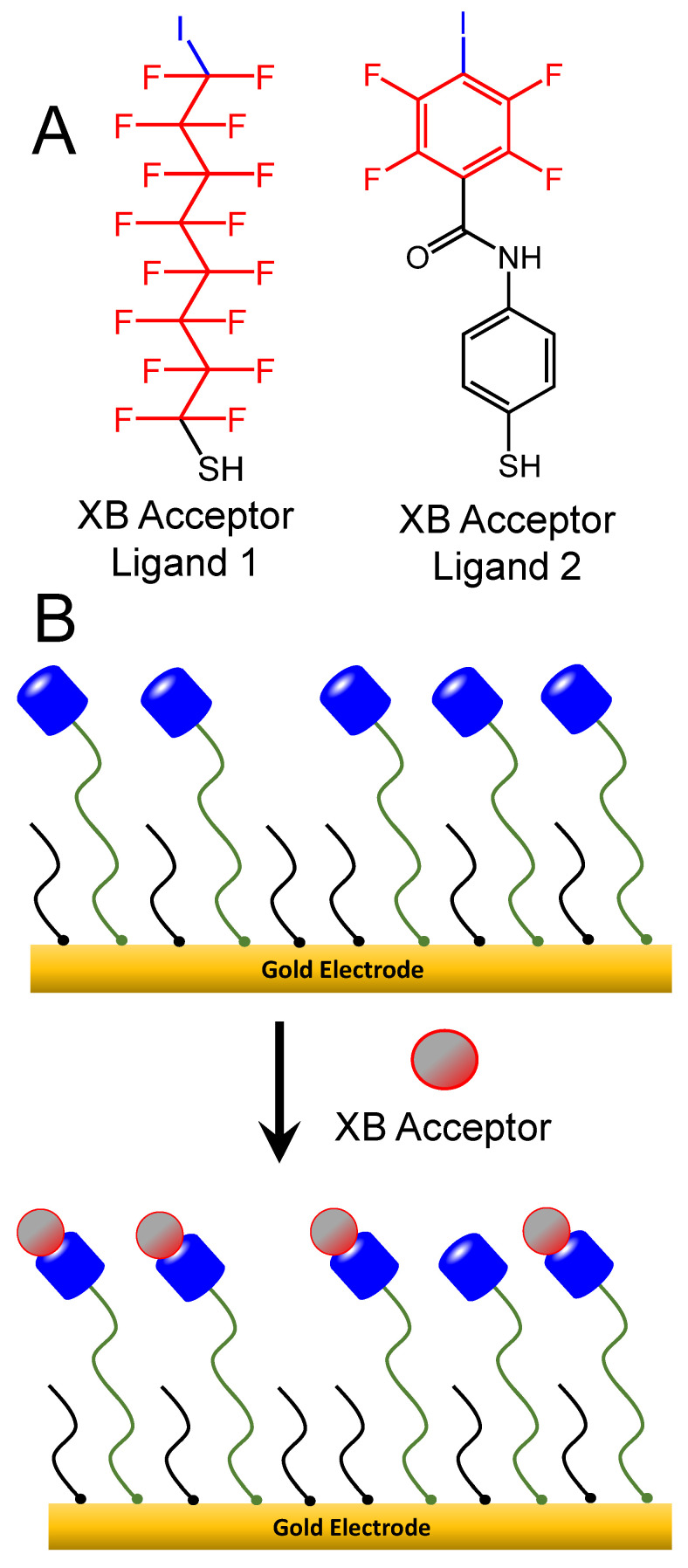
(**A**) XB donor ligands, hexadecafluoro-8-iodooctane-1-thiol (Ligand 1, **left**) and 2,3,5,6-tetrafluoro-4-iodo-N-(4-mercaptophenyl)-benzamide (Ligand 2, **right**), were place-exchanged into hexanethiolate (C6)-modified films to form mixed SAMs; (**B**) mixed SAMs featuring XB donor sites were exposed to XB acceptor molecules and tested electrochemically.

**Figure 3:**
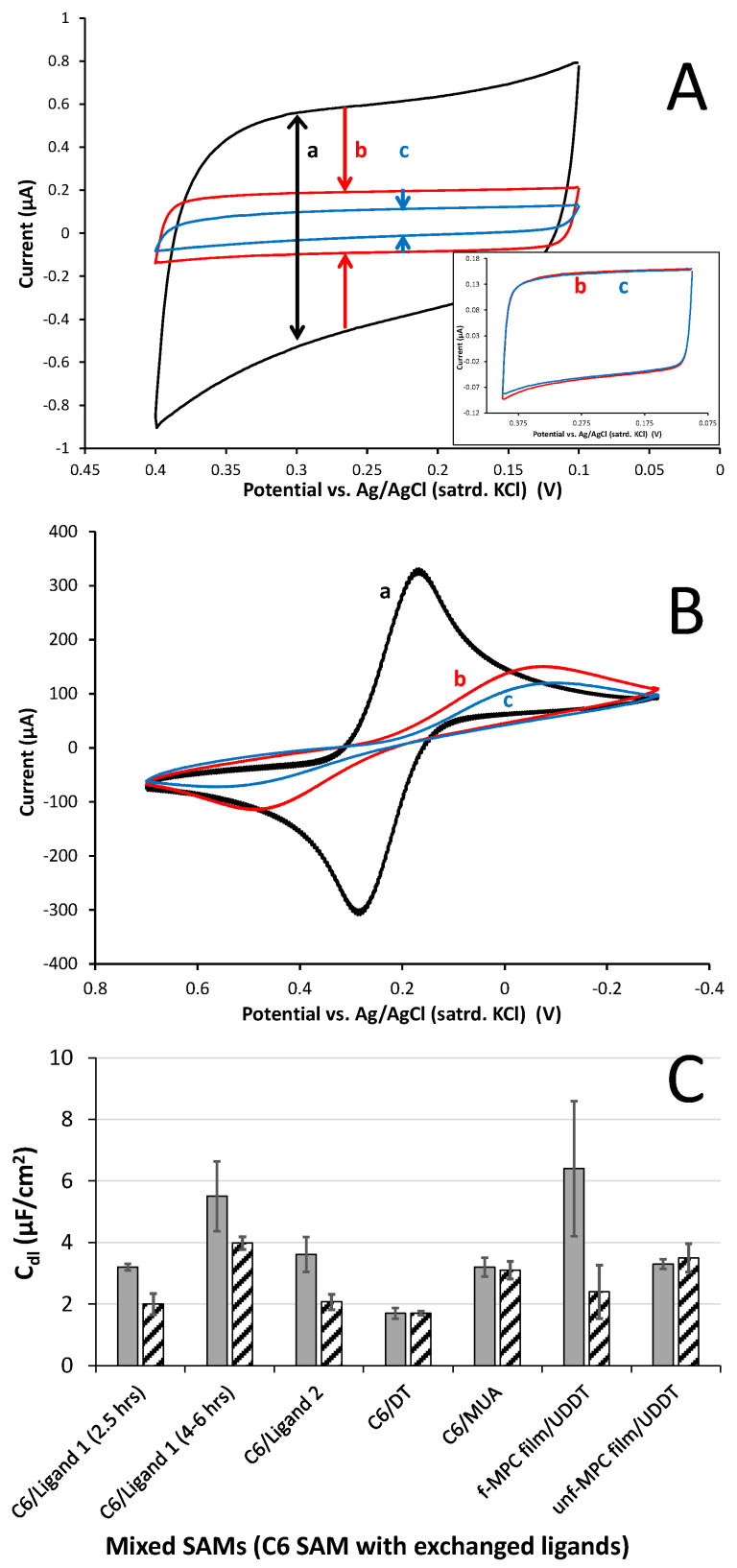
Representative CV of (**A**) C_dl_ (4.4 mM PBS) and (**B**) 5 mM K_3_Fe(CN)_6_ in 0.5 M KCl at (a) bare/clean gold electrode, (b) a C6/Ligand 1 mixed SAM modification, and (c) the same C6/Ligand 1 mixed SAM after exposure to 5 mM Frag 5A (1-BP) solution (cyclohexane); control film of C6/MUA mixed SAM exposed to the same Frag 5A solution (inset); and (**C**) C_dl_ results summary for films before (grey) and after (hashed) overnight (12 h) exposure to Frag 5A. Note: Scan rates = 100 mV/s; error bars represent standard error (n ≥ 3).

Based on prior use of this strategy, it was conceivable that, given the DFT and NMR results for 1-BP or Frag 5A, XB interactions between that fragment, emphasizing the N1-accepting site, could be observed electrochemically at an interface presenting strong enough XB donor ligands. This possibility was pursued by forming SAM-modified gold electrodes, which were subsequently further modified with XB donor ligands via place exchange to form mixed or two-component mixed SAMs. These mixed SAMs presenting XB donor sites could then be electrochemically characterized before and after exposure to a solution of Frag 5A. Mixed SAMs of this nature were selected for the study based on literature reports that show they can interact effectively with interfacial adsorbates [49]. Newly formed hexanethiol (C6) SAM-modified electrodes were place-exchange-functionalized with solutions of either hexadecafluoro-8-iodooctane-1-thiol (Scheme 3, Ligand 1) or 2,3,5,6-tetrafluoro-4-iodo-N-(4-mercaptophenyl)-benzamide (Scheme 3, Ligand 2), both of which have shown significant σ-holes and strong XB donor behavior in prior work [38,60]. As expected, modification of the clean gold substrates with any type of thiol-based SAM or mixed SAM resulted in drastic decreases in C_dl_ and significantly blocked FeCN electrochemistry versus a bare gold electrode (Supplementary Materials: Figures SI-31 and SI-32). It was observed that as hexadecafluoro-8-iodooctane-1-thiol exchanged into the C6 SAM to form the mixed SAM, C_dl_ increased slightly with exchange time, though it remained very low overall compared to bare gold (Supplementary Materials—Figure SI-33). For the current study, changes from the baseline C_dl_ of the mixed SAM versus C_dl_ after exposure to a fragment molecule were of most interest, as they could be directly attributed to XB interactions. After exposing mixed SAMs to an XB acceptor solution and subsequent gentle rinsing, a decreased C_dl_ coupled with increased blocking of the FeCN redox probe would suggest XB interactions persist between the fragment molecule and the mixed SAM interface (Scheme 3)—a result that could be matched with NMR and DFT results. Figure 3A reflects typical capacitance voltammetry throughout this type of exposure process and captures the significantly lower capacitance of the mixed-SAM-modified gold versus the bare gold, as well as the notable decrease in the film’s capacitance after exposure to a solution containing Frag 5A. As control experiments, analogous procedures were applied to mixed SAMs lacking XB donor capability. Mixed SAMs of C6 with either 11-mercapto-undecanoic acid (MUA) or decanethiol (DT) were exposed for the same time to solutions of Frag 5A and resulted in no significant change in capacitance. Figure 3A (inset) shows the voltammetry of a C6/MUA mixed SAM before and after exposure to Frag 5A, while a similar result achieved with C6/DT is provided in the Supplementary Materials (Figure SI-34). These capacitance results can be coupled with FeCN redox probe voltammetry experiments that show systems exhibiting decreases in C_dl_ after fragment exposure are also blocking diffusing FeCN more effectively (Figure 3B). This suggests that there is an interaction/adhesion between the mixed SAM and the fragment, creating an even more substantial barrier at the interface.

Ligand 2 (Scheme 3) represents an important XB donor ligand for this type of study as it was used previously to modify Au-NPs and effectively accent the material with strong XB donor capability [38,39]. As such, the aforementioned electrochemical analyses were repeated with mixed SAMs of C6 and Ligand 2. The results were very similar to those presented for the C6/Ligand 1 mixed SAMs. Upon exposure to a solution of Frag 5A, C_dl_ decreased with a corresponding increasing blocking behavior toward FeCN redox activity (Supplementary Materials, Figure SI-35).

While numerous analyses using C_dl_ and FeCN probing were conducted for various types of mixed SAMs, it was the changes to C_dl_ of the interfaces before and after exposure to the fragment solution that served as the most instructive tool toward indicating the presence of potential XB interactions. C_dl_ measurement results thus far in the study are summarized in Figure 3C (first five film systems). The collective results allow for some notable observations to be made at this stage of the work. First, upon exposure to solutions of Frag 5A, mixed SAMs presenting XB donor components of either Ligand 1 or Ligand 2 showed significant decreases in capacitance of ~28% and ~43%, respectively, versus control C6 SAMs mixed with either DT or MUA components. In those cases, minimal capacitance decreases (<5%) for those mixed SAMs were observed. Secondly, if one examines the first two mixed SAM experiments where Ligand 1 (XB donor ligand) is exchanged into a C6 SAM for different amounts of time (2.5 vs. 4–6 h), a decrease in C_dl_ after Frag 5A exposure persists but does not correlate with ligand exchange time. The films exchanged with Ligand 1 for only 2.5 h exhibited an average decrease in C_dl_ of 38%, while the longer-exchanged film showed a 28% decrease after exposure to Frag 5A. Prior to the exposure, increasing the exchange time of Ligand 1 did correlate with higher initial capacitance as we have observed previously (Supplementary Materials, Figure SI-33). Ligand 2-exchanged mixed SAMs showed the largest average decrease (48%) in C_dl_ after exposure to Frag 5A, suggesting that these particular XB donor ligands are capable of promoting significant XB. Not shown as part of the results in Figure 3C is that the XB effect on films capable of engaging in XB was dependent on the time of exposure to the fragment solution. While the XB interactions between mixed SAMs and Frag 5A could be observed in the voltammetry after only a few hours (2–3) of exposure, the effect was clearly more significant after overnight (12 h) exposure (Supplementary Materials, Figure SI-36). As such, this exposure time was used for all the experiments as the standard for comparing films. The dependence of exposure time as it correlates to the electrochemical evidence suggests that XB interactions require time to organize at interfaces. This particular observation is the primary subject of another ongoing investigation in our lab.

### 3.4. XB Interactions at Interfaces Using XB Donor-Functionalized Gold Nanoparticles

**Figure 4:**
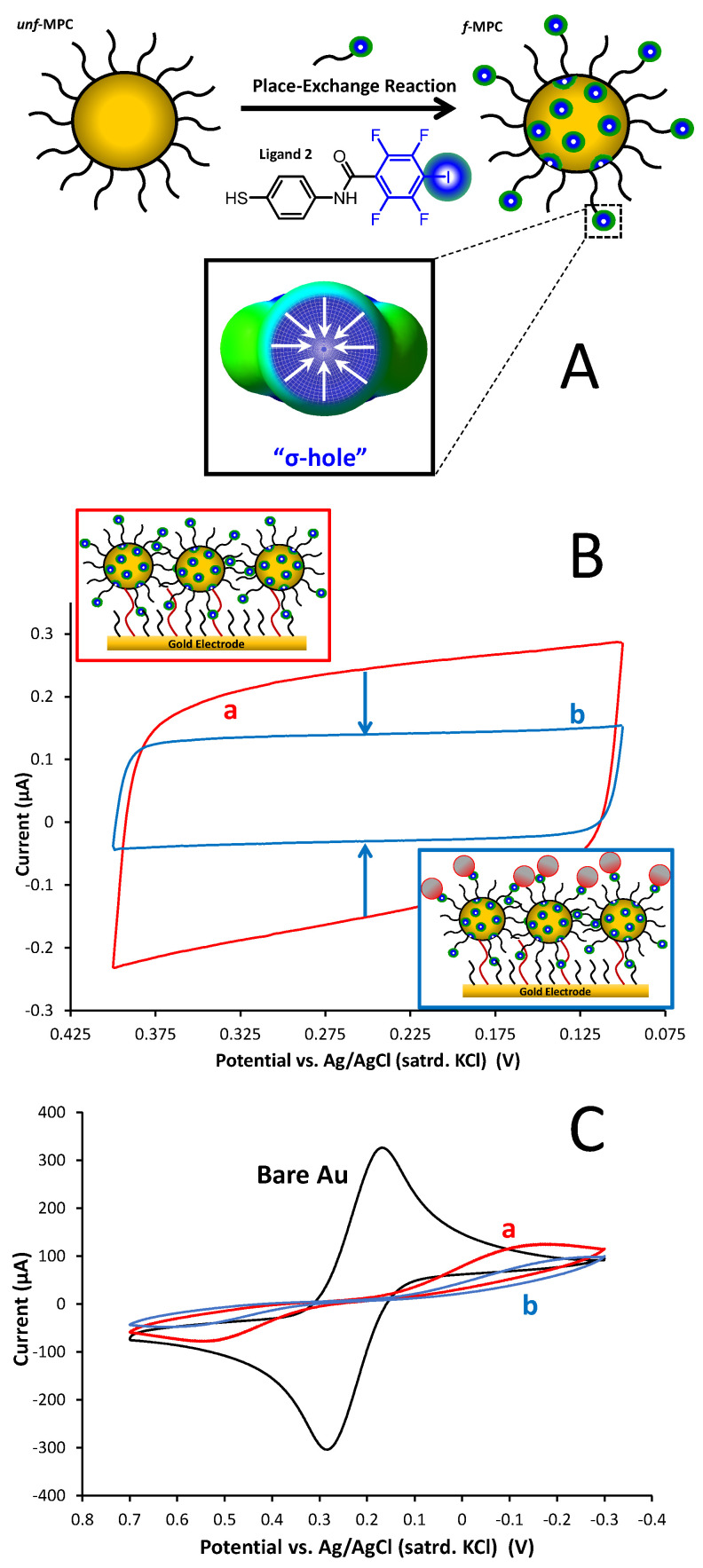
(**A**) Schematic of a place-exchange reaction of *unf*-MPCs with Ligand 2 to create *f*-MPCs with XB donor capability in three dimensions; (**B**) representative CV showing change in C_dl_ of *f*-MPC film assembly before (a) and after (b) the film’s exposure to a 5 mM solution of Frag 5A (1-BP) in cyclohexane; and (**C**) corresponding CV (100 mV/sec) of 5 mM K_3_Fe(CN)_6_ in 0.5 M KCl at the same films and exposure to Frag 5A.

One of the goals of our current study was to assess if XB interactions with FTN could be instigated/enhanced with the use of Au-NPs that have an XB donor capability. The introduction of nanomaterials has been observed to enhance both optical and electronic signals at interfaces [63,64]. As depicted in the scheme of Figure 4A, Ligand 2 was successfully place-exchanged into unfunctionalized, C6-monolayer-protected gold clusters (*unf*-MPCs) to form functionalized MPCs (*f*-MPCs) featuring strong σ-holes at XB donor locations [38]. The *f*-MPC material has been demonstrated to engage in significant XB interactions, acting as XB donors both at solid interfaces (*f*-MPC film assemblies at electrodes) as well as in solution (MPC aggregation) with strong XB acceptor molecules of interest [38]. As part of the current study, similar film assemblies of *f*-MPCs were again formed on solid electrodes and the same electrochemical strategies (Section 3.3) to test if Frag 5A effects were enhanced at the NP interface. Dithiol-linked films of either *f*-MPCs capable of engaging in XB or *unf*-MPCs without that capability were formed at C6-SAM-modified gold electrodes (Figure 4B—top inset). As in prior work with MPC film assemblies, modification of the electrode with any type of MPC resulted in small but consistent increases in film C_dl_ after each exposure to an MPC solution. The increase in C_dl_ is attributed to the surface attachment of MPCs that act as small capacitors with additive dielectric layers as they bind to the interface via dithiol linkages. Additionally, as MPCs adhere to the interface, the electrode becomes more blocked toward solution redox species (e.g., FeCN) as well [38,57,65]. Examples of these results tracking the MPC film assembly are provided in the Supplementary Materials (Figures SI-37). As in Section 3.3, the key result of this technique is the change in C_dl_ and blocking behavior before and after the film is exposed to Frag 5A (XB acceptor), where decreased C_dl_ and more substantial blocking indicates XB interactions between the *f*-MPC film and Frag 5A (Figure 4B–bottom inset). Figure 4 represents typical results from this set of experiments and indicates not only the presence of XB at the *f*-MPC (i.e., decreased C_dl_ and increased FeCN blocking) but also suggests that the effect is enhanced at NP film assemblies versus the mixed SAM interfaces (see Figure 3C), with an average decrease of 68% in capacitance recorded at the *f*-MPC film assemblies after exposure to Frag 5A. Notably, both effects were not significant with films composed of *unf*-MPCs after the same exposure to a Frag 5A solution (Supplementary Materials, Figure SI-38), suggesting that the electrochemical observations at *f*-MPC film assemblies are attributable to XB interactions at the film/solution interface.

The electrochemical analysis of these film systems resulted in a few other important findings. First, given the electrostatic nature of XB as a fundamental interaction, it was hypothesized that either XB donor interface (i.e., mixed-SAMs or *f*-MPC film assemblies) that exhibited XB interactions with Frag 5A would likely have that interaction diminished if exhaustively washed with a highly polar solvent rinse (e.g., combination of MeOH, EtOH, and water). In every case, with robust rinsing of such a polar mixture, the films reverted to capacitance levels near that of the mixed SAM or MPC film assembly prior to exposure to the XB acceptor solution. Correspondingly, the same films also showed less blocking behavior of FeCN after the polar wash. Examples of these results are provided in the Supplementary Materials (Figures SI-39–SI-40). A second, particularly important finding was that none of the Fragments featuring O1 or O2 acceptor sites (Scheme 2) exhibited detectable XB interactions. An example analysis for Fragment 2 (Scheme 2) has been provided in the Supplementary Materials (Figure SI-41).

### 3.5. XB Interactions in Solution Using XB Donor-Functionalized Gold Nanoparticles

One of the primary motivations to harness NPs with the functionality of a particular interaction like XB is that aggregation models for molecule detection may be possible [38]. That is, if a targeted molecule with two or more interaction sites is added to a solution of functionalized NPs and their interaction is strong enough to bridge the NPs, aggregation of the NPs can be detected via a variety of methods including visual changes. Such a system could be the basis of a fast detection scheme that would be particularly useful for on-site testing of chemicals such as FTN. Au-NP aggregation detection schemes of this nature, where an interparticle interaction is exploited, have been extensively studied in a number of cases over the years [66], including a system utilizing XB interactions between *f*-MPCs to detect specific pesticides [39]. In these types of schemes, upon addition of a specific molecule, aggregation can be visible or tracked using spectroscopy, which shows an accompanying decrease in absorbance and/or red shift of the NP’s characteristic surface plasmon resonance band, at times also yielding a colorimetric change as well.

**Figure 5:**
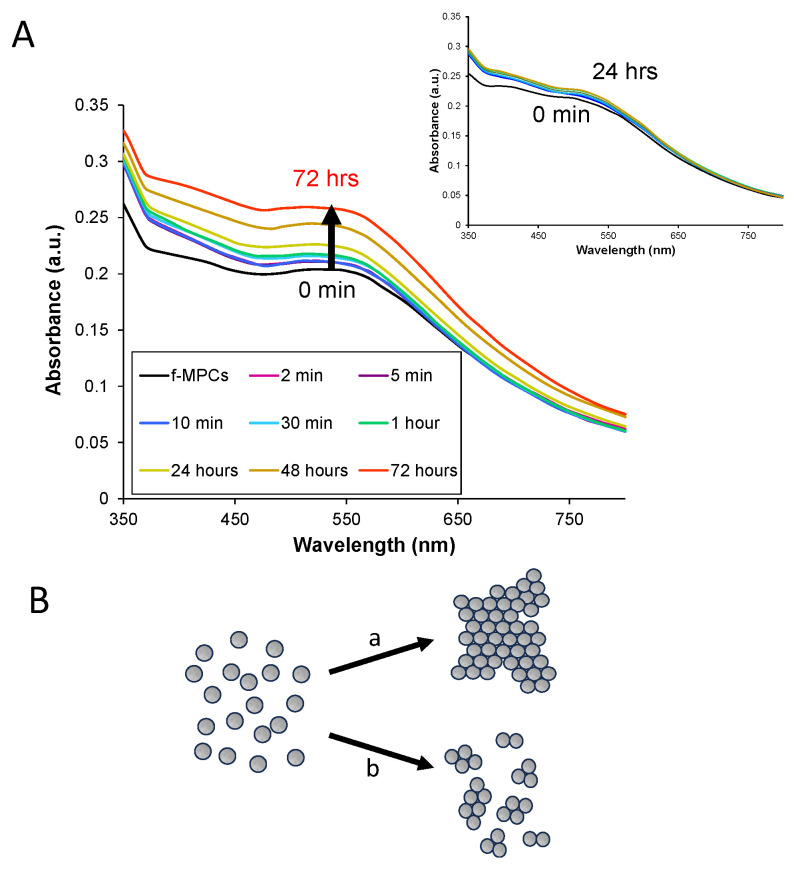
(**A**) UV–Vis spectra of *f*-MPC and *unf*-MPC (inset) solutions monitored over time after the addition of 5 mg of fentanyl; and (**B**) illustrations of NPs undergoing (a) aggregation vs. (b) agglomeration. Note: Both *f*-MPC and *unf*-MPC solutions were prepared to have a starting absorbance at 350 nm of 0.250 a.u. in THF; in the case of the *unf*-MPC with fentanyl, only spectra from the first 24 h are shown for clarity.

**Figure 6:**
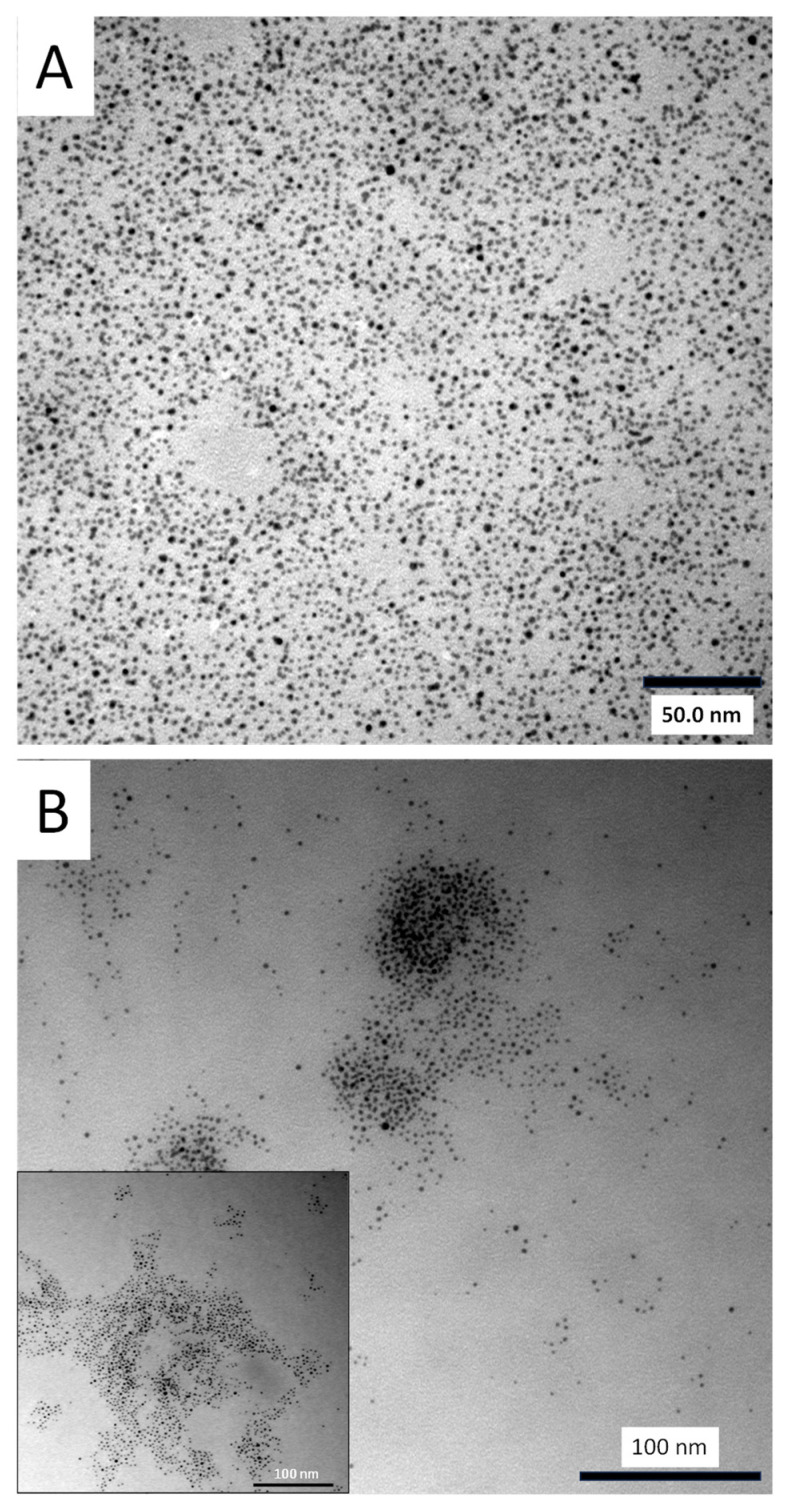
TEM imaging of *f*-MPC solutions both (**A**) before and (**B**) after the addition of fentanyl (5 mg) to the solutions that are consistent with agglomeration of the material in the presence of the opioid (Inset: additional example image of NP agglomeration). Note: dynamic light scattering measurements were inconsistent on a sample-to-sample basis due to the inherent polydispersity of the as-prepared MPCs, the suspected presence of dust/larger particles skewing results, and the weakness of the XB interactions present.

Despite DFT analysis showing multiple XB acceptor sites on the FTN molecule and its derivatives (Table 1), experimental findings from NMR titration and electrochemistry suggest that only the N1 site exhibits significant XB interactions (Fragment 5 or 5A, Scheme 2). As such, FTN’s ability to interact as an interparticle bridge toward aggregation comes into question. However, it was acknowledged that the fragmentation approach may not exactly mimic the actual FTN molecule itself. Given the immediate utility and significance of an NP-based sensing system for FTN, aggregation of the *f*-MPCs in the presence of FTN was explored. Solutions of both *f*-MPCs and *unf*-MPCs were prepared with similar concentrations (Abs@ 350 nm = 0.250 a.u.) and UV–Vis spectra of each were obtained. FTN (5 mg) was added to each solution and subsequently monitored with spectroscopy over time. As seen in Figure 5A, the addition of FTN to solutions of *f*-MPC does not result in aggregation. Instead of decreasing the absorbance and/or red-shift of the plasmon band that typifies aggregation [38], an *increase* in the absorbance and plasmon band is observed. This phenomenon was observed in the spectra as early as 15–30 min after the addition of FTN, becoming more prominent over the course of hours. As shown in Figure 5A (inset), solutions of *unf*-MPCs do not exhibit these effects over the same timeframe after the addition of FTN. The observed increase in the signal suggests that the *f*-MPCs, upon interaction with FTN, display optical behavior more consistent with the agglomeration of NPs rather than aggregation [66]. That is, the addition of FTN causes the *f*-MPCs to gather together and essentially mimic the spectra of larger-diameter NPs. The results suggest that, over time, the *f*-MPCs agglomerate via weak XB interactions with the introduction of FTN but that the interparticle interactions are not strong enough to cause aggregation. TEM analysis of the solutions before and after the addition of FTN is consistent with this hypothesis and spectroscopic data (Figure 5) showing the agglomeration of *f*-MPCs over time (Figure 6). The figures shown are representative and were consistent across the TEM grid when measured in multiple locations. Notably, TEM images of the *unf*-MPC sample did not change significantly over time. Taken collectively, these results reiterate that there are not multiple XB acceptor sites of significant strength on the molecule, but that the strongest site is likely N1.

## 4. Conclusions

Theoretical and experimental analysis of XB interactions with FTN and its derivatives all indicate that the molecule exhibits only moderate XB-accepting capability at the N1 site with the other sites having little to no XB acceptor properties within a solvent system, even when matched with strong XB donors. This finding indicates that NP aggregation schemes designed around XB interactions are likely not possible with FTN and its derivatives. However, the demonstrated ability to harness XB interactions at N1, common to many of the FTN analogs (Scheme 1), at electrode interfaces does suggest that the XB interactions of these particular systems may be better suited to heterogenous sensors at interfaces/electrodes that employ host–guest chemistry [27,31,67,68]. This study also re-emphasizes that XB interactions must be optimized in terms of *both* structure and solvent [34]. This study showed that NMR titrations and surface electrochemistry can be used to observe weaker XB if sufficient time is allowed for those interactions to develop. This observation is strikingly different than prior work using strong XB interactions to aggregate NPs within minutes [38]. The weak XB interactions encountered in this study, coupled with a limited number of experimental systems where strong XB interactions caused a faster, significant change [38,39], suggests that those systems have situational selectivity, requiring solubility of the XB donor and acceptor in a solvent that also promotes the XB interaction. Ultimately, the presented study provides a methodology to experimentally evaluate if theoretically predicted XB interactions are sufficiently strong enough to base a sensor design on, which is useful when dealing with targeted molecules of significant toxicity and/or cost.

## Figures and Tables

**Table 1:** M06-2X/cc-pVTZ/M06-2X/cc-pVDZ interaction energies (∆E_int_), bond distances, and bond angles of XB adducts of XB donor IPFB with fentanyl and various fentanyl derivatives (XB acceptors).

	Fentanyl Derivatives	X••B Interaction	ΔE_int_ (kcal/mol)	X••B Distance (Å)	R–X••B Angle (θ)
A	Fentanyl	N_1_	−10.51	2.89	176.1
		O_1_	−7.11	2.82	179.3
B	*a*-Mefentanyl	N_1_	−10.54	2.90	175.9
		O_1_	−7.12	2.82	179.3
C	Crotonylfentanyl	N_1_	−9.29	2.85	177.0
		O_1_	−7.14	2.83	179.3
D	Cyclopropylfentanyl	N_1_	−9.04	2.86	177.3
		O_1_	−7.22	2.81	178.7
E	Mefentanyl	N_1_	−8.62	2.86	175.9
		O_1_	−7.70	2.84	172.6
F	Methoxyacetylfentanyl	N_1_	−9.12	2.85	176.9
		O_1_	−9.02	2.83	176.7
		O_2_	−11.16	2.96	164.5
G	*p*-Fluorobutyrylfentanyl	N_1_	−10.81	2.90	176.0
		O_1_	−7.13	2.83	179.7
H	Ohmefentanyl	N_1_	−12.52	2.97	168.7
		O_1_	−10.07	2.83	178.6
		O_2_	−9.24	2.92	170.2
I	Phenaridine	N_1_	−9.61	2.96	175.7
		O_1_	−10.45	2.82	178.8
J	Valerylfentanyl	N_1_	−9.59	2.86	174.2
		O_1_	−8.63	2.84	173.6
K	*p*-Methoxybutyrylfentanyl	N_1_	−10.23	2.98	162.7
		O_1_	−7.48	2.82	179.3
		O_2_	−4.46	2.92	175.3

## Data Availability

Data are contained within the article and Supplementary Materials.

## Supplementary Materials

The following Supplementary Materials can be downloaded at: https://www.mdpi.com/article/10.3390/nano14110917/s1, Experimental details of the synthesis of Ligand 1 including NMR and voltammetry; TEM and UV–Vis characterization of *f*-MPCs and *unf*-MPCs; DFT results for adducts of IPFB and selected FTN derivatives; Fragmentation schemes for *p*-FBF and *p*-MBF; Table of fragments’ names, structures, and CAS#s; Table of DFT results for adducts of IPFB and selected fragments; NMR titration analyses for selected fragments; Electrochemical results including SAM and mixed SAM electrode modifications, effect ligand exchange time, C6/DT mixed SAM control film exposed to Frag 5A, mixed C6/Ligand 2 SAMs, dependence on exposure to Frag 5A, tracking of MPC film assembly, *unf*-MPC films before/after exposure to Frag 5A (control), effect of polar washes on XB adsorbates, and *f*-MPC films before/after exposure to Frag 2.
