# Supplementary material for: Functionalized Gold Nanoparticles and Halogen Bonding Interactions Involving Fentanyl and Fentanyl Derivatives
Source: Nanomaterials (Basel). 2024 May 23;14(11):917. doi: 10.3390/nano14110917 (PMC11173406; doi:10.3390/nano14110917)
Supplement: Supplementary file 1 [file nanomaterials-14-00917-s001.zip › nanomaterials-3003636-supplementary.pdf]

Supplementary Materials: Functionalized Gold Nanoparticles and XB Interactions Involving Fentanyl and Fentanyl Derivatives

Molly M. Sherard, Jamie S. Kaplan, Jeffrey H. Simpson, Kevin W. Kittredge†, and Michael C. Leopold*

**Department of Chemistry, Gottwald Center for the Sciences, University of Richmond, Richmond, Virginia 23173, United States (Corresponding Author)*

†Department of Chemistry, Joan P. Brock School of Math and Natural Sciences, Virginia Wesleyan College, Virinia Beach, VA 23455, United States

Table of Contents:

- Experimental details of synthesis of Ligand 1 (hexadecafluoro-8-iodooctane-1-thiol) including NMR and electrochemistry (**Figs. SI-1, SI-2**)
- TEM imaging, histogram analysis and UV-Vis spectra of *f*-MPCs and *unf*-MPCs (**Fig. SI-3**)
- DFT-generated, geometry optimized structure adducts of IPFB interacting with fentanyl derivatives via XB (**Figs. SI-4 to SI-12**)
- **Scheme SI-1 and SI-2** – Fragmentation equivalents of Scheme 2 for pFBF and p-MBF
- **Table SI-0** - Listing of all fragments from fentanyl and select fentanyl derivatives
- **Table SI-1:** Interaction Energies (ΔE_{int}), Bond Distances and Bond Angles of XB Adducts of IPFB (XB Donor) with Fragments of Fentanyl, p-MBF and p-FBF (XB Acceptors)
- DFT-generated, geometry optimized structure adducts of IPFB interacting with fentanyl fragments via XB (**Figs. SI-13 to SI-24**)
- NMR Titration Analyses; IPFB titrated with select fragments (Frag 3, Frag 5A, Frag 6, Frag 10) in various solvents (**Figs. SI-25 to SI-30**)
- Voltammetry (C_{dl} and FeCN) - general effect of SAM-modification of an electrode (**Fig. SI-31**)
- C_{dl} voltammetry and data summary: bare gold vs. all mixed SAMs (**Fig. SI-32**)
- C_{dl} of C6 SAMs vs. C6/Ligand1 mixed SAMs as a function of ligand exchange time (**Fig. SI-33**)
- C_{dl} voltammetry: C6/Ligand1 SAM before/after exposure to Frag 5A w/ C6/DT control (**Fig. SI-34**)
- Voltammetry (C_{dl} and FeCN) – C6/Ligand 2 SAM before/after exposure to Frag 5A (**Fig. SI-35**)
- C_{dl} voltammetry: C6/Ligand1 SAM as a function of exposure time to Frag 5A (**Fig. SI-36**)
- C_{dl} voltammetry as a function of dithiol-linked MPC film assembly (**Fig SI-37**)
- Voltammetry (C_{dl} and FeCN) – C6/dithiol-linked *unf*-MPC film assembly before/after exposure to Frag 5A [Control system] (**Fig. SI-38**)
- Voltammetry (C_{dl} and FeCN): Effect of polar "wash" with mixture of MeOH/EtOH/H_2O (**Fig SI-39 to SI-40**)
- C_{dl} voltammetry: C6/Ligand2 SAM, *f*-MPC film, and *unf*-MPC film before/after exposure to Frag 2 [Control system] (**Fig. SI-41**)

- **Synthesis of 1,1,2,2,3,3,4,4,5,5,6,6,7,7,8,8-Hexadecafluoro-8-iodooctane-1-thiol (i.e., hexadecafluoro-8-iodooctane-1-thiol**

Compound was in a modified procedure by Kittredge *et al.* In a 10 mL round bottom flask equipped with a stir bar were added 1.00 g (1.53 mmol) of hexadecafluoro-1,8-diiodooctane in 5 mL of THF. The flask was cooled to -10 °C in a salt ice water bath. To the reaction mixture is added 0.39 mL (1.2 eq., 1.84 mmol) of hexamethyldisilathiane and 1.68 mL (1.1 eq., 1.68 mmol) of 1.0 TBAF in THF. The reaction was allowed to warm to room temperature and stirred overnight. After completion of the reaction, the reaction mixture was diluted with 5 mL diethyl ether and washed once with 10 mL saturated ammonium chloride. The solvent was dried over magnesium sulfate, gravity filtered and removed *in vacuo.* The product mixture was purified flash chromatography by loading the brown oily mixture onto a silica packed vacuum funnel eluted with hexanes. The solvent was removed *in vacuo* leaving a yellowish-brown solid. MP 68-70 °C. ^{1}H NMR (400 MHz, $CDCl_3$) δ 1.55 (s, 1H, S***H***). ^{13}C NMR (75 MHz, $CDCl_3$) δ 108.3, 110.1, 112.5, 115.3, 121.2, 123.6, 129.4. ^{19}F NMR (376 MHz, $CDCl_3$) δ -59.04, -113.03, -120.84, -121.64. HRMS (EOS-TOF) m/z: $[M]^+$ Calcd for $C_8HF_{16}IS$ 559.8588; Found 559.8532..

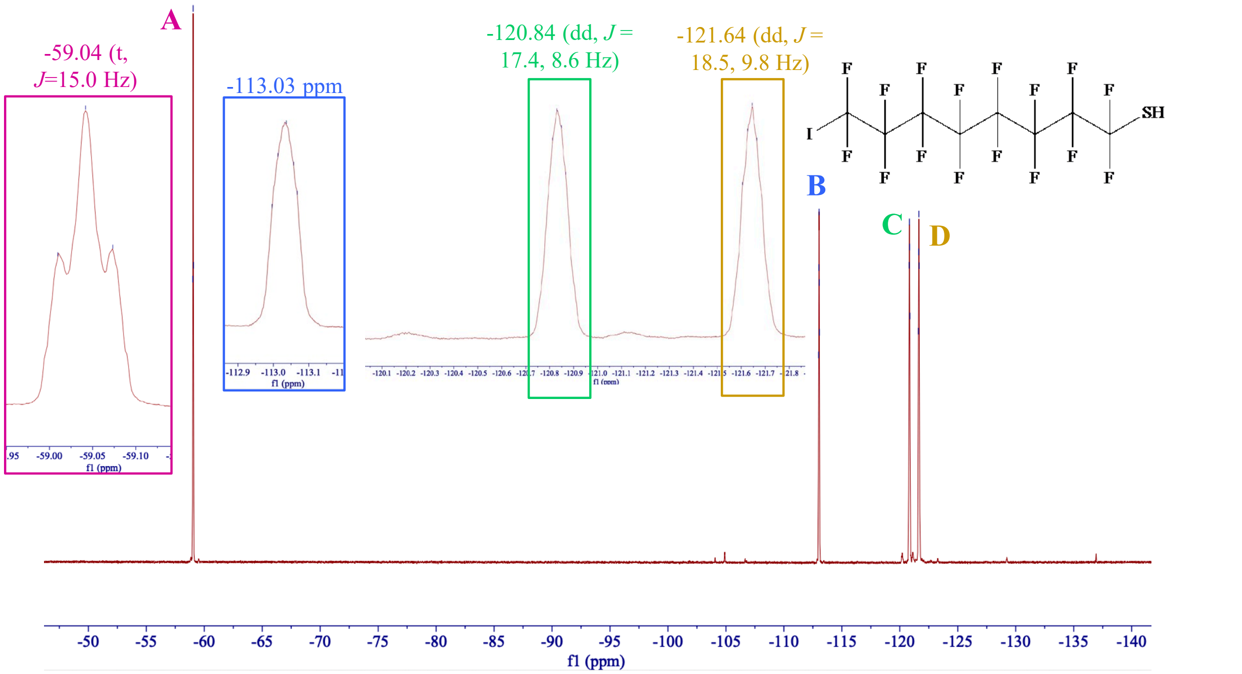

Figure SI-1. ^{19}F NMR of 1,1,2,2,3,3,4,4,5,5,6,6,7,7,8,8-Hexadecafluoro-8-iodooctane-1-thiol or hexadecafluoro-8-iodooctane-1-thiol

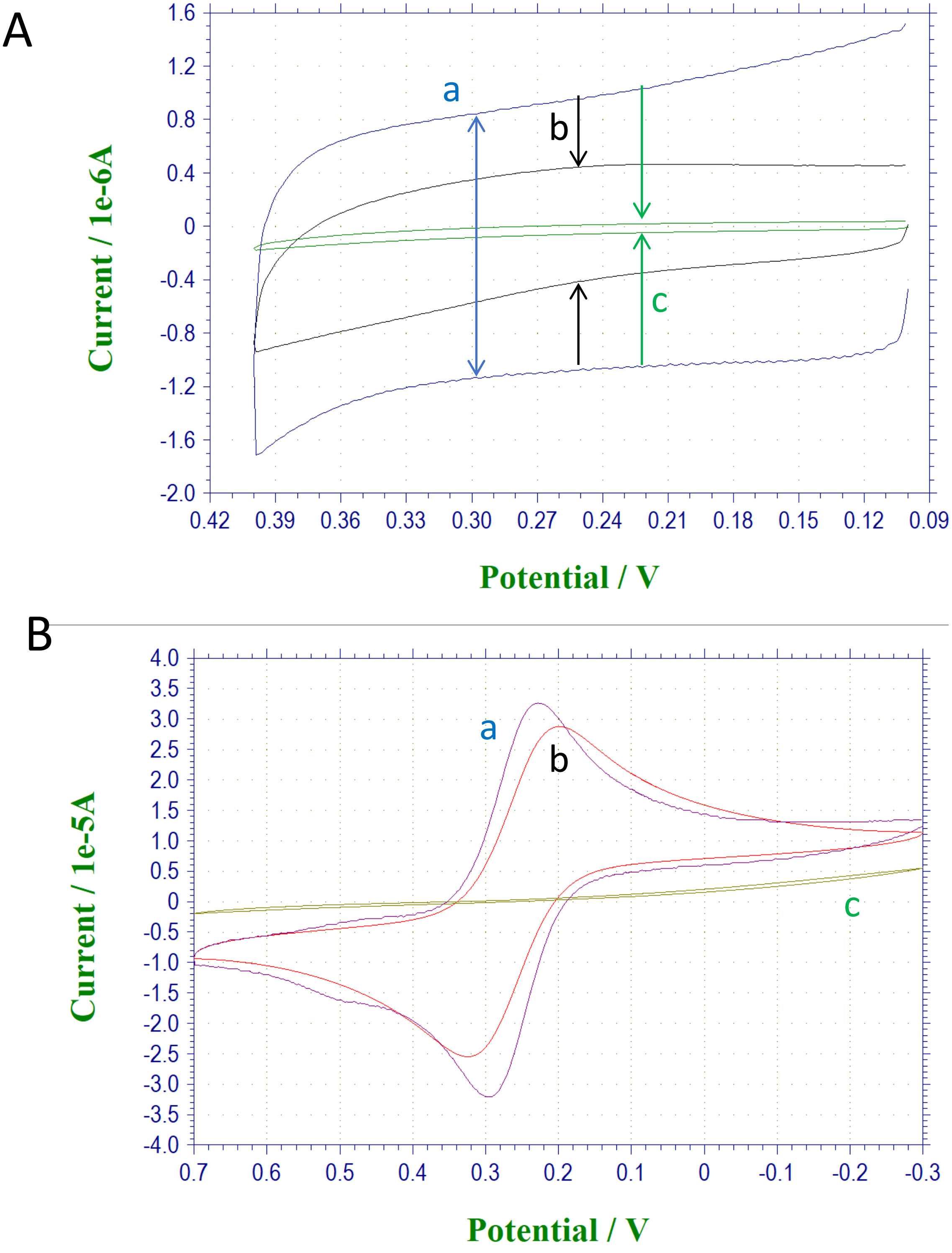

Figure SI-2. (A) Representative CV of **(A)** C_{dl} and **(B)** 5 mM $K_3Fe(CN)_6$ in 0.5 M KCl (100 mV/sec) at **(a)** bare/clean gold, **(b)** Ligand 1 SAM modified gold electrode, and **(c)** an ocatanethiol (C8) modified gold electrode. Notes: All C_{dl} voltammetry was performed in 4.4 mM PBS at 100 mV/sec; results suggest that SAMs formed with Ligand 1 have a greater defect density than similar-sized alkanethiolate SAMs.

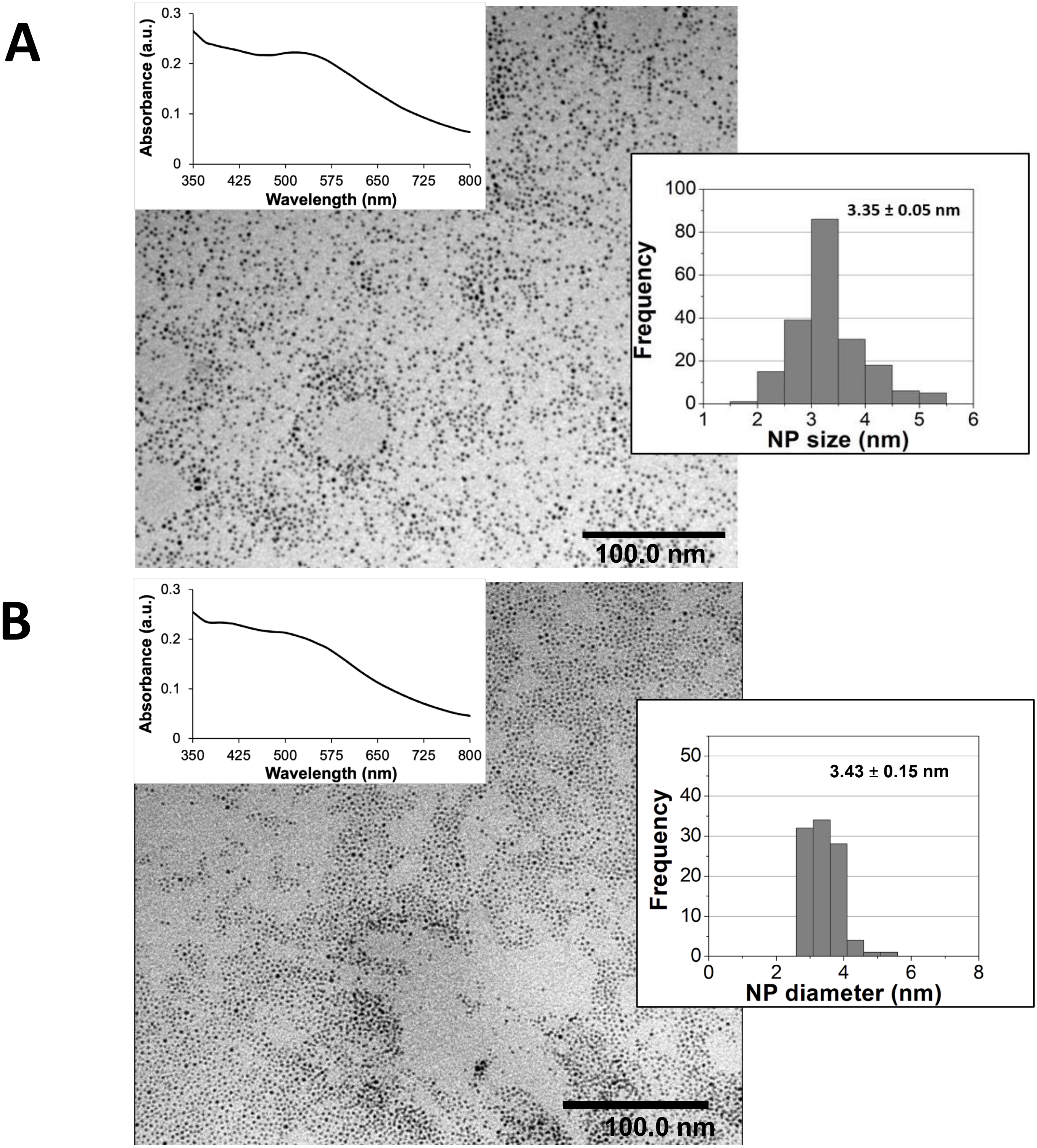

Figure SI-3. Representative TEM images, histogram analysis, and UV-Vis spectra of **(A)** unfunctionalized and **(B)** functionalized MPC nanoparticles. Notes: Histogram analysis of **(A)** ≥ 200 particles per sample or (B) ≥ 100 particles per sample.

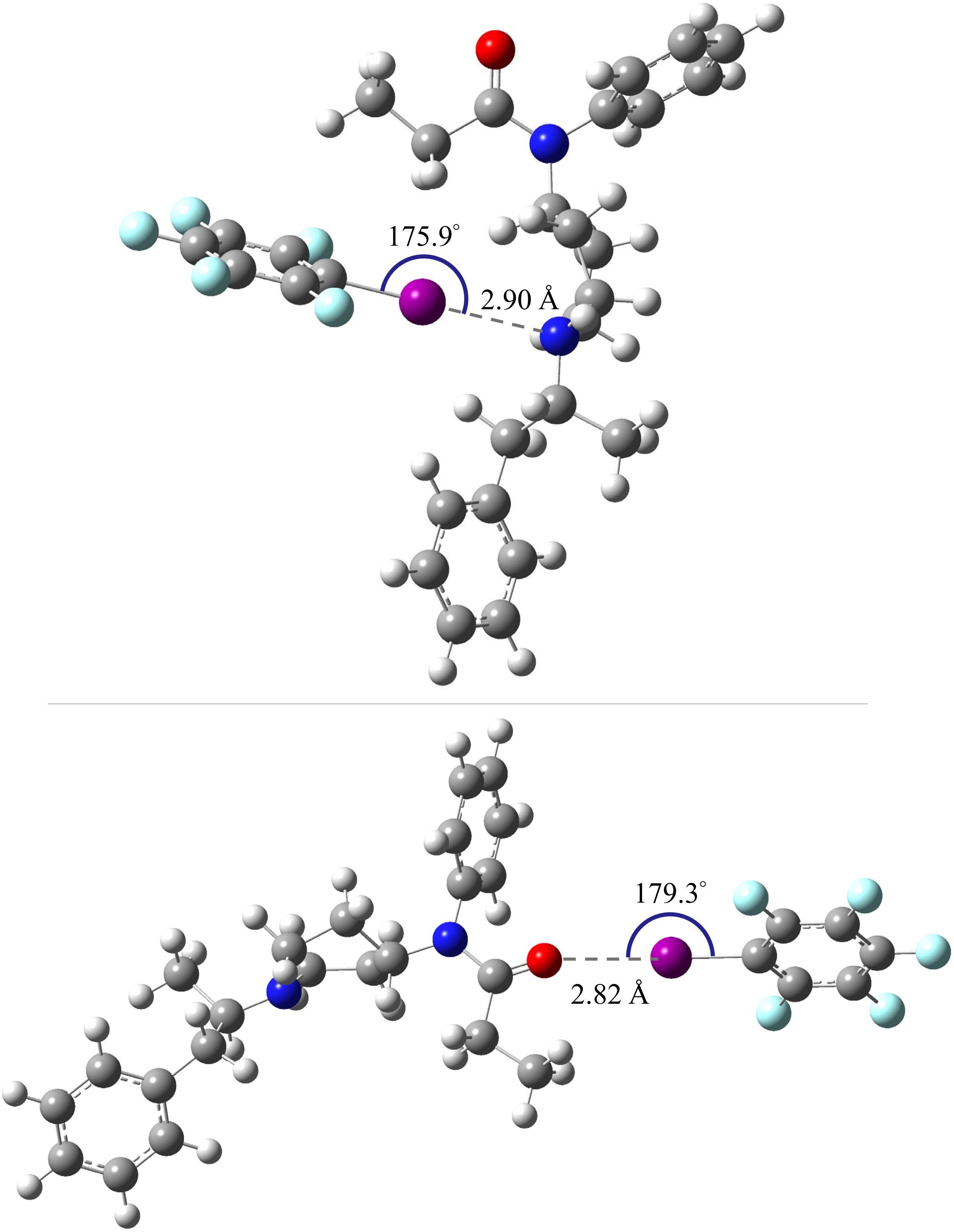

Figure SI-4. DFT-generated, geometry-optimized structure adducts of IPFB interacting with **a-Mefentanyl** at the **N1** (*top*) and **O1** (*bottom*) XB accepting sites.

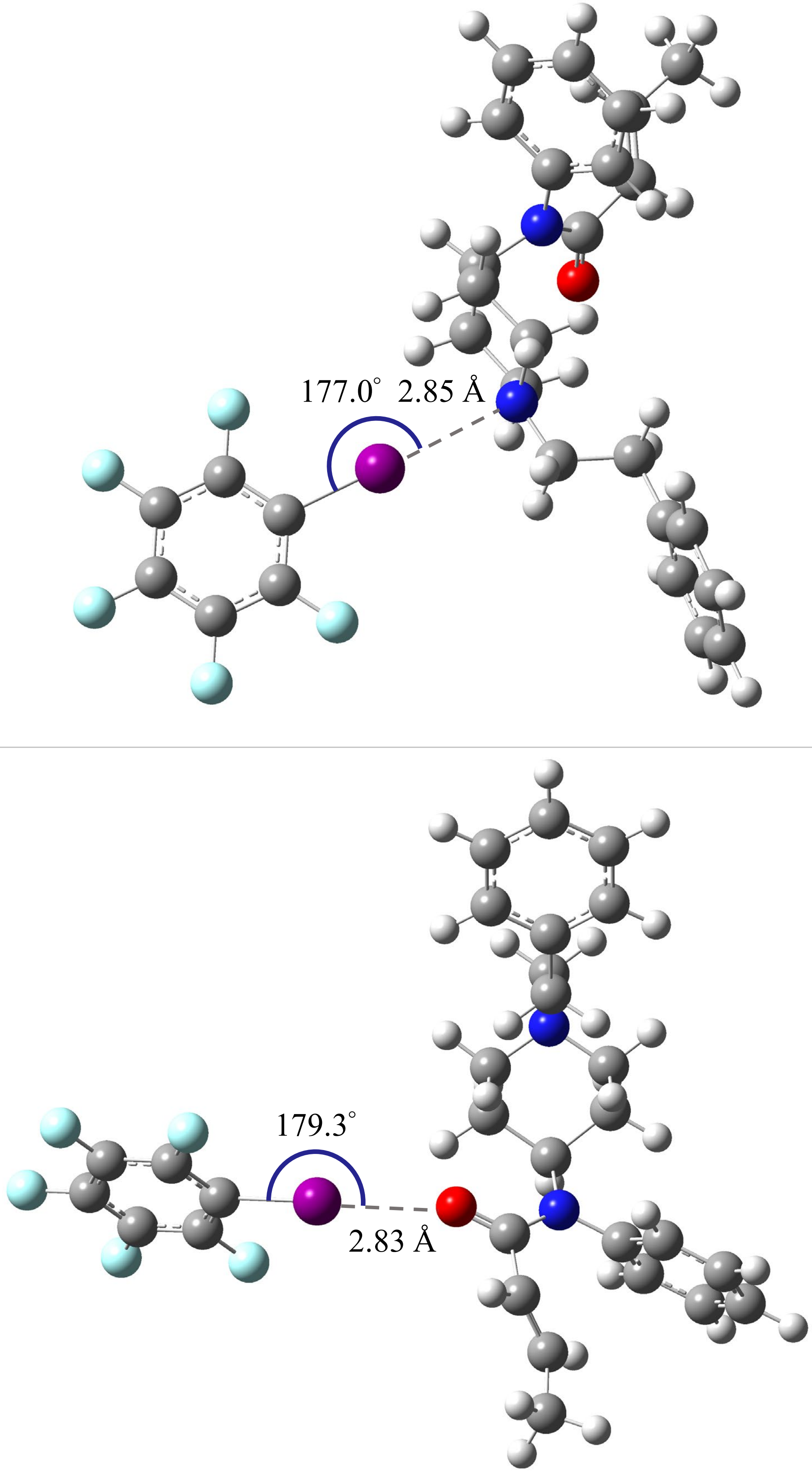

Figure SI-5. DFT-generated, geometry-optimized structure adducts of IPFB interacting with **crotonylfentanyl** at the **N1** (*top*) and **O1** (*bottom*) XB accepting sites.

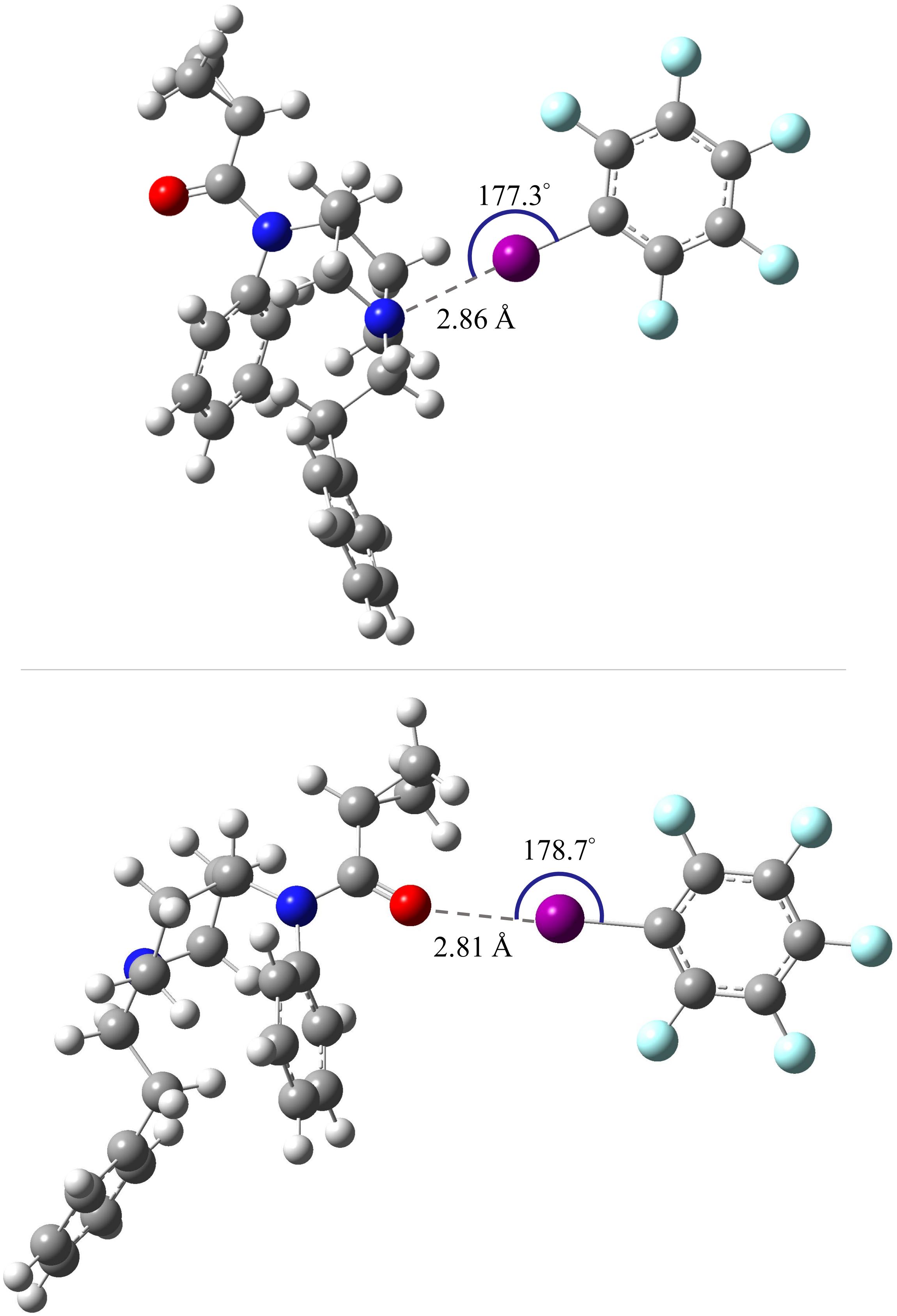

Figure SI-6. DFT-generated, geometry-optimized structure adducts of IPFB interacting with **cyclopropylfentanyl** at the **N1** (*top*) and **O1** (*bottom*) XB accepting sites.

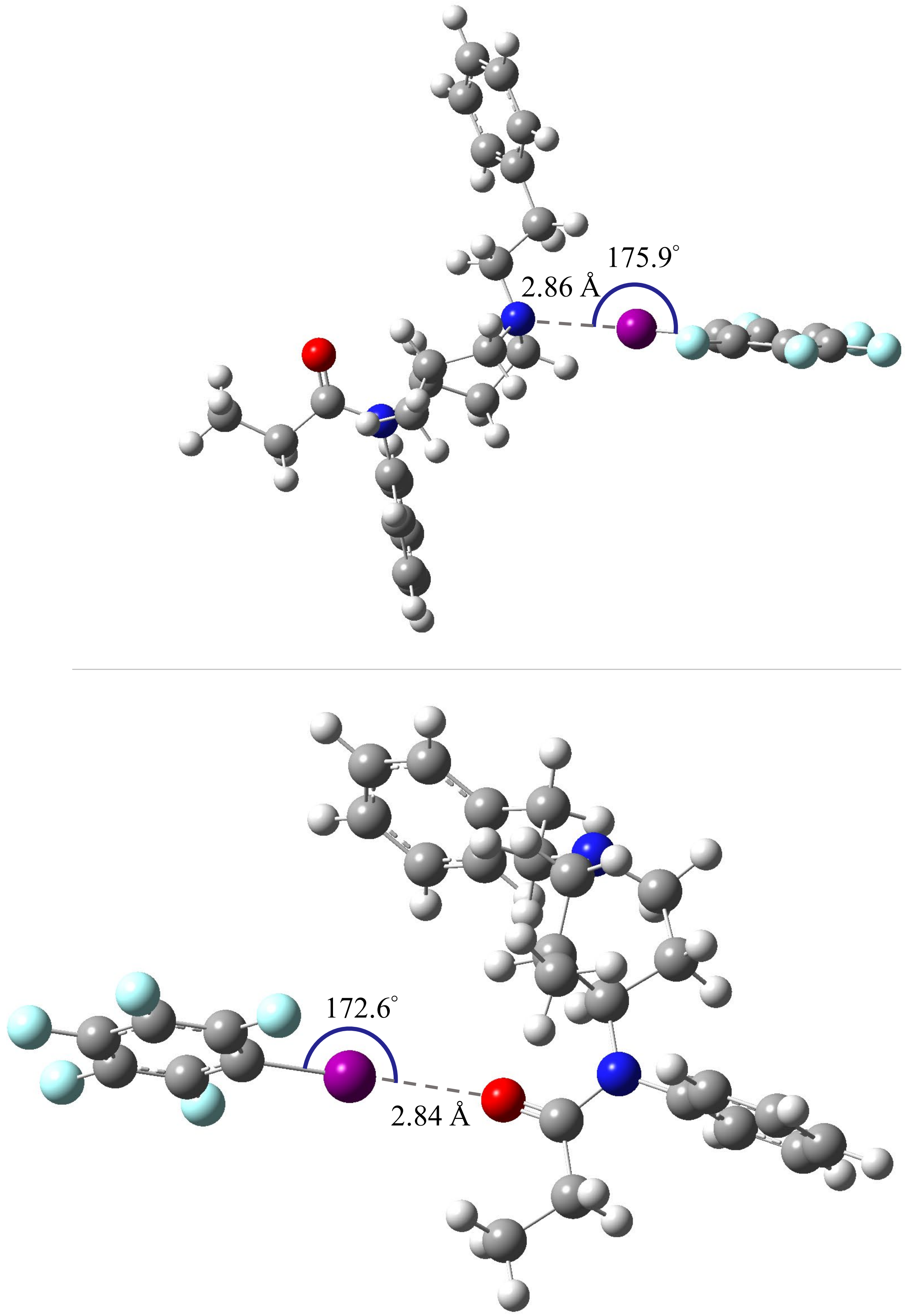

Figure SI-7. DFT-generated, geometry-optimized structure adducts of IPFB interacting with **Mefentanyl** at the **N1** (*top*) and **O1** (*bottom*) XB accepting sites.

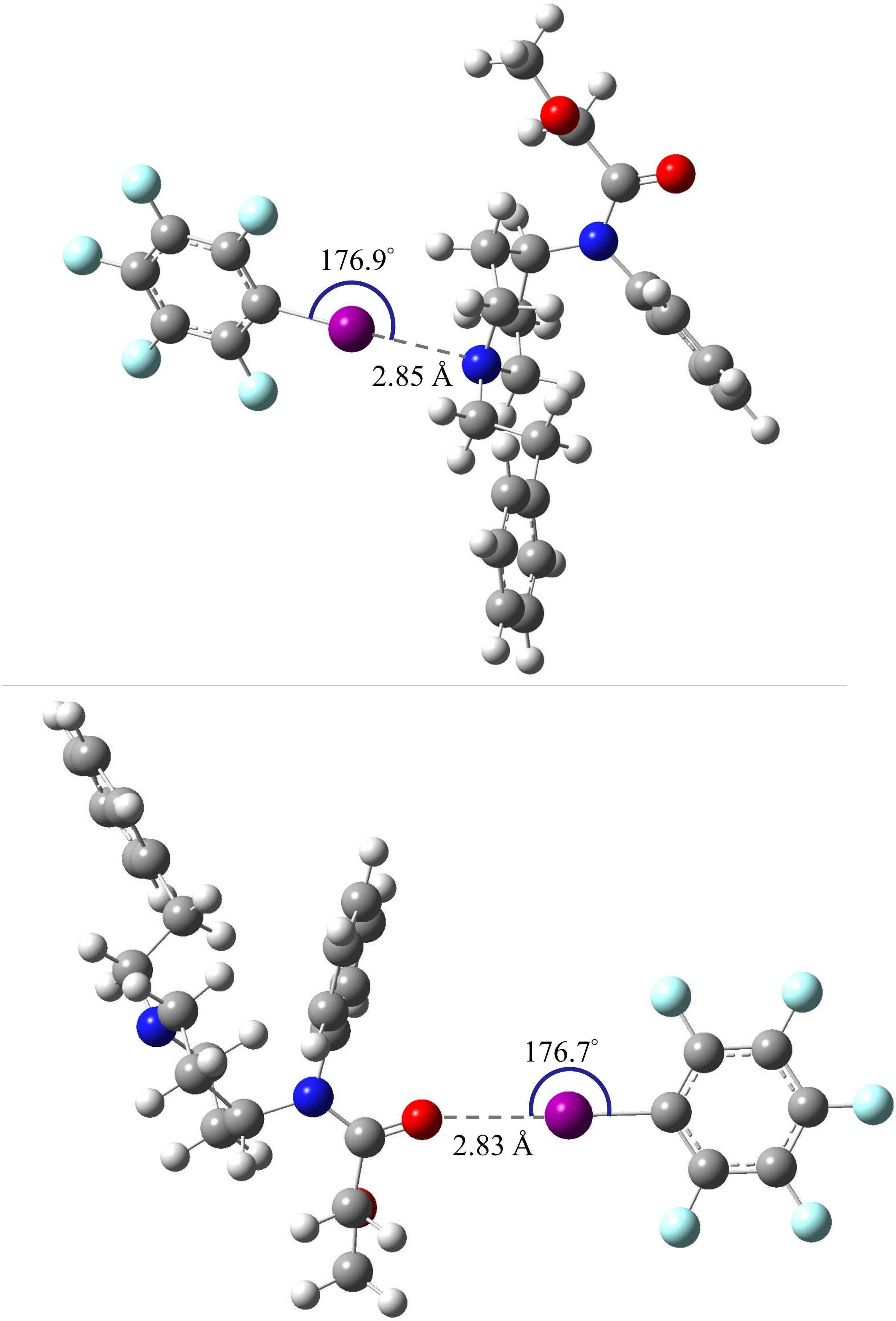

Figure SI-8 (A). DFT-generated, geometry-optimized structure adducts of IPFB interacting with **Methoxyacetylfentanyl** at the **N1** (*top*) and **O1** (*bottom*) XB accepting sites.

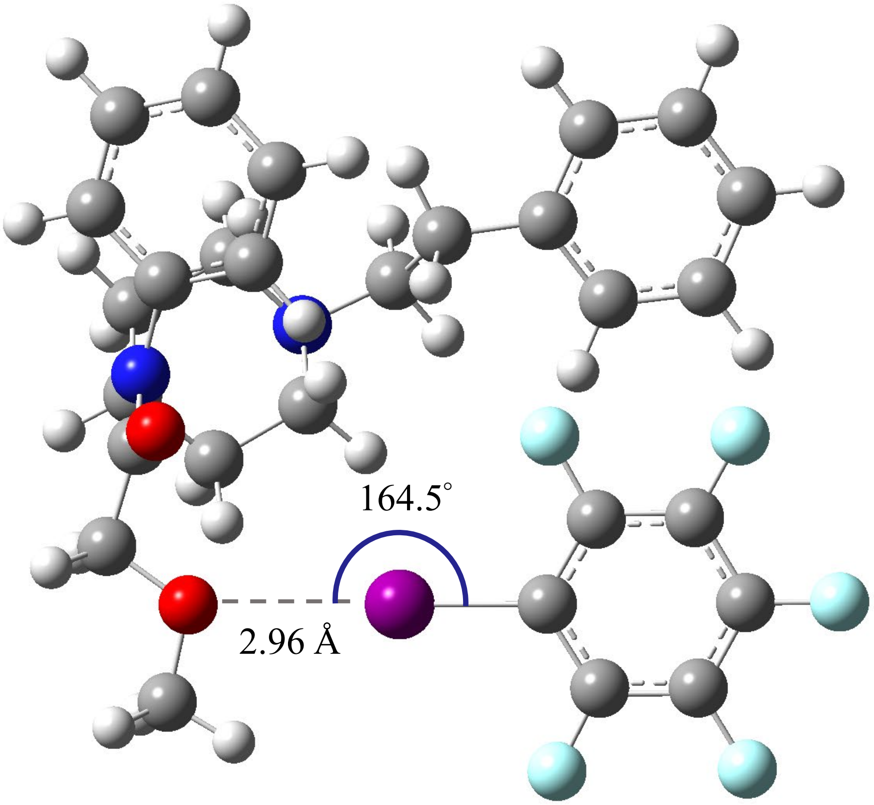

Figure SI-8 (B). DFT-generated, geometry-optimized structure adducts of IPFB interacting with **Methoxyacetylfentanyl** at **O2** XB accepting site.

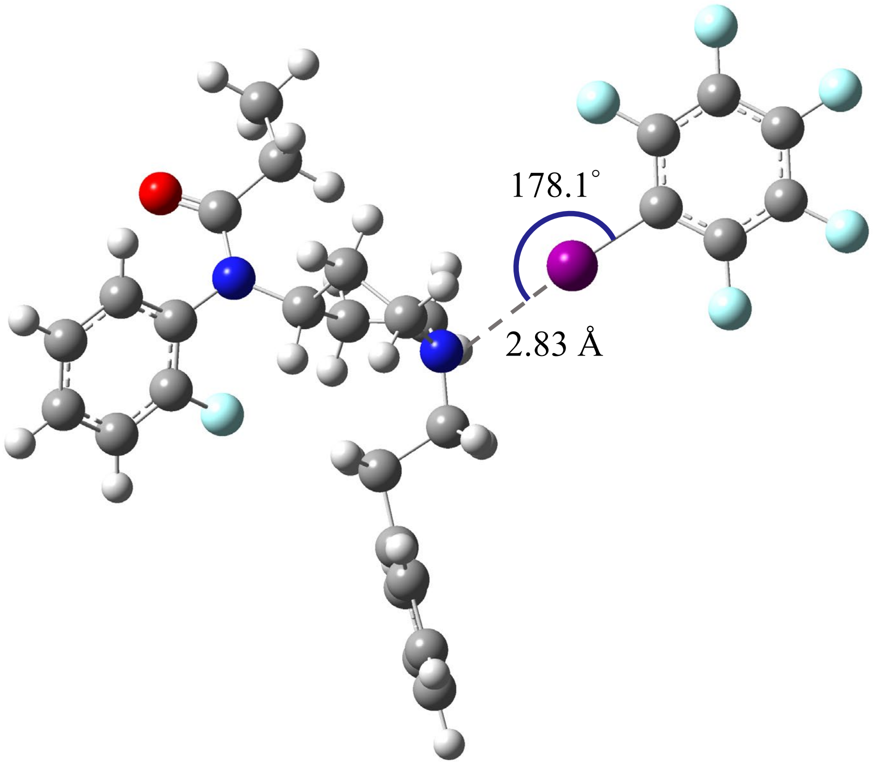

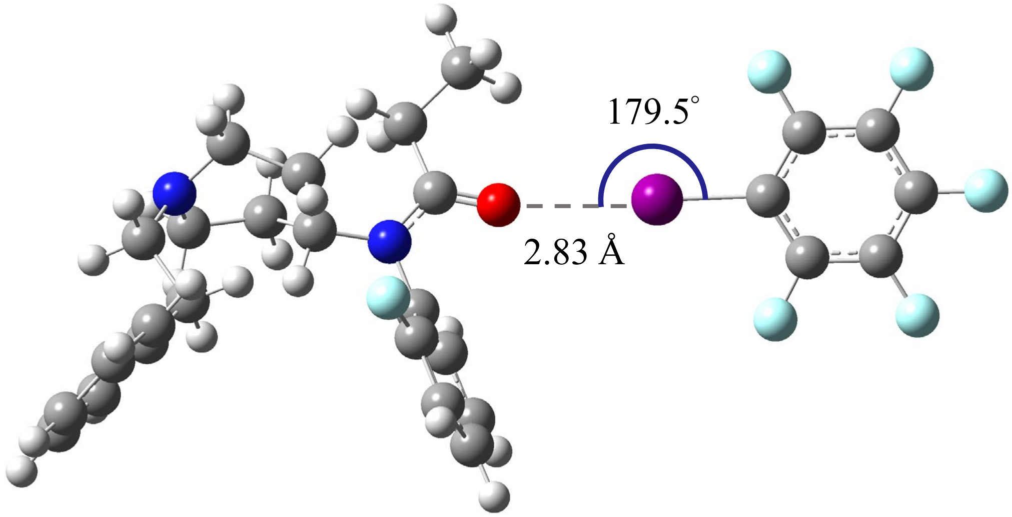

Figure SI-9. DFT-generated, geometry-optimized structure adducts of IPFB interacting with **o-fluorofentanyl** at the **N1** (*top*) and **O1** (*bottom*) XB accepting sites.

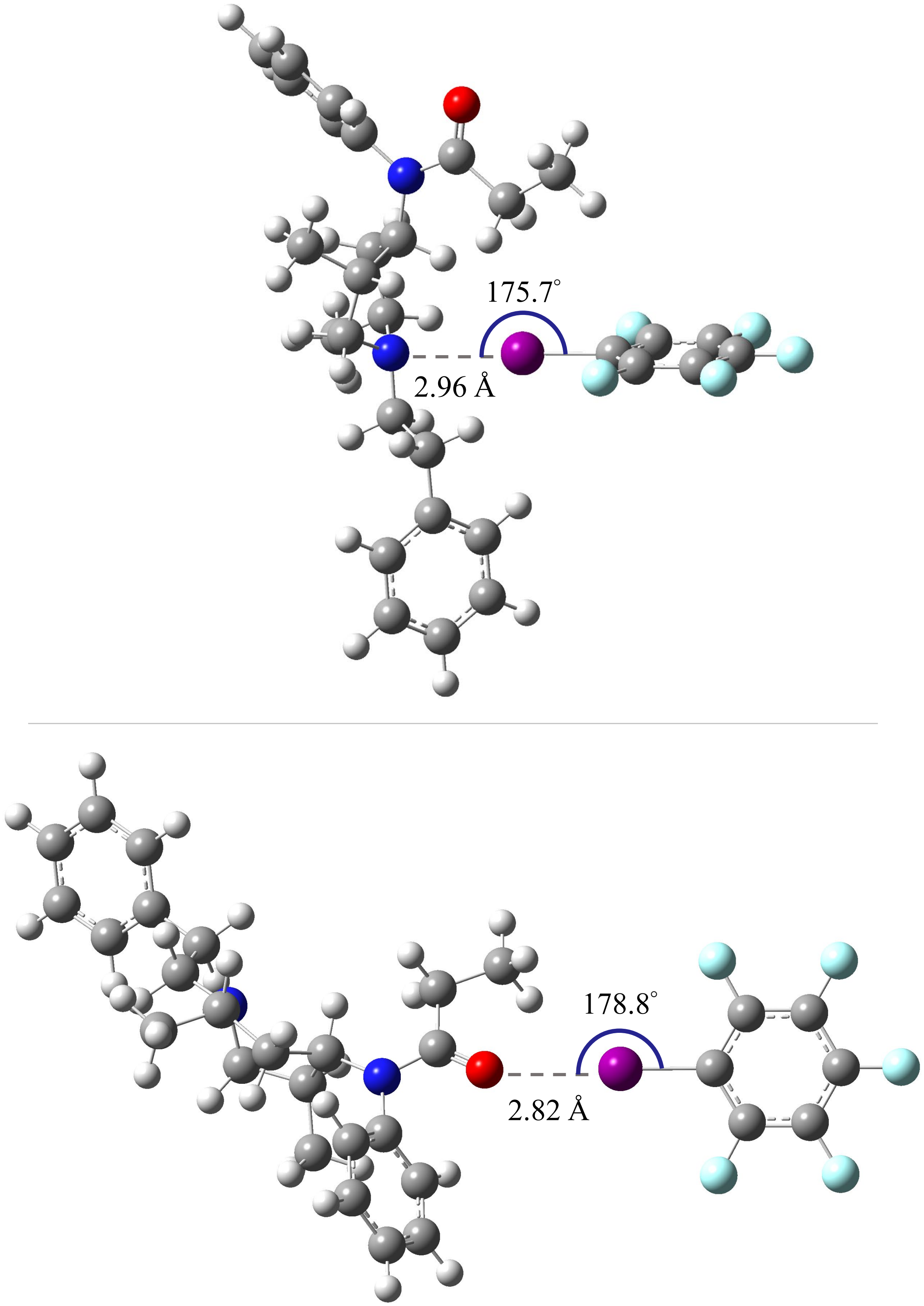

Figure SI-10. DFT-generated, geometry-optimized structure adducts of IPFB interacting with **Phenaridine** at the **N1** (*top*) and **O1** (*bottom*) XB accepting sites.

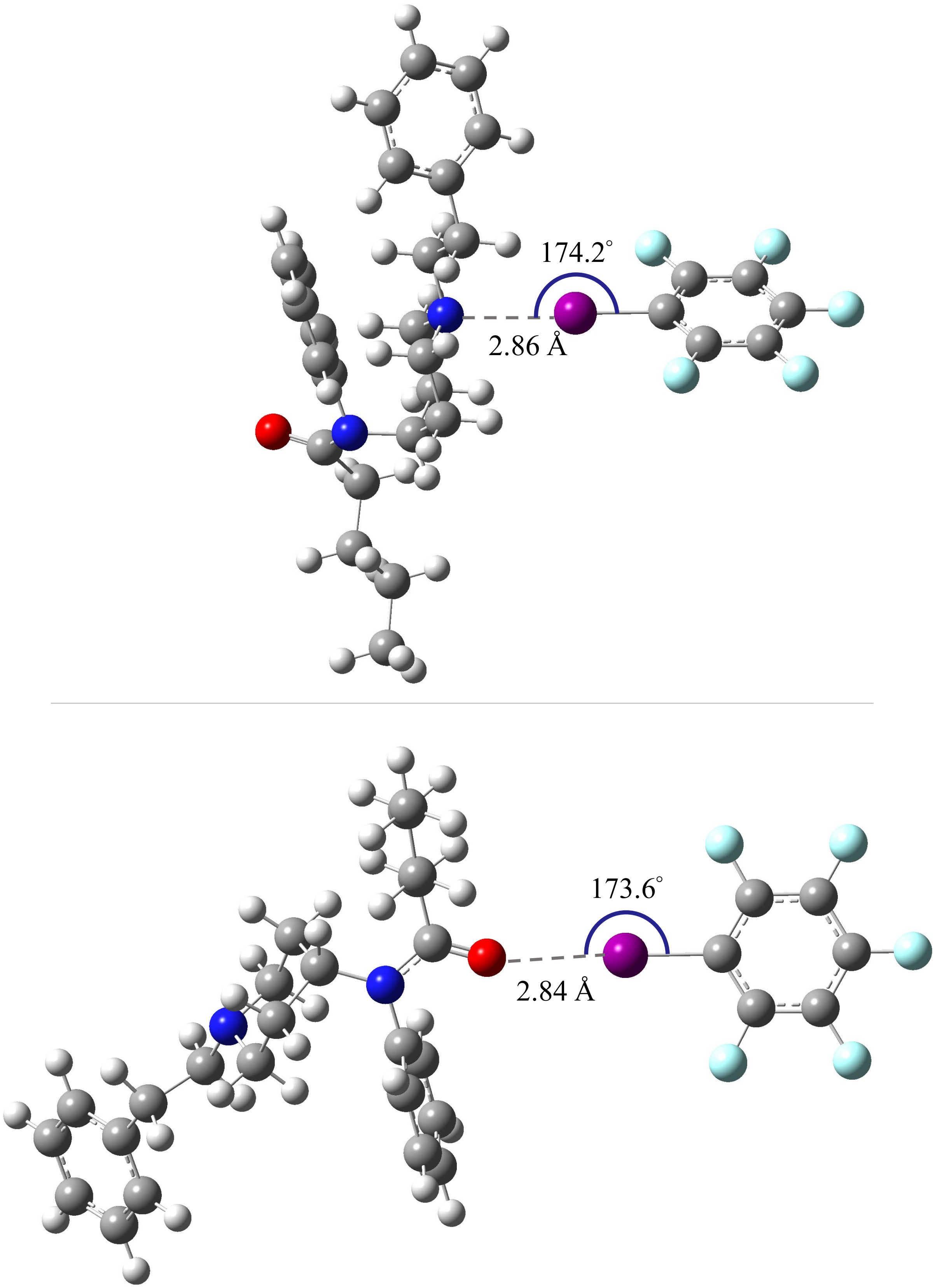

Figure SI-10. DFT-generated, geometry-optimized structure adducts of IPFB interacting with **Valerylfentanyl** at the **N1** (*top*) and **O1** (*bottom*) XB accepting sites.

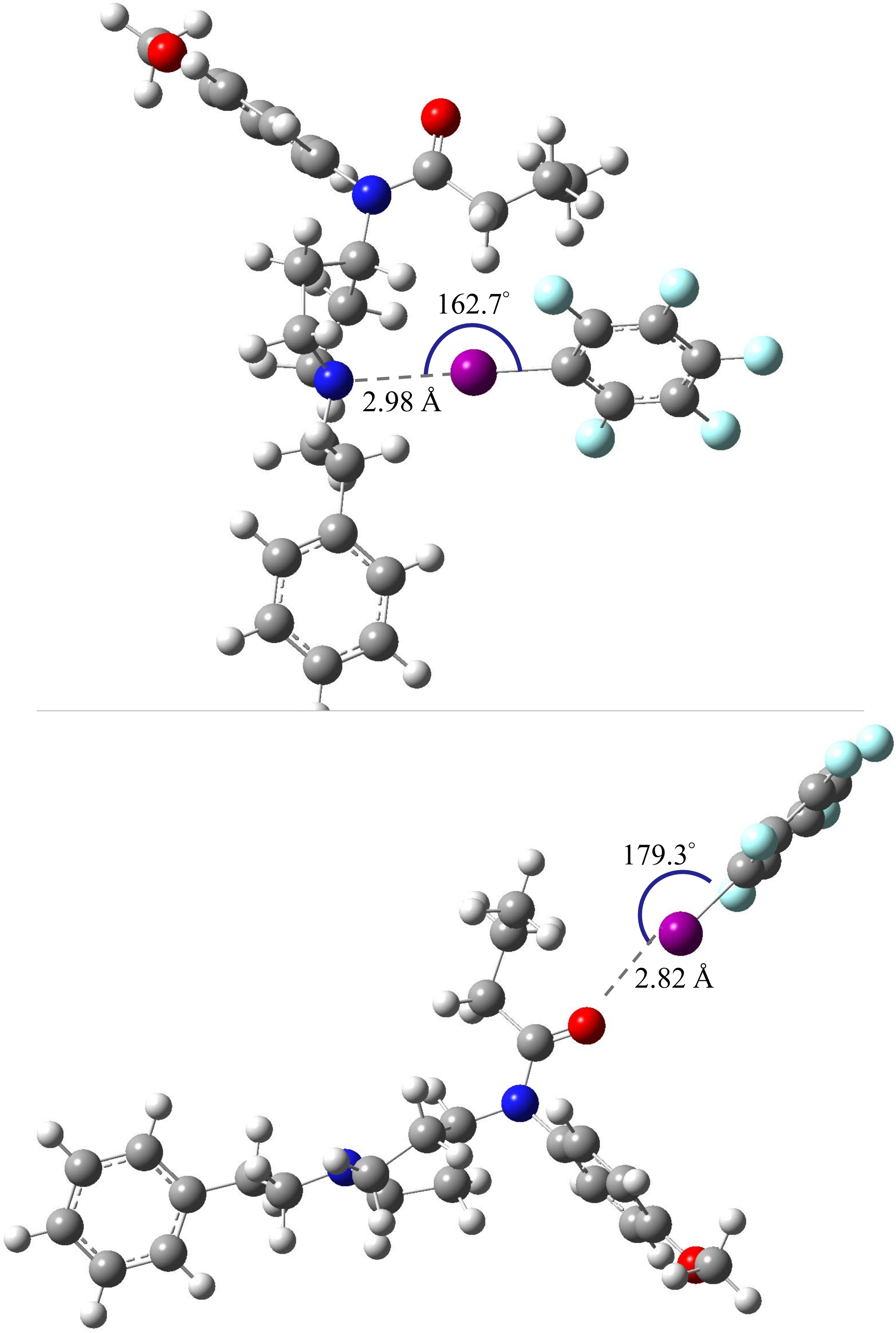

Figure SI-11 (A). DFT-generated, geometry-optimized structure adducts of IPFB interacting with *p*-**Methoxybutyrylfentanyl** at the **N1** (*top*) and **O1** (*bottom*) XB accepting sites.

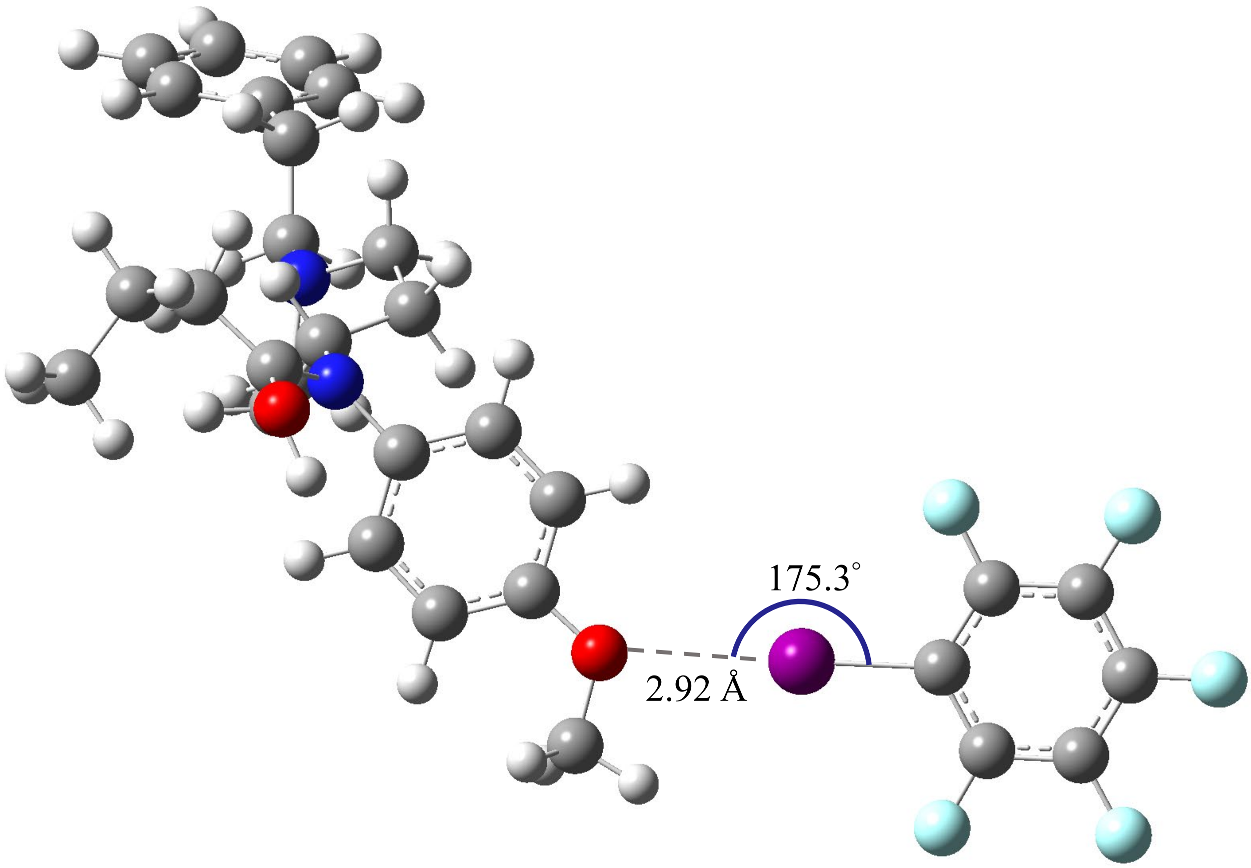

Figure SI-11 (B). DFT-generated, geometry-optimized structure adducts of IPFB interacting with *p*-**Methoxybutyrylfentanyl** at the **N1** (*top*) and **O1** (*bottom*) XB accepting sites.

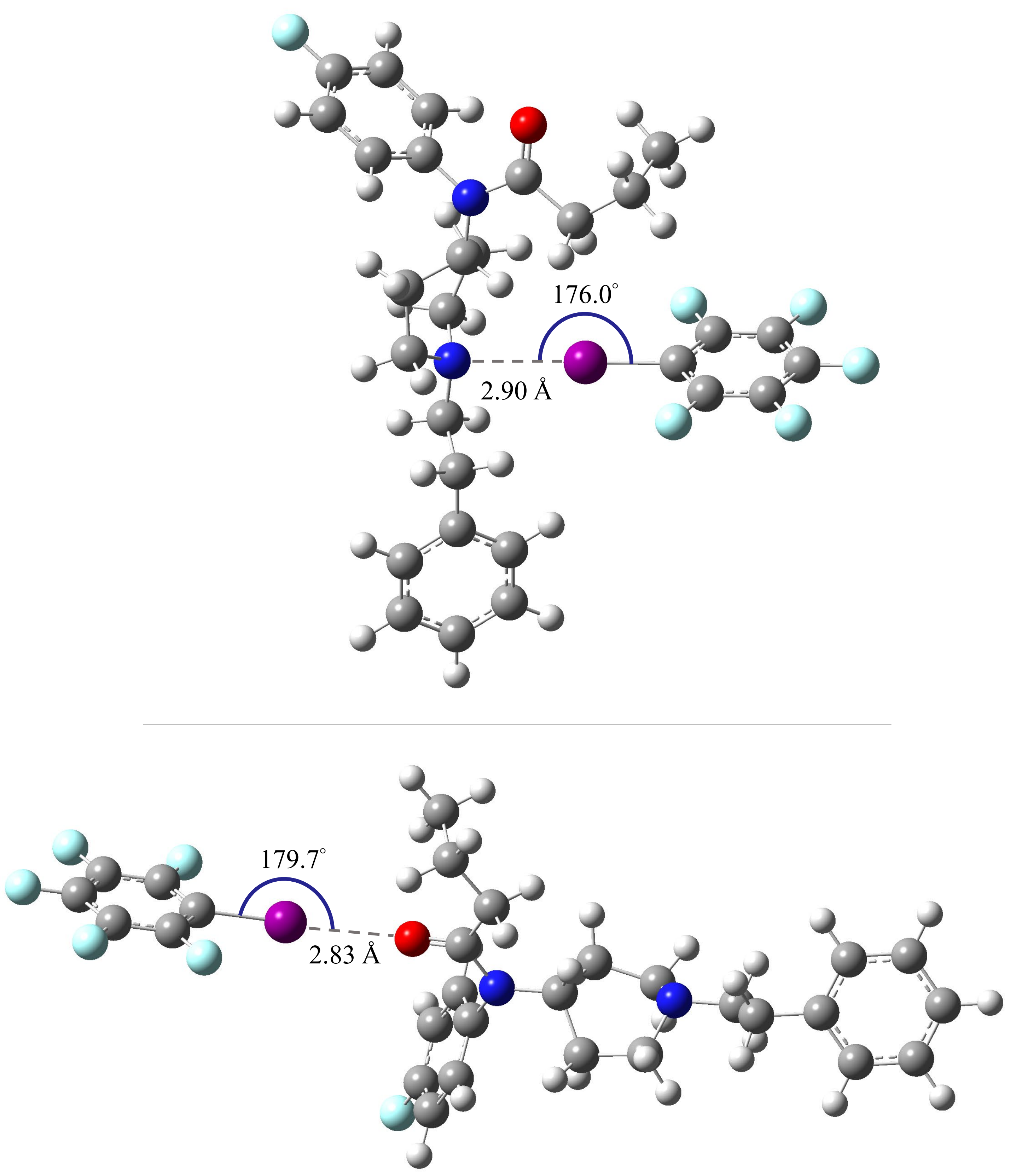

Figure SI-12 (B). DFT-generated, geometry-optimized structure adducts of IPFB interacting with ***p*-Fluorobutyrylfentanyl** at the **N1** (*top*) and **O1** (*bottom*) XB accepting sites.

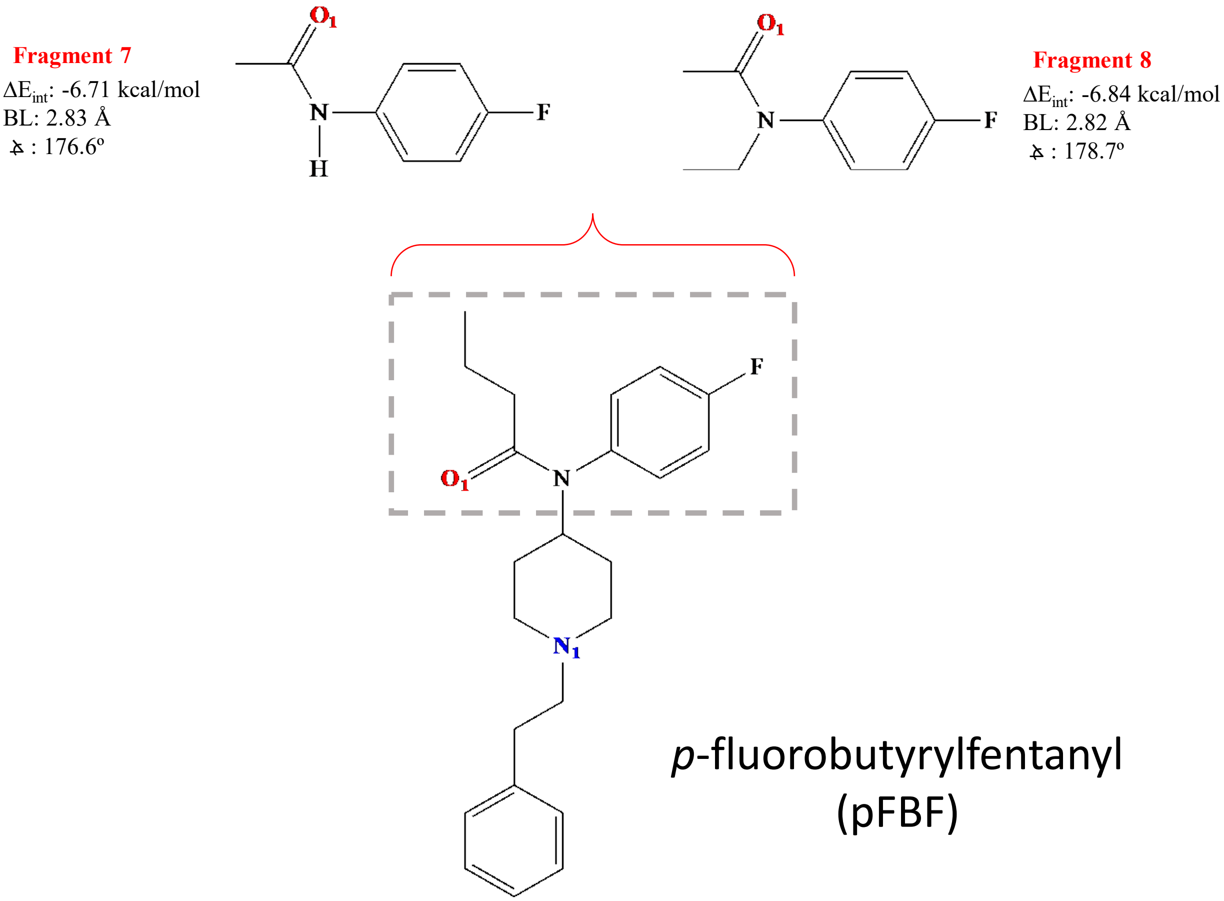

Scheme SI-1. DFT results from analysis of IPFB interacting with specific "fragments" of **pFBF** that emphasize the **O1** accepting site: Fragment 7 (*N*-(4-fluorophenyl)acetamide, Oakwood Chemical) and Fragment 8 (*N*-ethyl-*N*-(4-fluorophenyl)acetamide, not purchased.

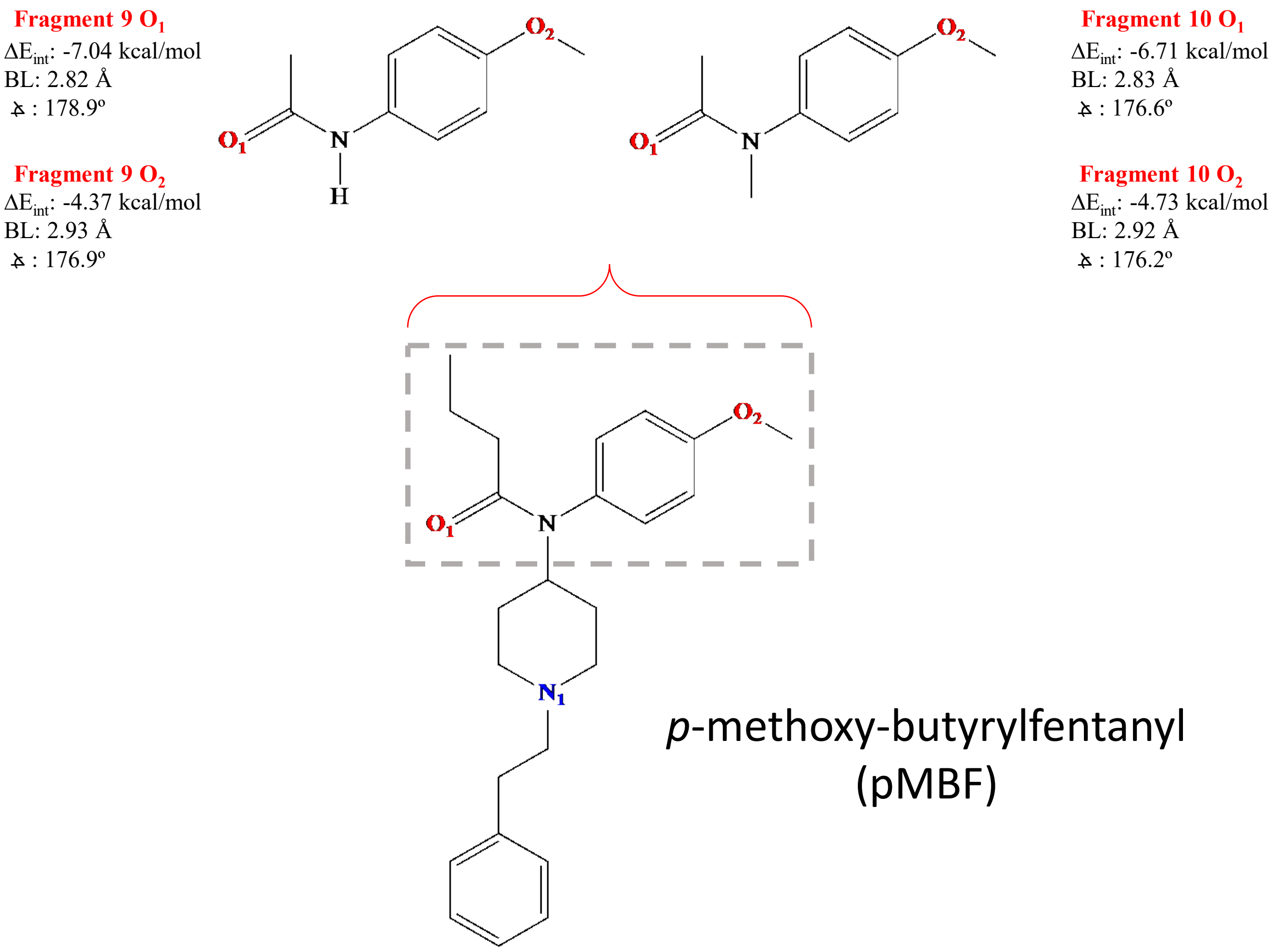

Scheme SI-2. DFT results from analysis of IPFB interacting with specific “fragments” of **pMBF** that emphasize the **O1** and **O2** accepting site: Fragment 9 (*N*-(4-methoxyphenyl)acetamide, not purchased) and Fragment 10 (*N*-(4-methoxyphenyl)-*N*-methylacetamide, Oakwood Chemical.

Table SI-0. Listing of "Fragments" From Fentanyl and Fentanyl Derivatives.

Fragment	IUPAC Name	CAS #	Structure
1	*N*-methyl-*N*-phenylpropanamide	5827-78-1	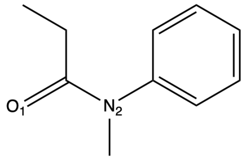
2	*N*-cyclohexyl-*N*-phenylpropanamide	500548-02-7	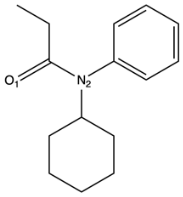
3	*N*-phenyl-*N*-propylacetamide	2437-98-1	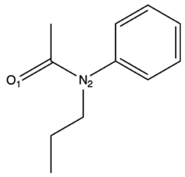
4	*N*-methyl-*N*-phenylbutanamide	42883-79-4	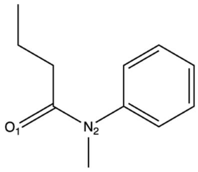
5	1-phenethylpiperidine	6949-43-5	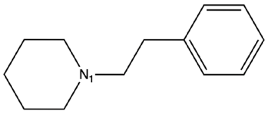
6	*N*-phenylacetamide	103-84-4	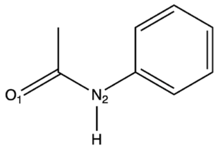
7	*N*-(4-fluorophenyl)acetamide	351-83-7	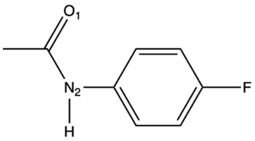
8	*N*-ethyl-*N*-(4-fluorophenyl)acetamide	388078-36-2	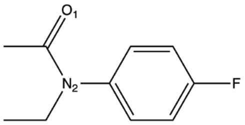
9	*N*-(4-methoxyphenyl)acetamide	51-66-1	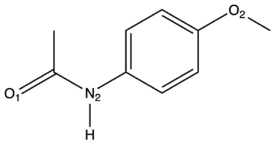

Table SI-1. M06-2X/cc-pVTZ/M06-2X/cc-pVDZ Interaction Energies (ΔE_{int}), Bond Distances (XBD), and Bond Angles of XB Adducts of XB Donor IPFB with Specific Fragments of Fentanyl, p-methoxybutrylfentanyl (p-MBF) and p-fluorobutyrlfentanyl (p-FBF) (XB Acceptors).

Base Structure	Fragments	X••B Interaction	ΔE_{int} (kcal/mol)	X••B Distance (Å)	R–X••B Angle (θ)
A	Fragment 1	O_1	-7.95	2.89	179.3
A	Fragment 2	O_1	-9.49	2.83	179.0
A	Fragment 3	O_1	-7.16	2.81	179.4
A	Fragment 4	O_1	-6.69	2.83	178.6
A	Fragment 5	N_1	-9.82	2.81	175.9
A	Fragment 6	O_1	-6.96	2.83	175.5
L	Fragment 7	O_1	-6.71	2.83	176.6
L	Fragment 8	O_1	-6.84	2.82	178.7
K	Fragment 9	O_1	-7.04	2.82	178.9
		O_2	-4.37	2.93	176.9
K	Fragment 10	O_1	-7.02	2.82	179.1
		O_2	-4.73	2.92	176.2

Notes: Base structures: A = fentanyl (Scheme 2); L = *p*-fluorobutyrylfentanyl (Scheme SI-1); K = *p*-methoxy-butyrylfentanyl (Scheme SI-2).

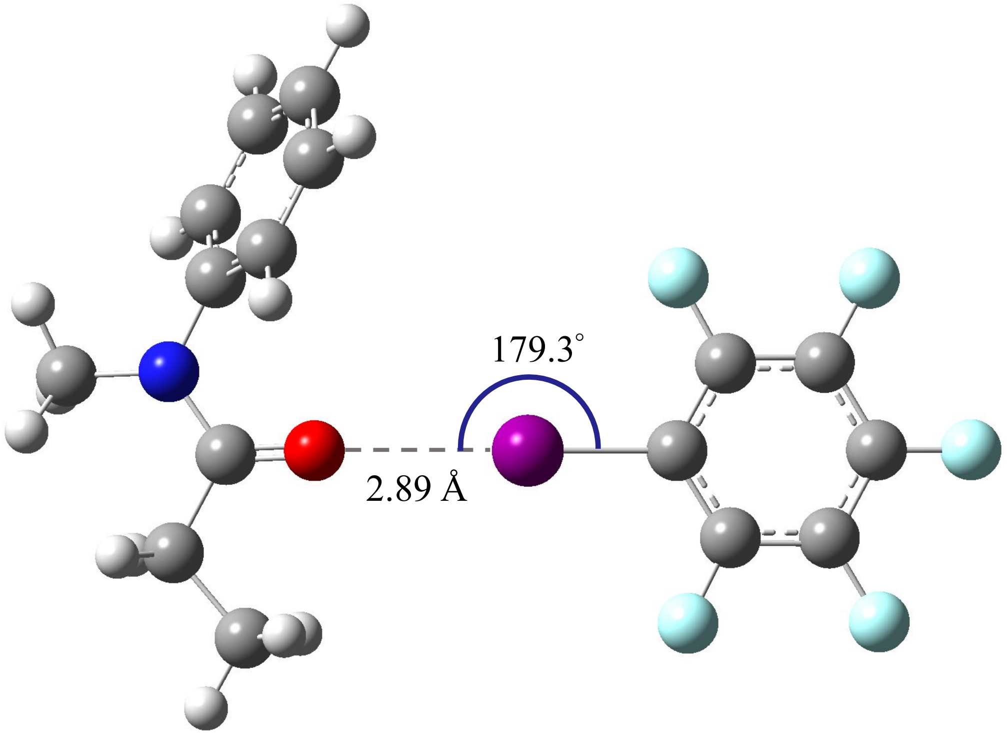

Figure SI-13. DFT-generated, geometry-optimized structure adducts of IPFB interacting with **Fragment 1 (***N*-methyl-*N*-phenylpropanamide**)** at the **O1** XB accepting site.

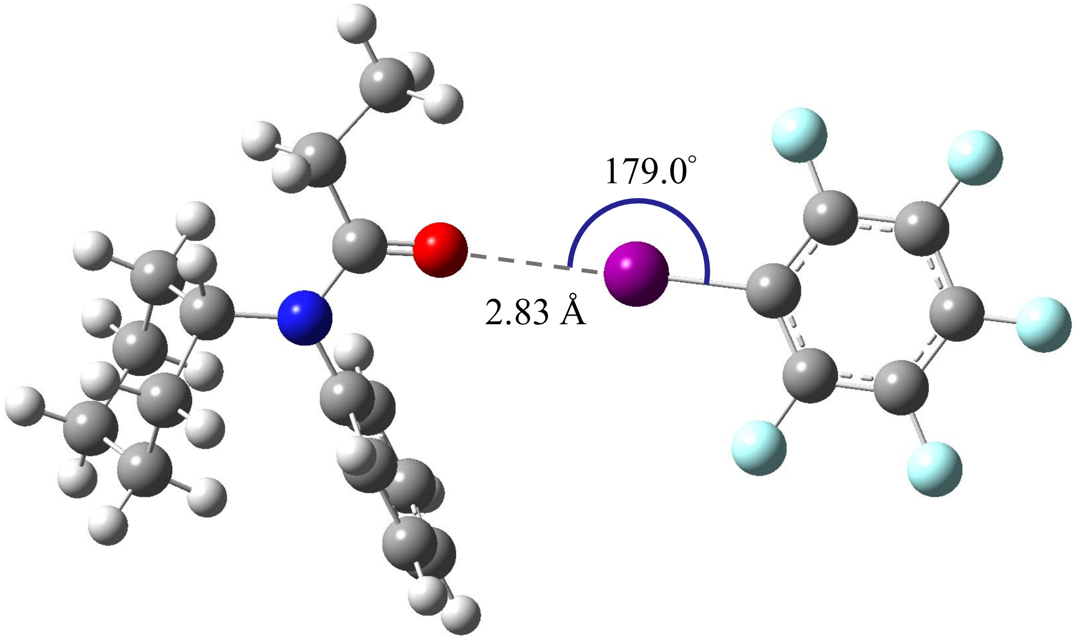

Figure SI-14. DFT-generated, geometry-optimized structure adducts of IPFB interacting with **Fragment 2 (***N*-cyclohexyl-*N*-phenylpropanamide**)** at the **O1** XB accepting site.

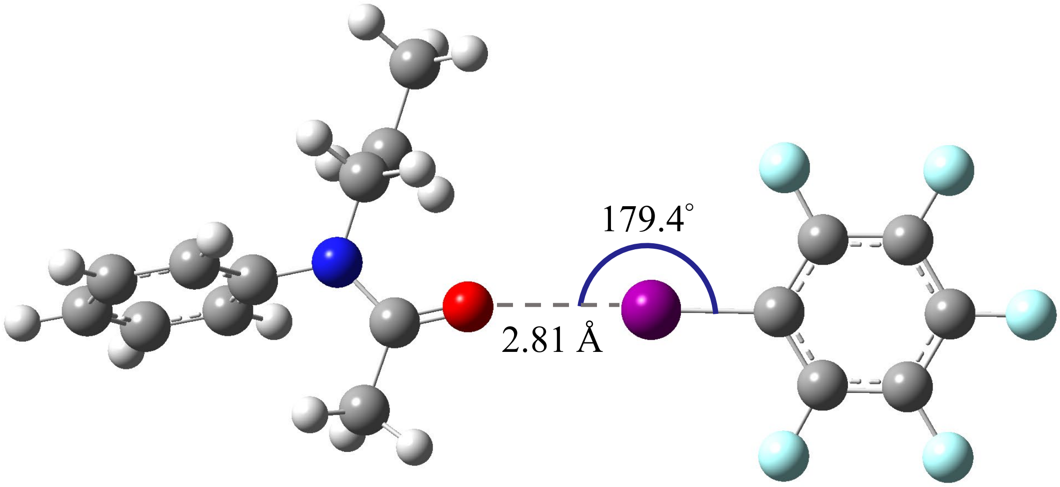

Figure SI-15. DFT-generated, geometry-optimized structure adducts of IPFB interacting with **Fragment 3 (***N*-phenyl-*N*-propylacetamide**)** at the **O1** XB accepting site.

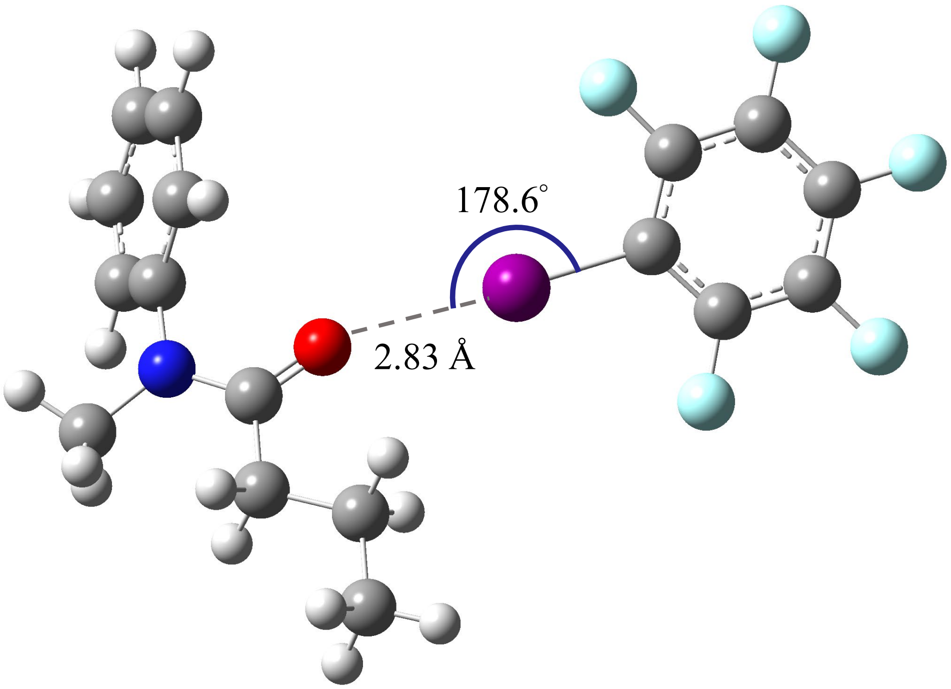

Figure SI-16. DFT-generated, geometry-optimized structure adducts of IPFB interacting with **Fragment 4 (***N*-methyl-*N*-phenylbutanamide**)** at the **O1** XB accepting site.

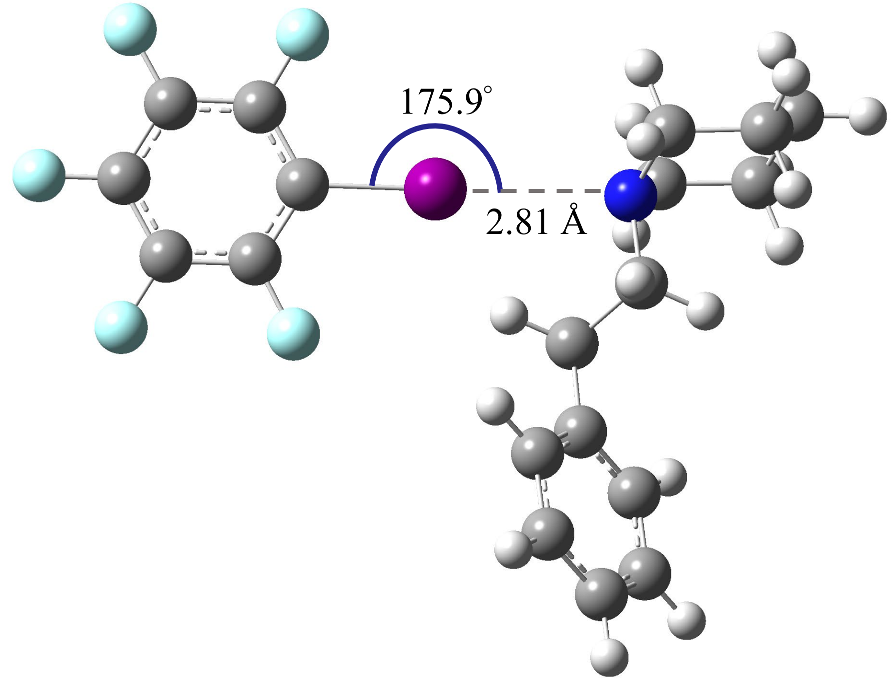

Figure SI-17. DFT-generated, geometry-optimized structure adducts of IPFB interacting with **Fragment 5 (**1-phenethylpiperidine**)** at the **O1** XB accepting site.

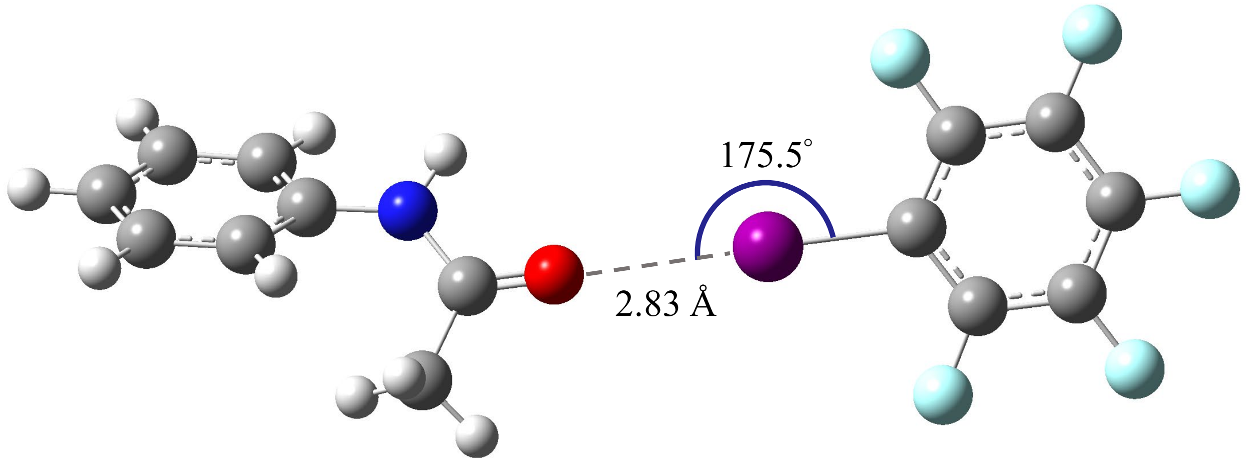

Figure SI-18. DFT-generated, geometry-optimized structure adducts of IPFB interacting with **Fragment 6 (***N*-phenylacetamide**)** at the **O1** XB accepting site.

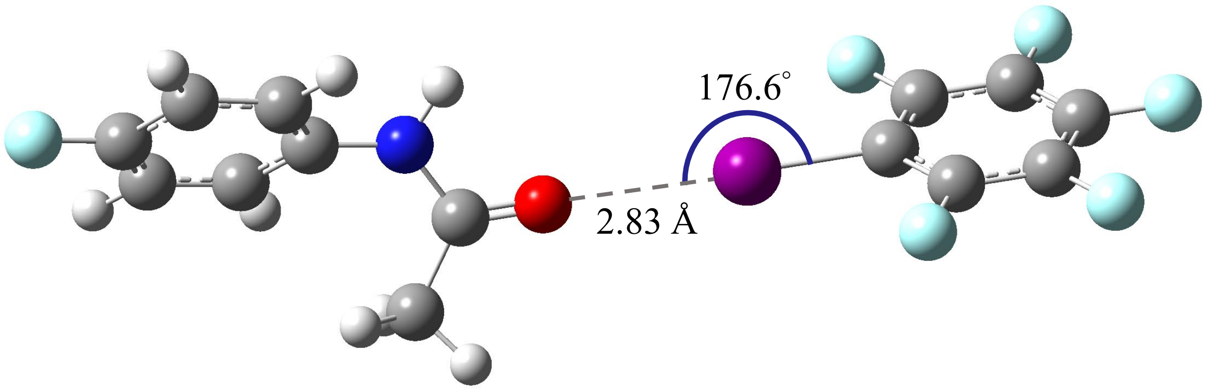

Figure SI-19. DFT-generated, geometry-optimized structure adducts of IPFB interacting with **Fragment 7 (***N*-(4-fluorophenyl)acetamide**)** of p-FBF at the **O1** XB accepting site.

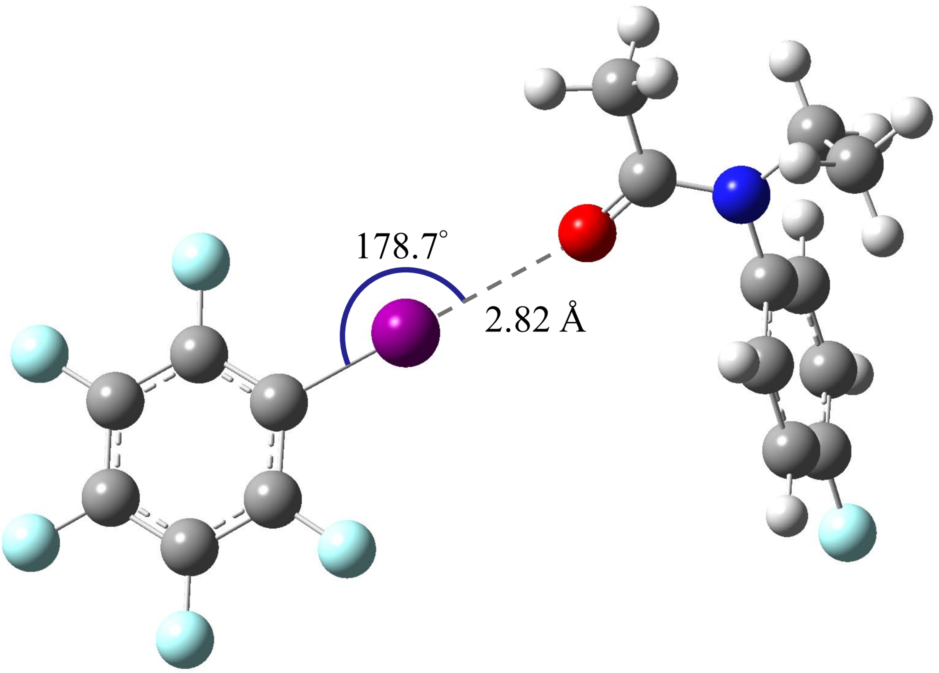

Figure SI-20. DFT-generated, geometry-optimized structure adducts of IPFB interacting with **Fragment 8 (***N*-ethyl-*N*-(4-fluorophenyl)acetamide**)** of p-FBF at the **O1** XB accepting site.

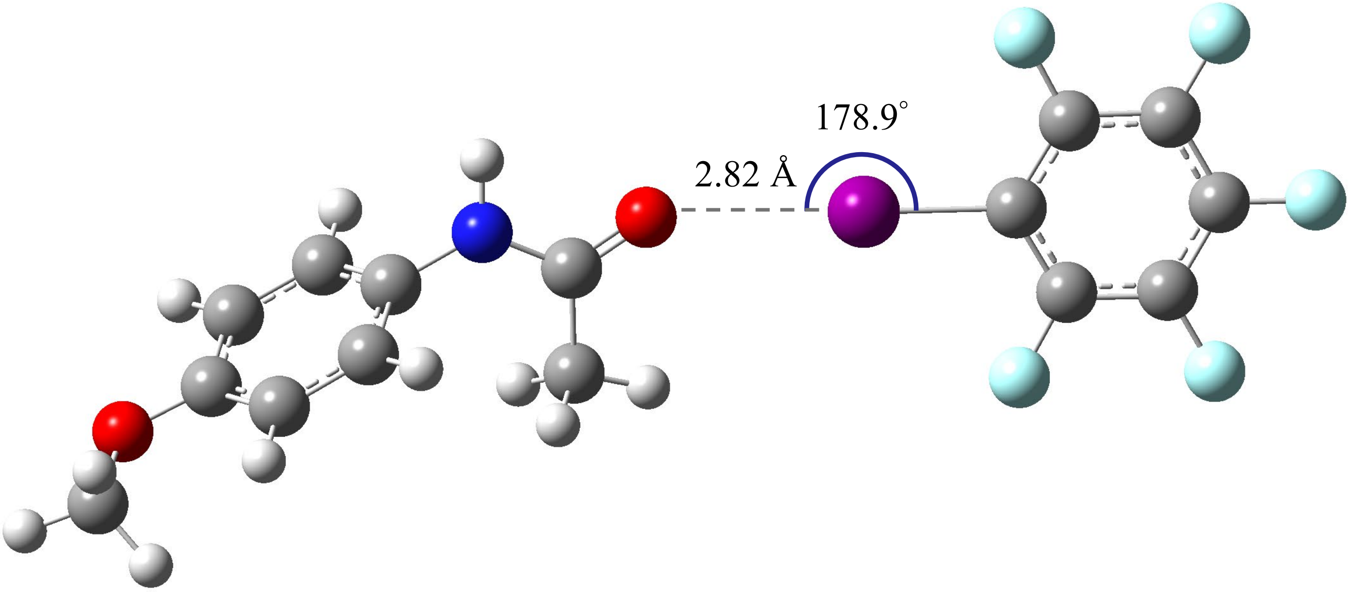

Figure SI-21. DFT-generated, geometry-optimized structure adducts of IPFB interacting with **Fragment 9 (***N*-(4-methoxyphenyl)acetamide**)** from pMBF (Scheme SI-2) at the **O1** XB accepting site.

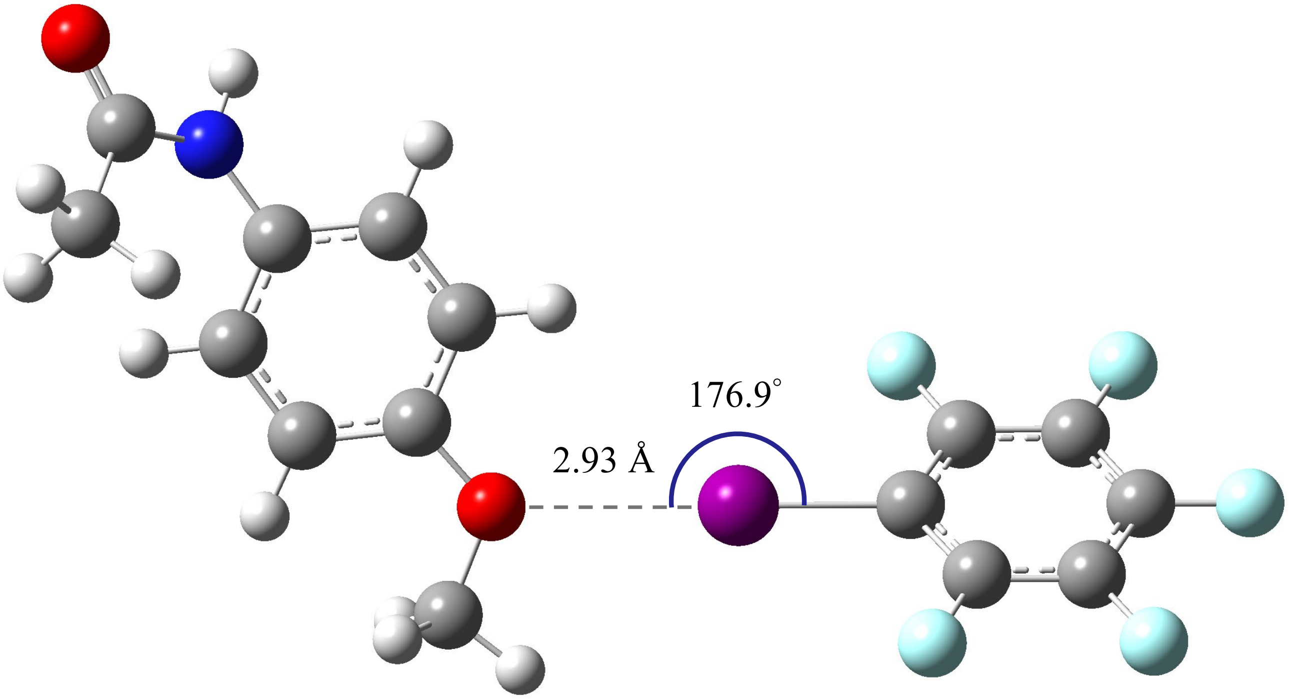

Figure SI-22. DFT-generated, geometry-optimized structure adducts of IPFB interacting with **Fragment 9 (***N*-(4-methoxyphenyl)acetamide**)** from pMBF (Scheme SI-2) at the **O2** XB accepting site.

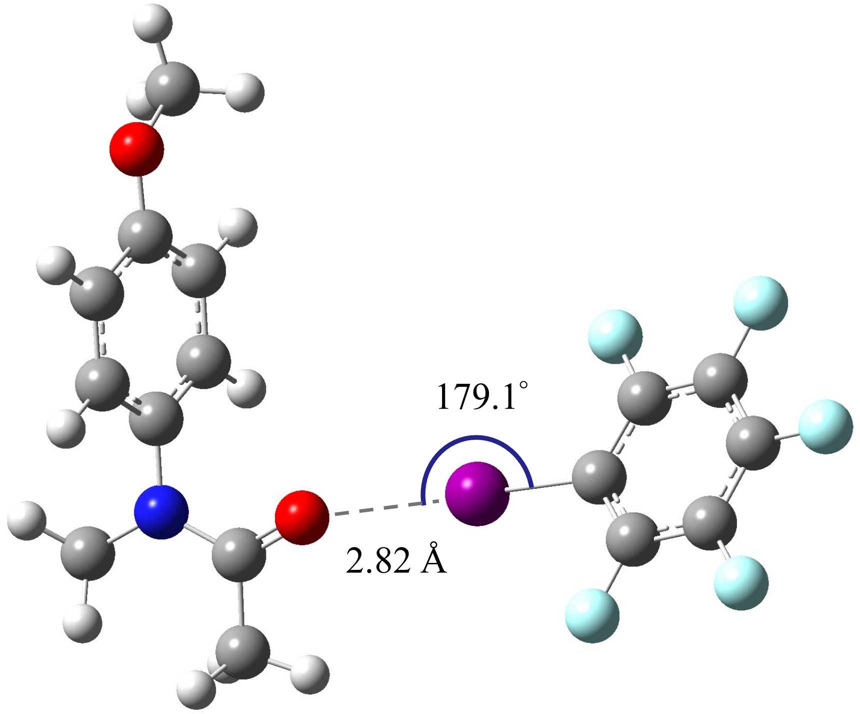

Figure SI-23. DFT-generated, geometry-optimized structure adducts of IPFB interacting with **Fragment 10 (***N*-(4-methoxyphenyl)-*N*-methylacetamide**)** from pMBF (Scheme SI-2) at the **O1** XB accepting site.

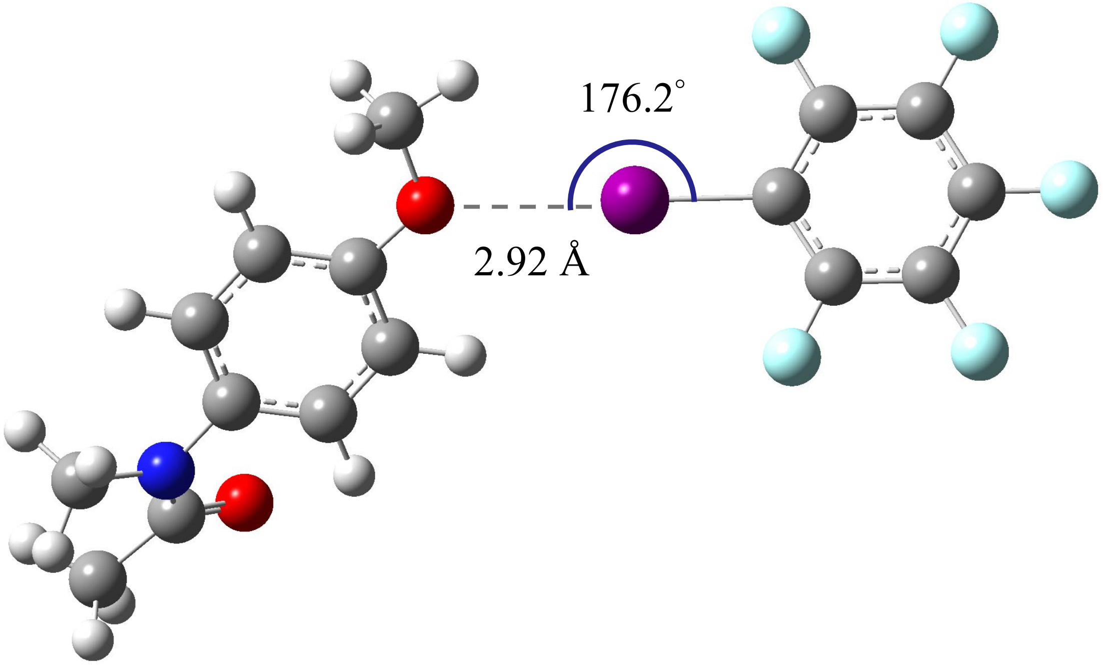

Figure SI-24. DFT-generated, geometry-optimized structure adducts of IPFB interacting with **Fragment 10 (***N*-(4-methoxyphenyl)-*N*-methylacetamide**)** from pMBF (Scheme SI-2) at the **O2** XB accepting site.

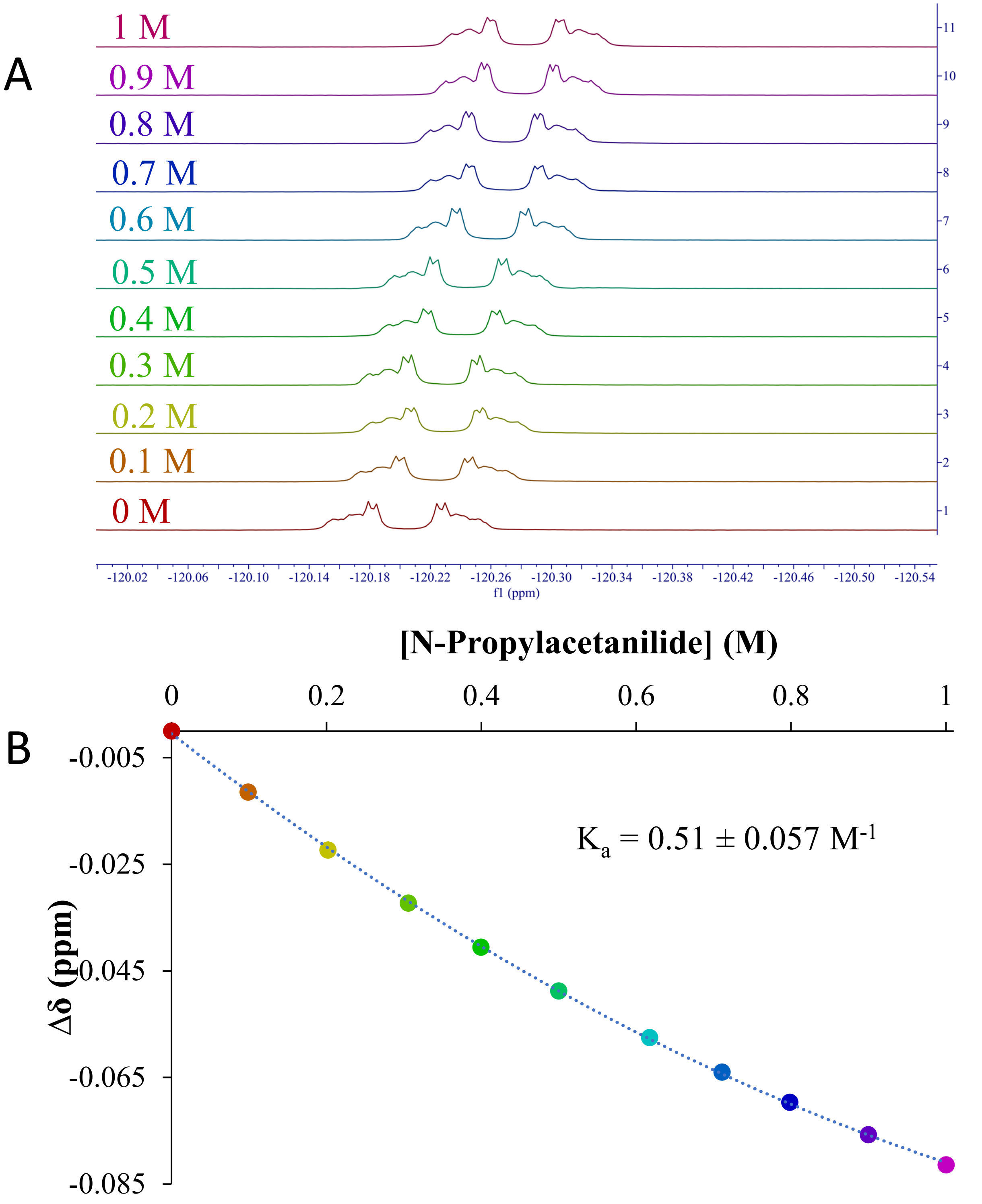

Figure SI-25. (A) ^{19}F NMR spectrum of the ortho-fluorine resonance on IPFB (XB donor) when subjected to increasing concentrations of *N*-phenyl-*N*-propylacetamide, or Fragment 3 from Scheme 2 (XB acceptor); **(B)** corresponding binding isotherm that, when analyzed with non-linear regression, yields and association constant (K_a) value reflective of the XB interaction strength (Solvent: toluene).

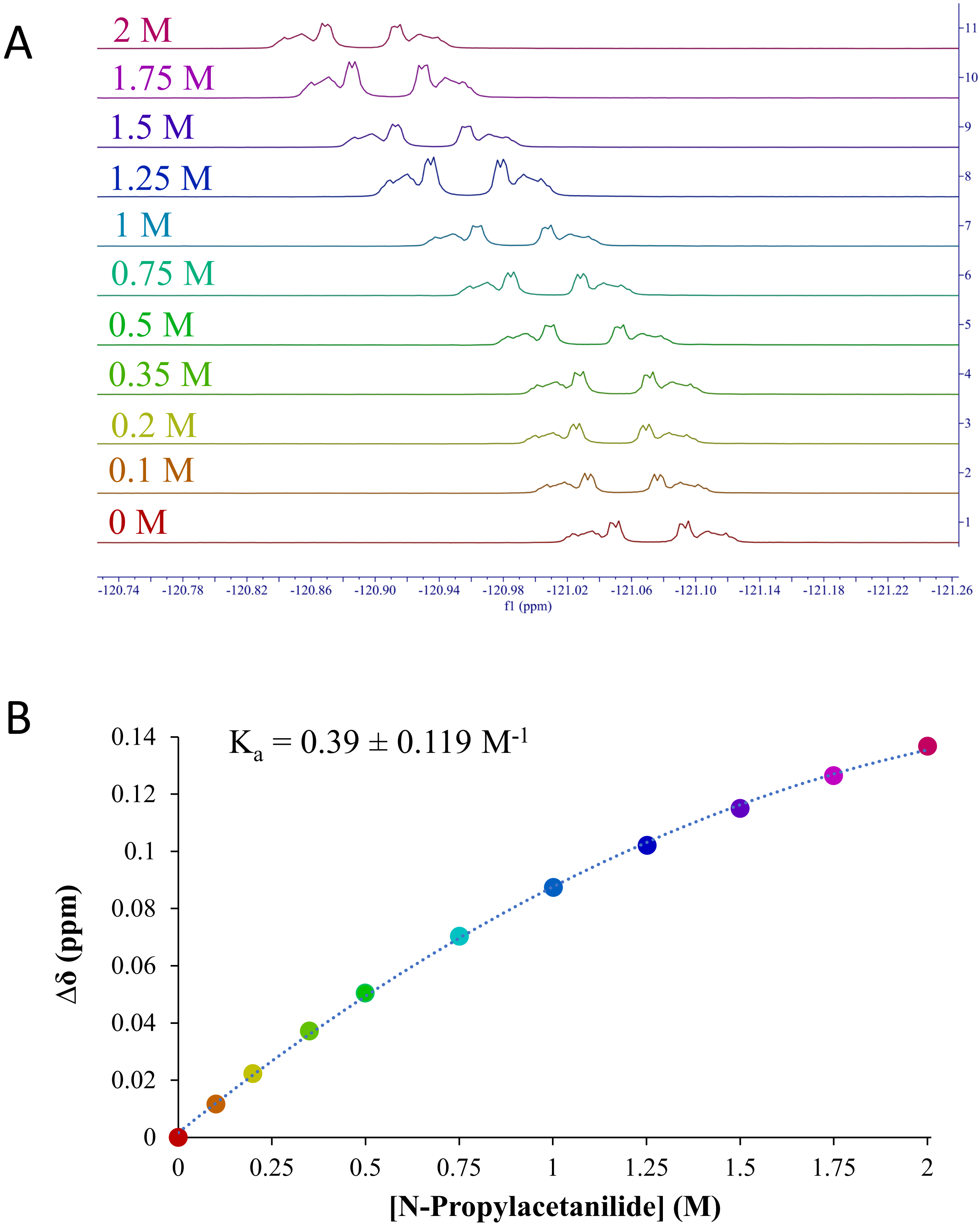

Figure SI-26. (A) ^{19}F NMR spectrum of the ortho-fluorine resonance on IPFB (XB donor) when subjected to increasing concentrations of *N*-phenyl-*N*-propylacetamide, or Fragment 3 from Scheme 2 (XB acceptor); **(B)** corresponding binding isotherm that, when analyzed with non-linear regression, yields and association constant (K_a) value reflective of the XB interaction strength (Solvent: THF).

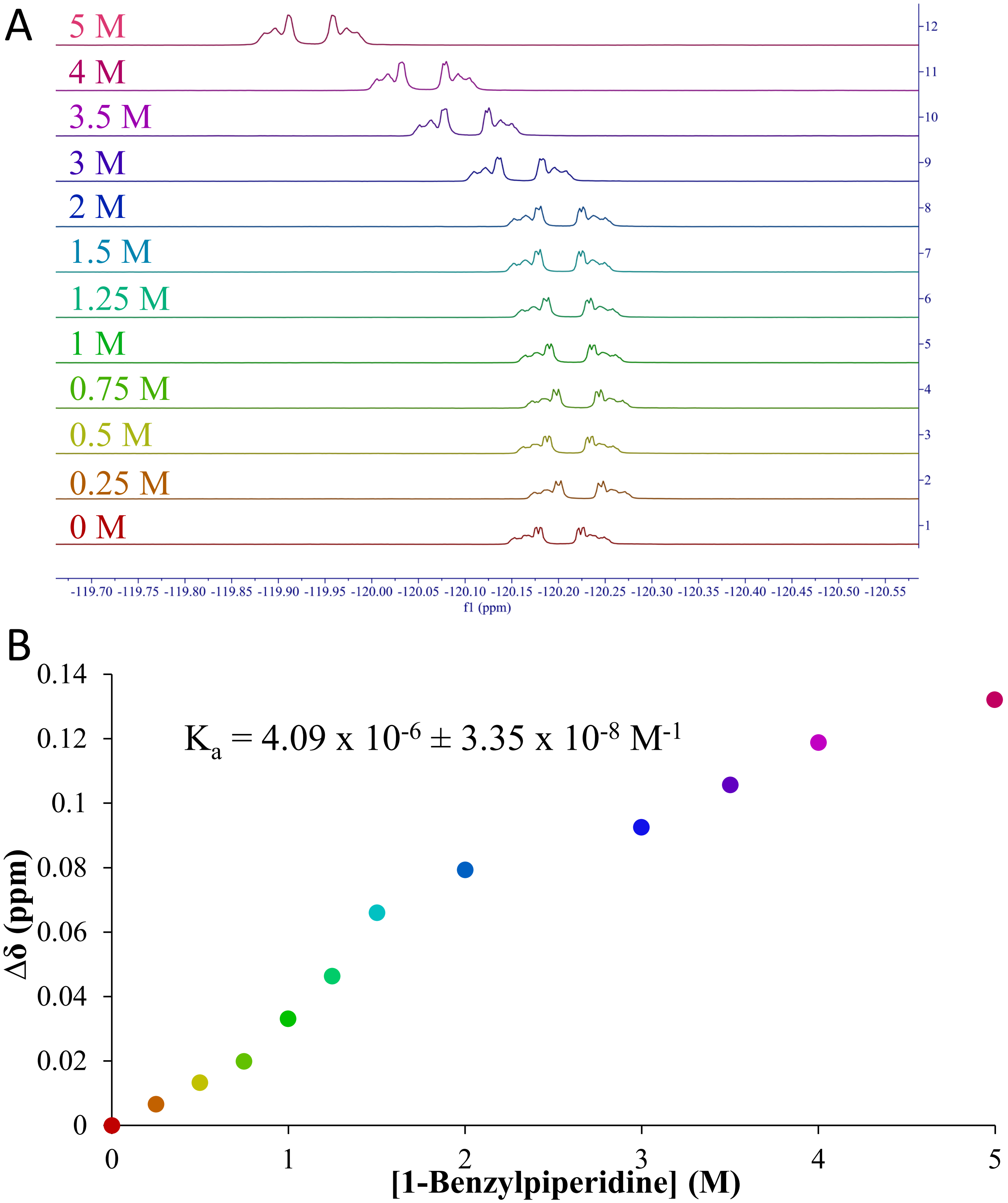

Figure SI-27. (A) ^{19}F NMR spectrum of the ortho-fluorine resonance on IPFB (XB donor) when subjected to increasing concentrations of 1-benzylpiperidine or Fragment 5A from Scheme 2 (XB acceptor); **(B)** corresponding binding isotherm that, when analyzed with non-linear regression, yields and association constant (K_a) value reflective of the XB interaction strength (Solvent: toluene).

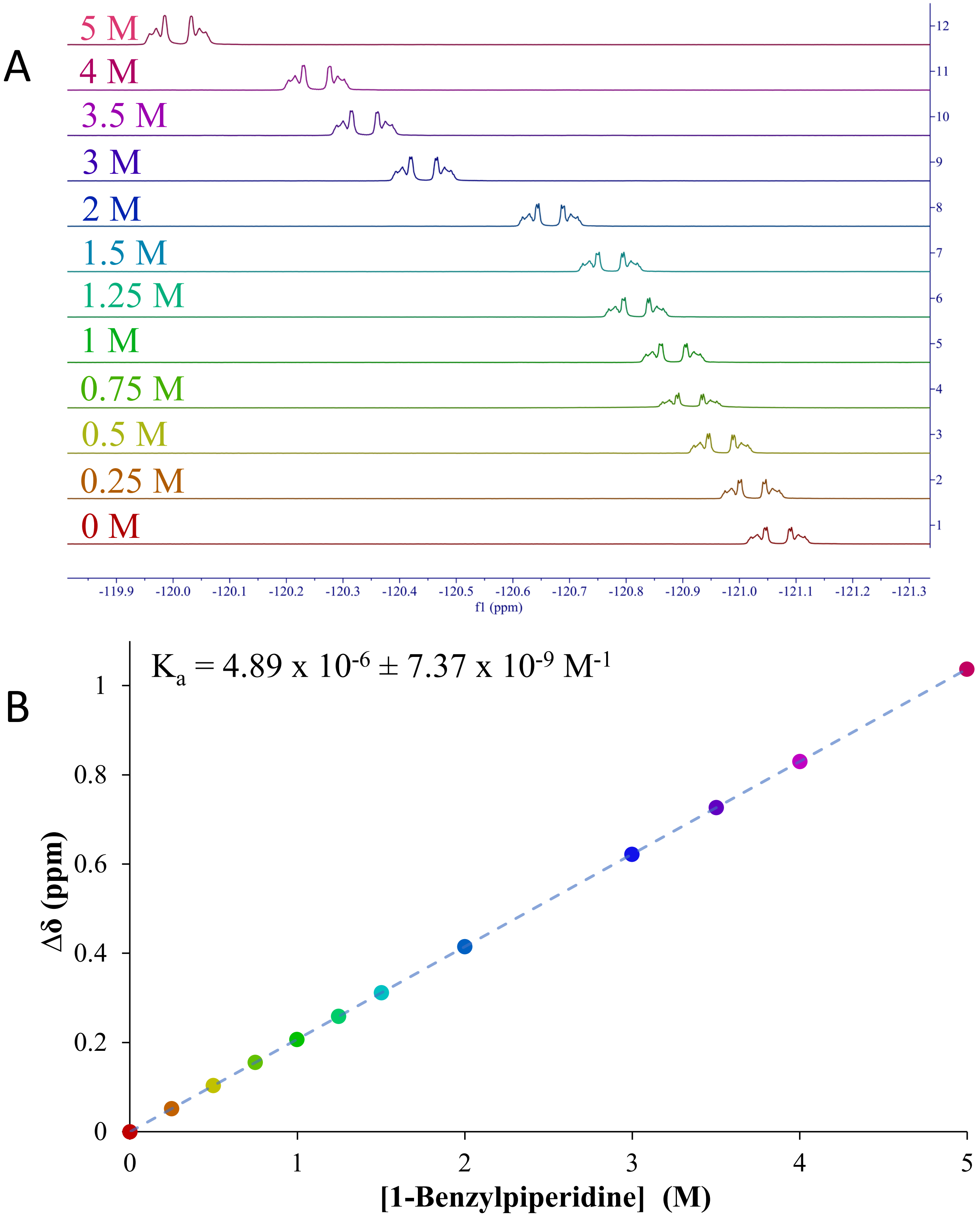

Figure SI-28. (A) ^{19}F NMR spectrum of the ortho-fluorine resonance on IPFB (XB donor) when subjected to increasing concentrations of 1-benzylpiperidine or Fragment 5A from Scheme 2 (XB acceptor); **(B)** corresponding binding isotherm that, when analyzed with non-linear regression, yields and association constant (K_a) value reflective of the XB interaction strength (Solvent: THF).

A

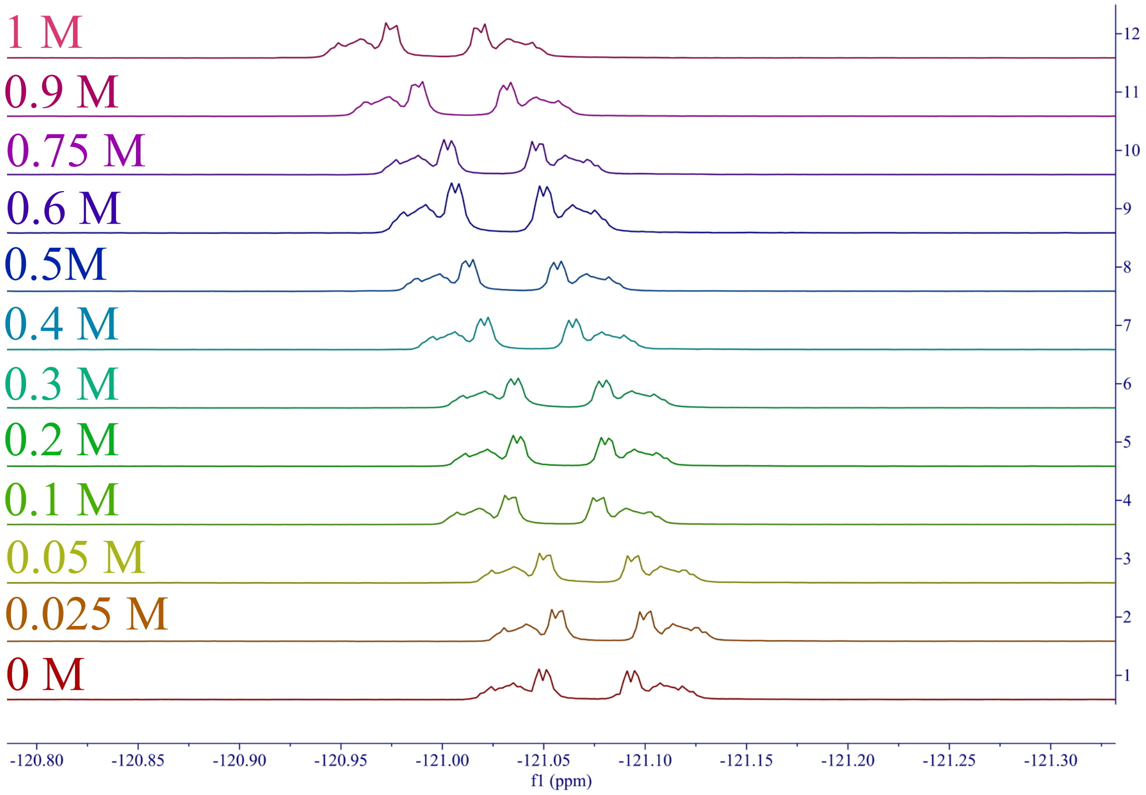

B

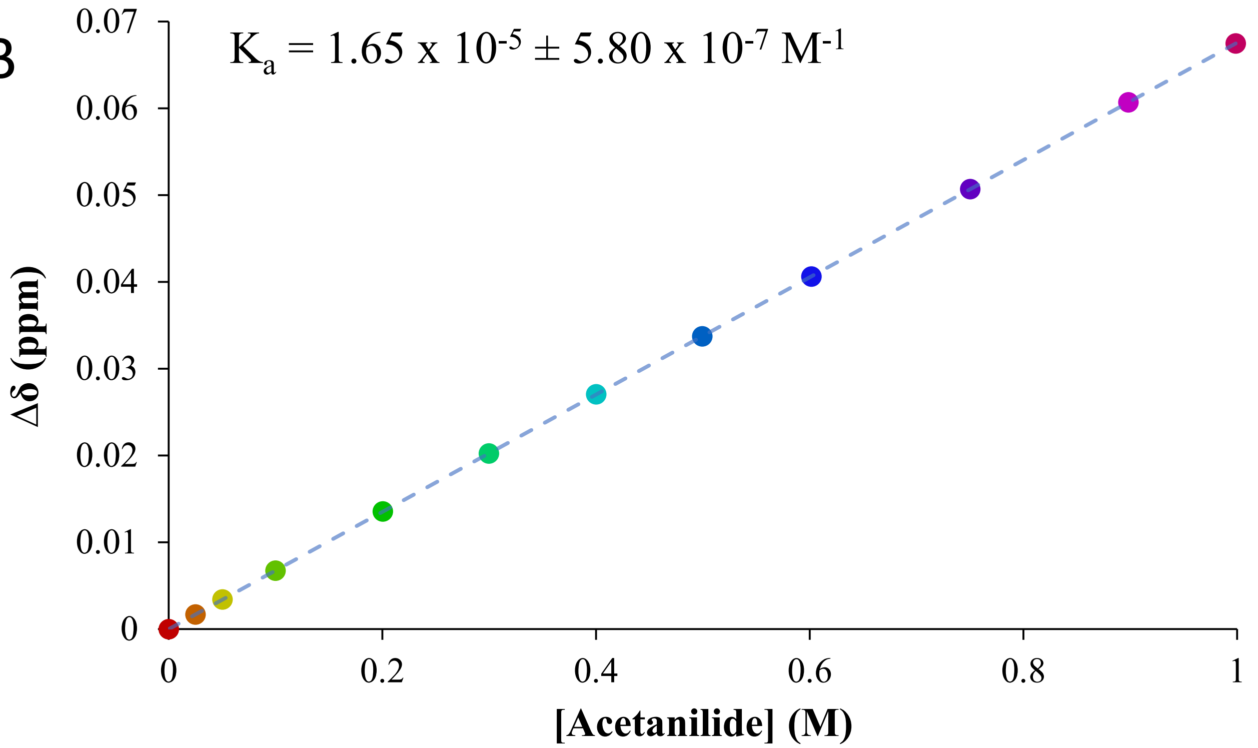

Figure SI-29. (A) ^{19}F NMR spectrum of the ortho-fluorine resonance on IPFB (XB donor) when subjected to increasing concentrations of *N*-phenylacetamide or Fragment 6 from Scheme 2 (XB acceptor); **(B)** corresponding binding isotherm that, when analyzed with non-linear regression, yields and association constant (K_a) value reflective of the XB interaction strength (Solvent: THF).

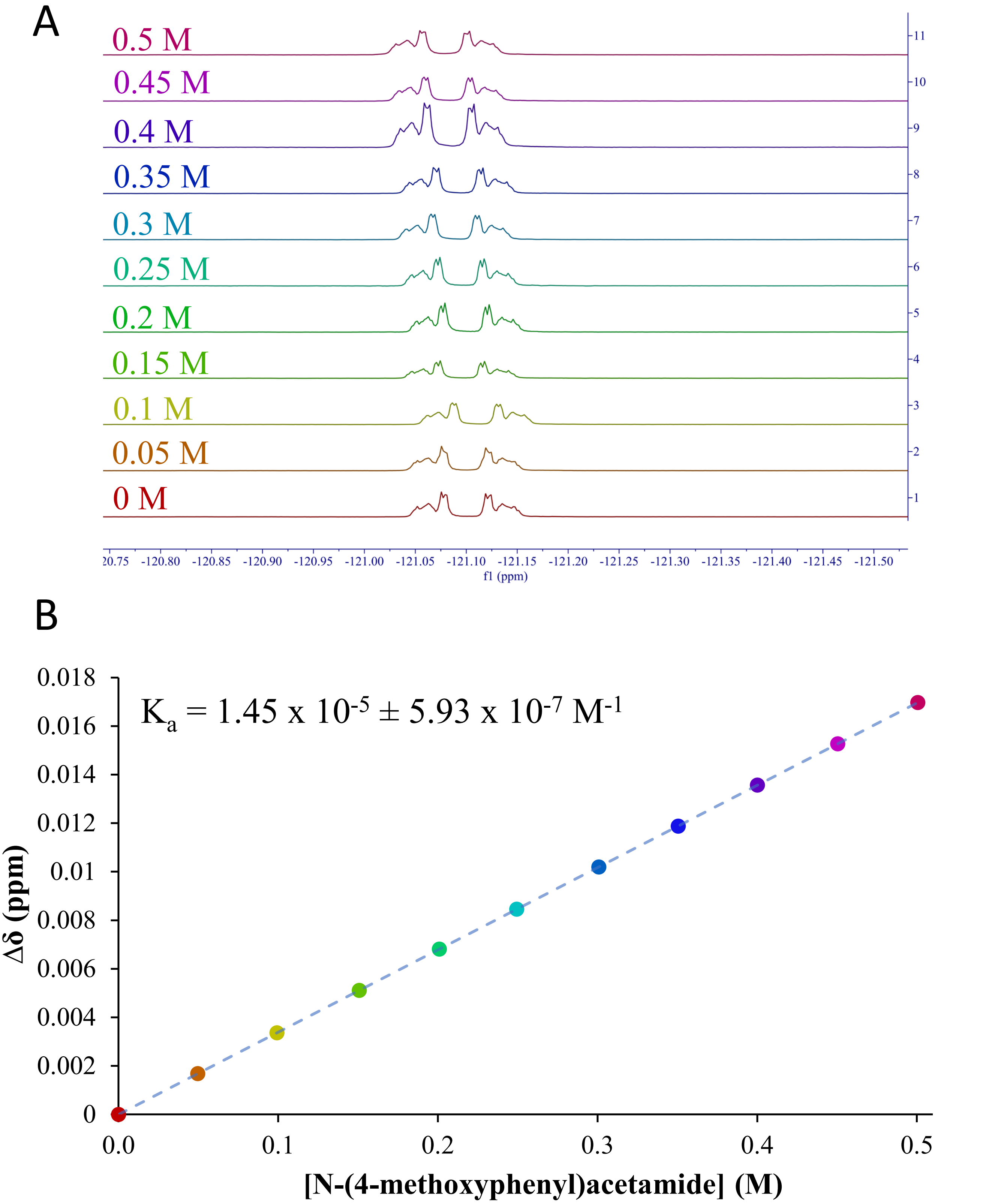

Figure SI-30. (A) ^{19}F NMR spectrum of the ortho-fluorine resonance on IPFB (XB donor) when subjected to increasing concentrations of *N*-(4-methoxyphenyl)-*N*-methylacetamide or Fragment 10 from Scheme SI-2 (XB acceptor); **(B)** corresponding binding isotherm that, when analyzed with non-linear regression, yields and association constant (K_a) value reflective of the XB interaction strength (Solvent: THF).

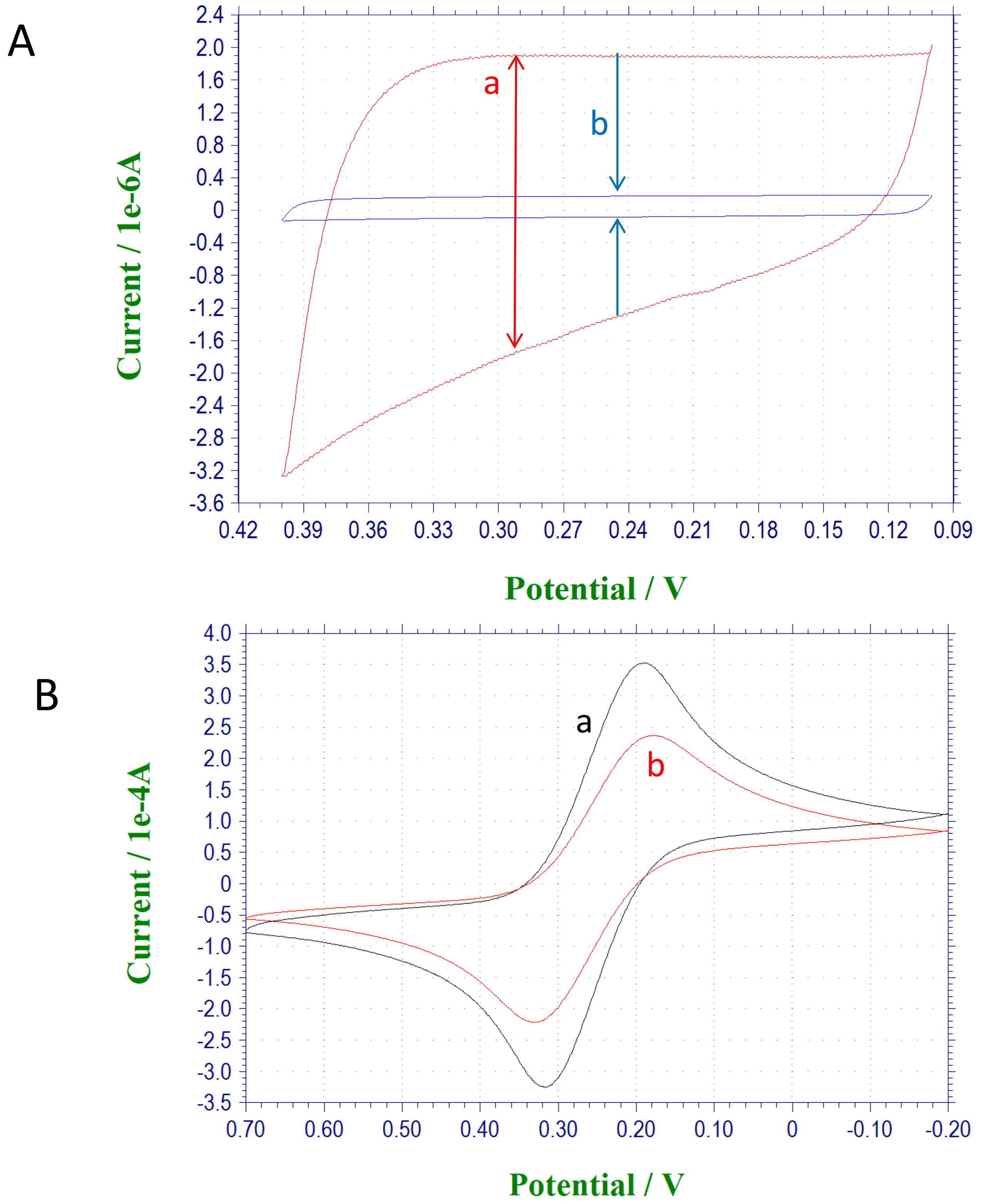

Figure SI-31. (A) Representative CV examples of **(A)** C_{dl} in 4.4 mM PBS and **(B)** 5 mM $K_3Fe(CN)_6$ in 0.5 M KCl at **(a)** bare/clean gold electrode and **(b)** SAM-modifed gold electrodes (i.e., hexanethiolate (C6) (top panel) and pentanethiolate (C5) (bottom panel) SAM-modified electrodes). Note: Scan rate = 100 mV/sec.

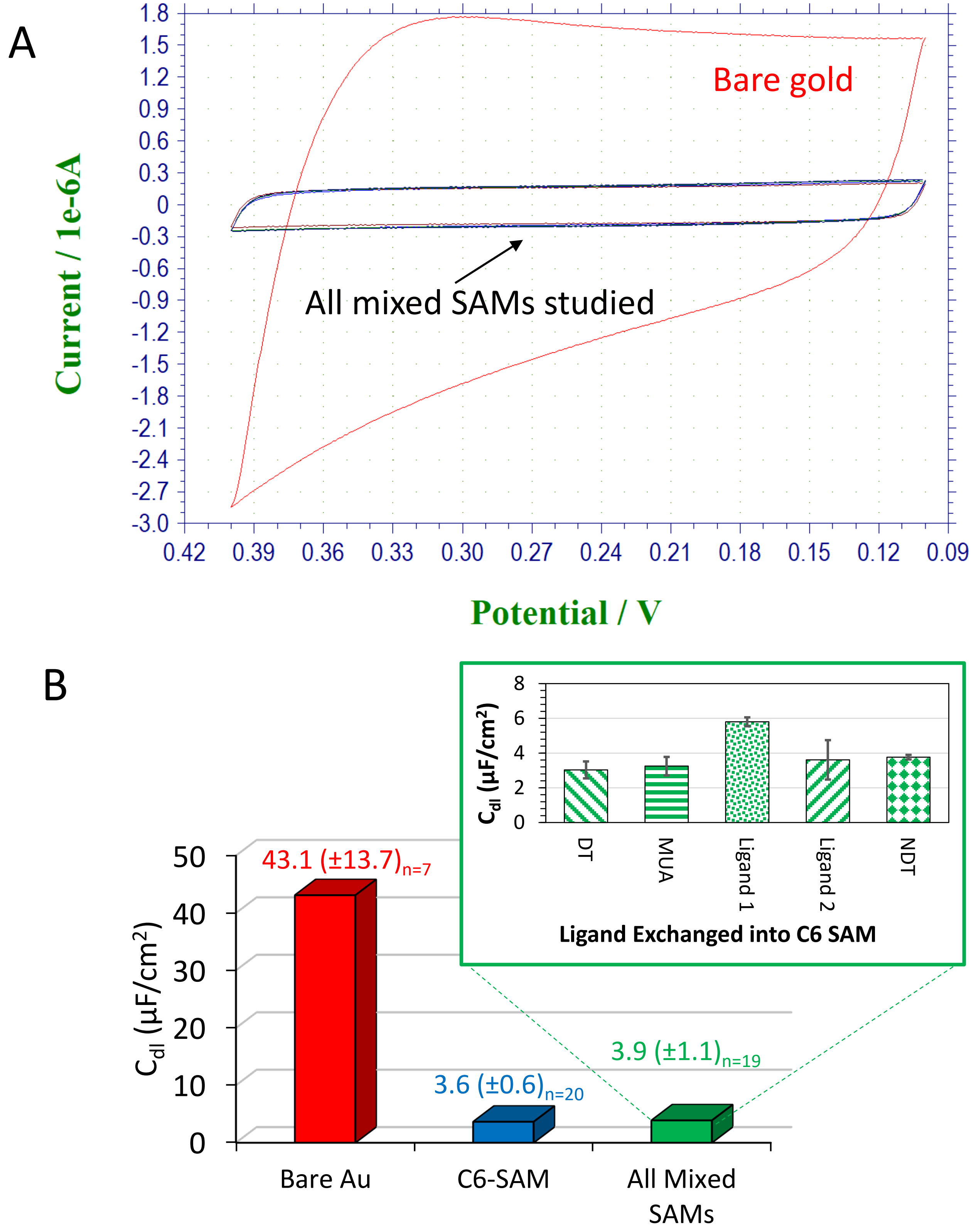

Figure SI-32. (A) Representative CV examples of C_{dl} in 4.4 mM PBS of bare gold versus overlay voltammograms of all the mixed SAMs in the study and; **(B)** C_{dl} results summarized for all films including individual mixed SAMs formed at C6 SAMs with ligands via place-exchange reactions. Note: Scan rate = 100 mV/sec.

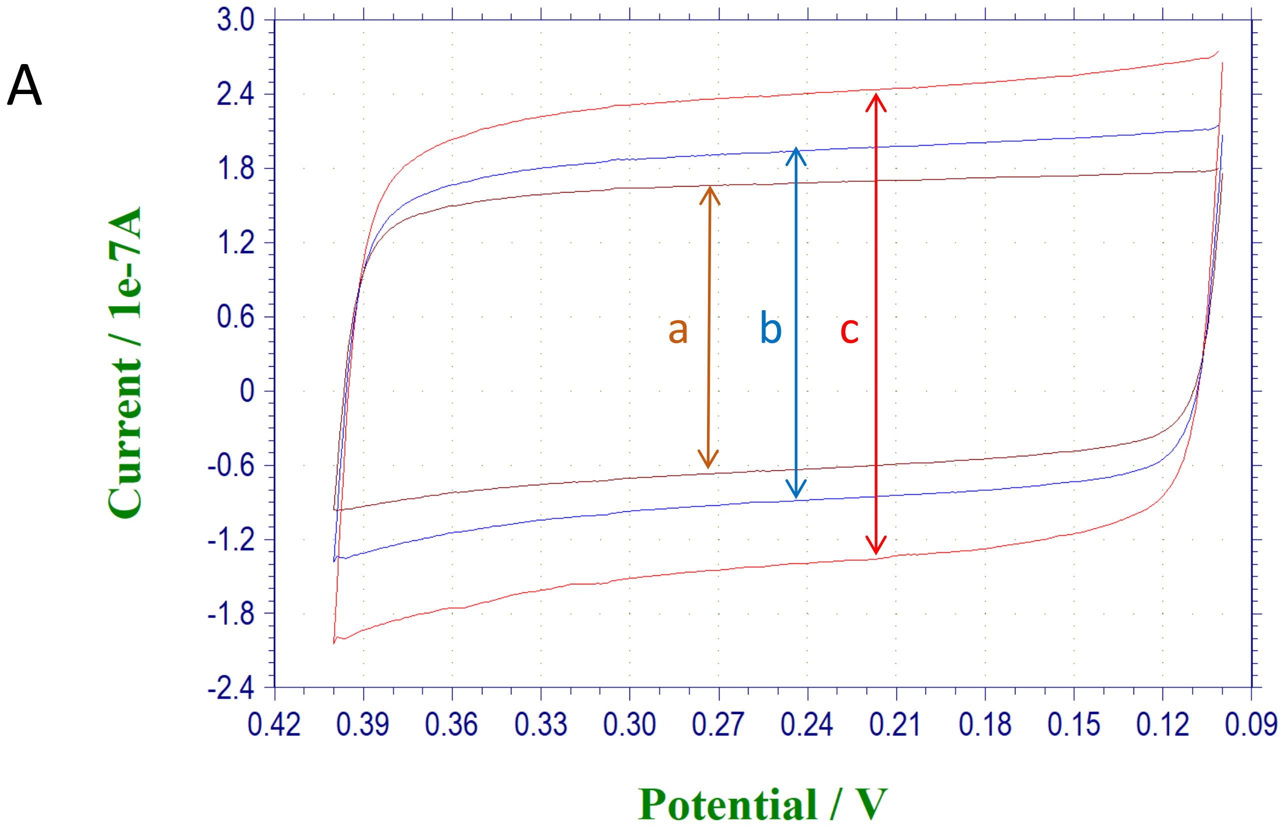

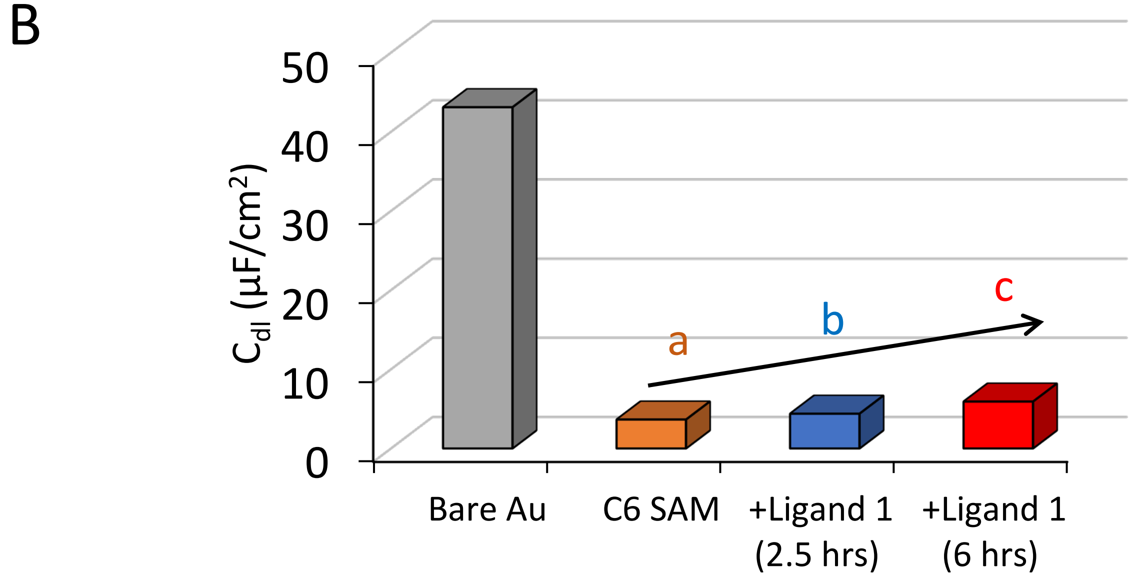

Figure SI-33. (A) Representative CV examples of C_{dl} in 4.4 mM PBS (100 mV/sec) of C6 SAMs **(a)** prior to and after (b) 2.5 hrs and (c) 6 hrs of exposure to a 0.5 mg/mL hexadecafluoro-8-iodooctane-1-thiol (Ligand 1) solution (ethanol); **(B)** C_{dl} results summarized for C6 SAMs as a function of exposure time (i.e., exchange reaction time) to Ligand 1.

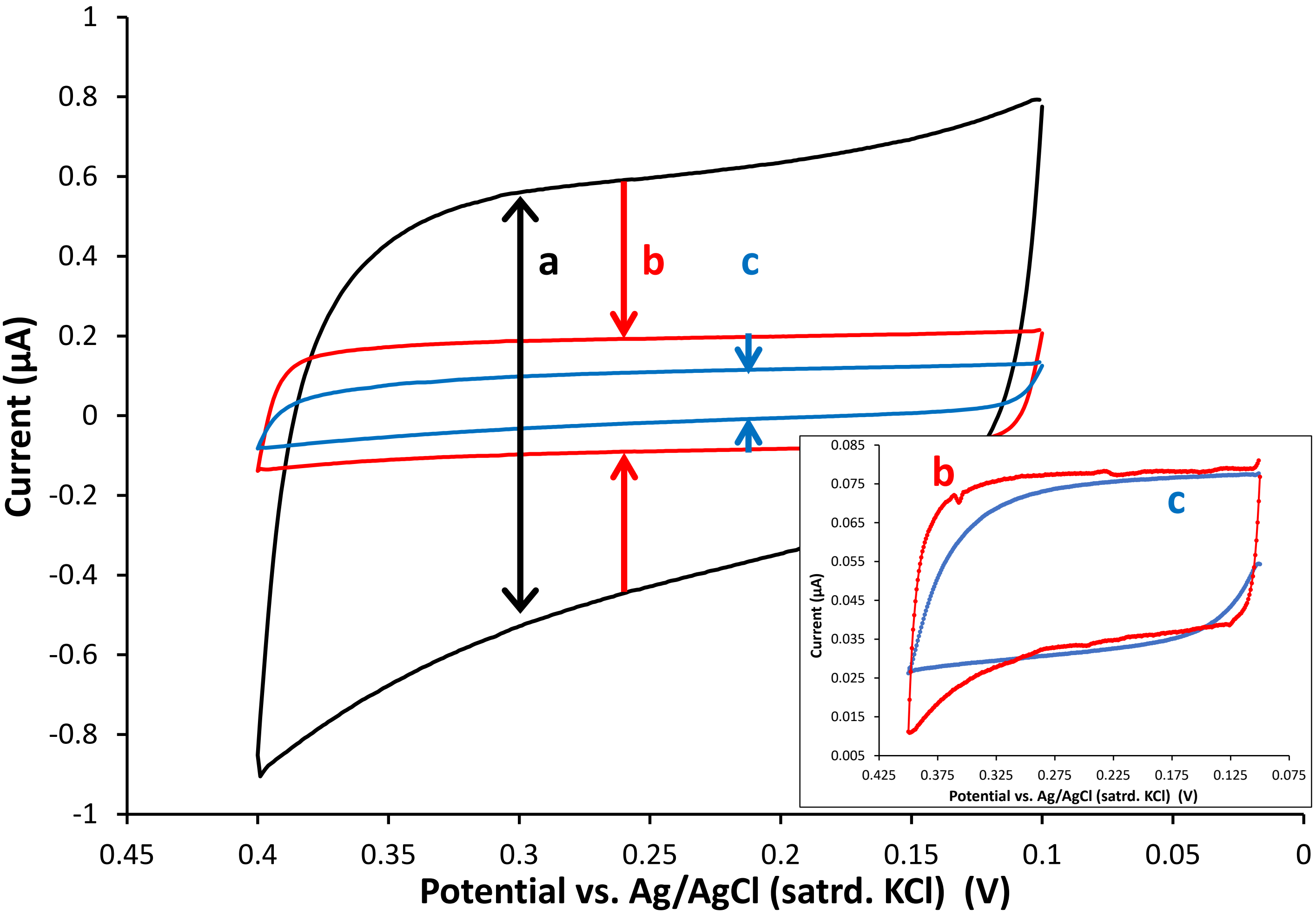

Figure SI-34. Representative CV of C_{dl} (4.4 mM PBS; 100 mV/sec) at **(a)** bare/clean gold electrode, **(b)** a C6/Ligand 1 mixed SAM modification, and **(c)** the same C6/Ligand 1 mixed SAM after exposure to a solution of 5 mM Frag 5A (1-BP) solution (cyclohexane) and; Control film CV of C6/DT mixed SAM exposed to the same Frag 5A solution (**inset**).

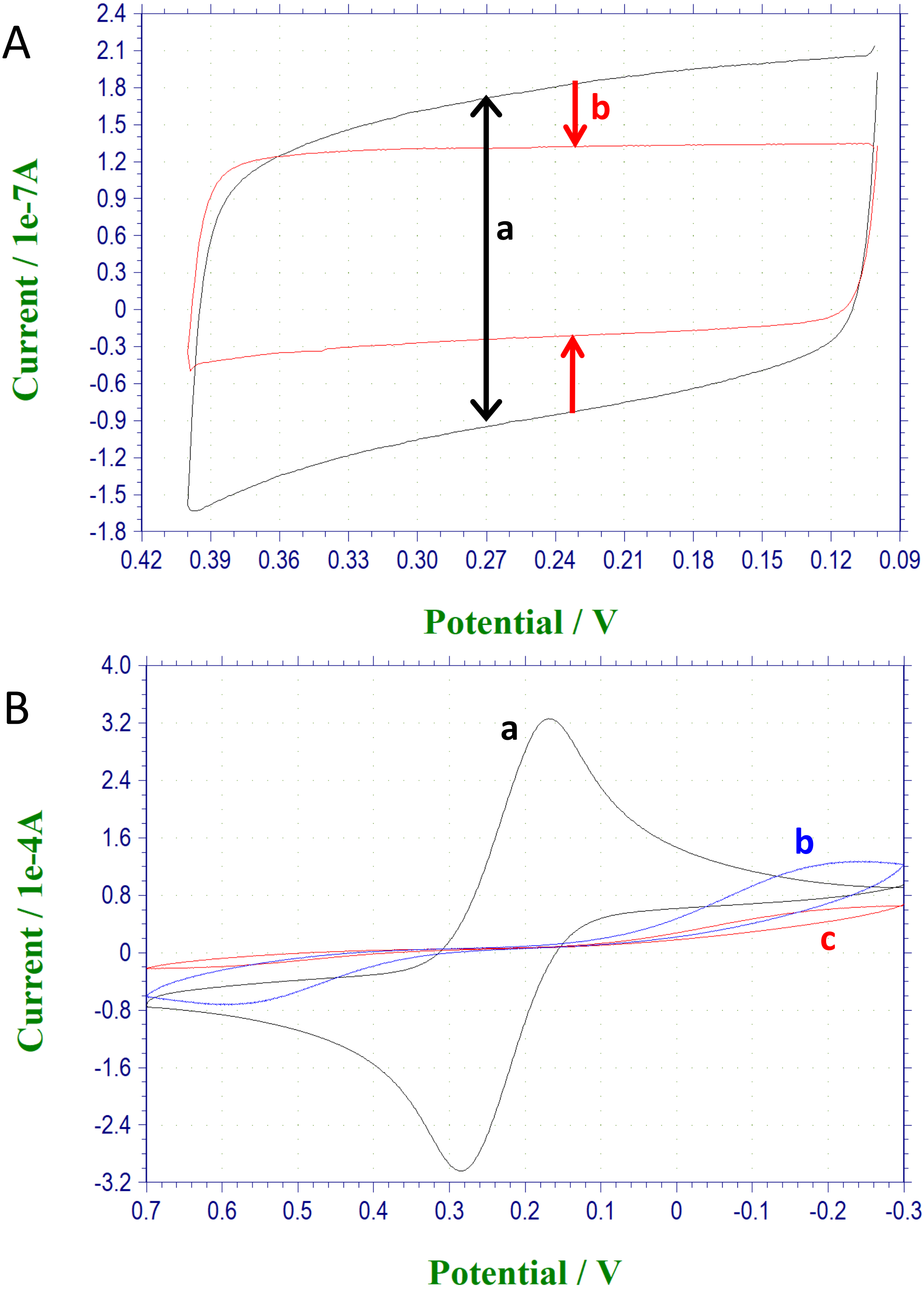

Figure SI-35. Representative CV of **(A)** C_{dl} (4.4 mM PBS) and **(B)** 5 mM $K_3Fe(CN)_6$ in 0.5 M KCl at **(a)** bare/clean gold electrode, **(b)** a C6/**Ligand 2** mixed SAM modification, and **(c)** the same C6/Ligand 2 mixed SAM after exposure to 5 mM Frag 5A (1-BP) solution (cyclohexane); Scan rates = 100 mV/sec

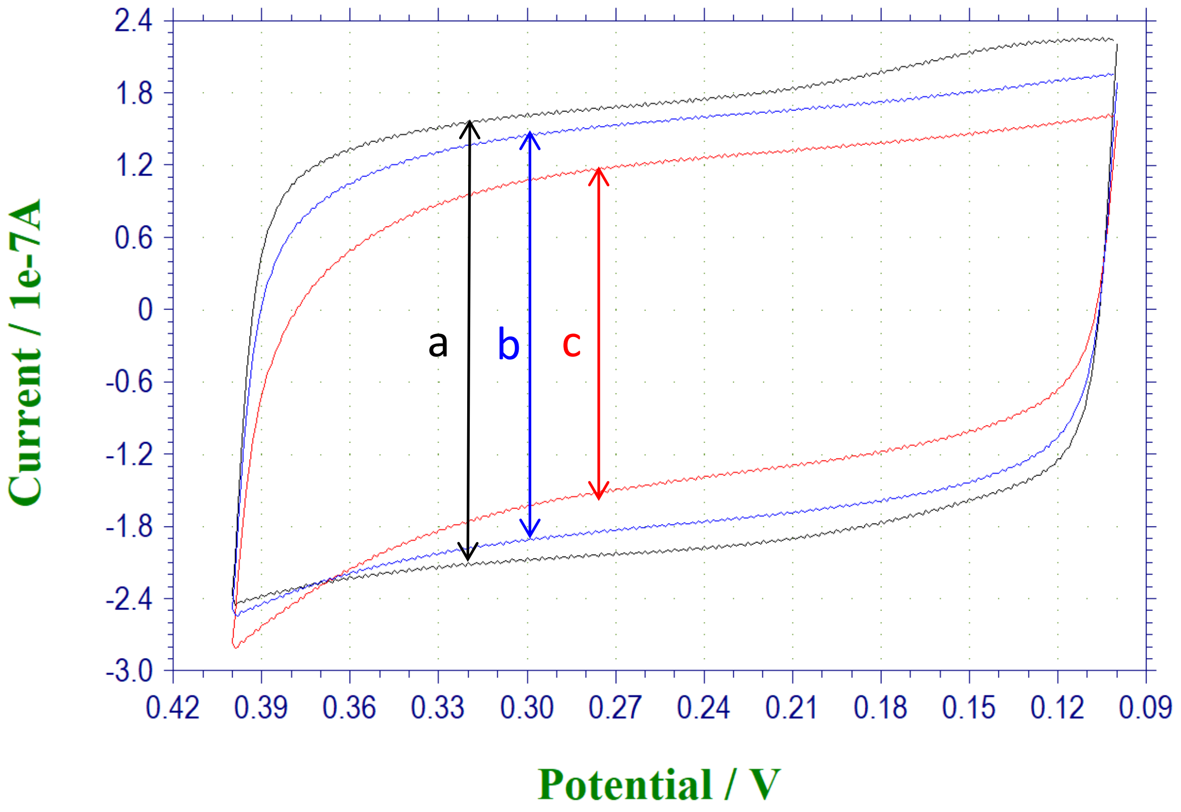

Figure SI-36. Representative CV of C_{dl} (4.4 mM PBS; 100 mV/sec) of a C6/Ligand 1 mixed SAM after **(a)** 0 **(b)** 2.5 and **(c)** 6 hrs of exposure to 5 mM Frag 5A (1-BP) in cyclohexane. Results suggest that longer exposure times results in more significant XB interactions at the interface.

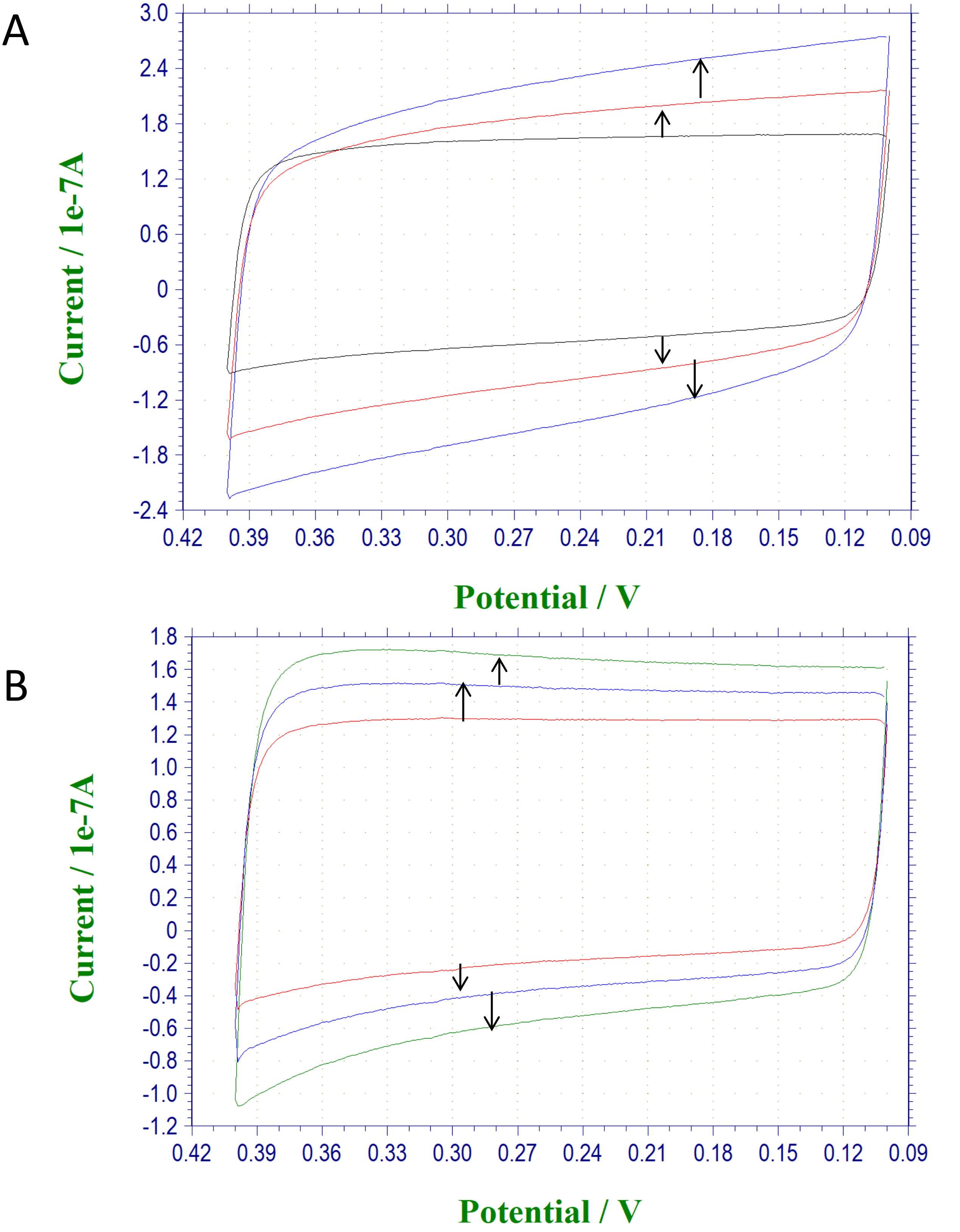

Figure SI-37. Representative CV examples of C_{dl} (4.4 mM PBS; 100 mV/sec) before and after cycles of exposing a C6 SAM modified electrode to dithiol linker molecules and (A) *f*-MPCs and (B) *unf*-MPCs (1 mg/mL in THF and toluene, respectively). Small, sequential increases in C_{dl} for the films indicates that MPCs are attaching the SAM-interface.

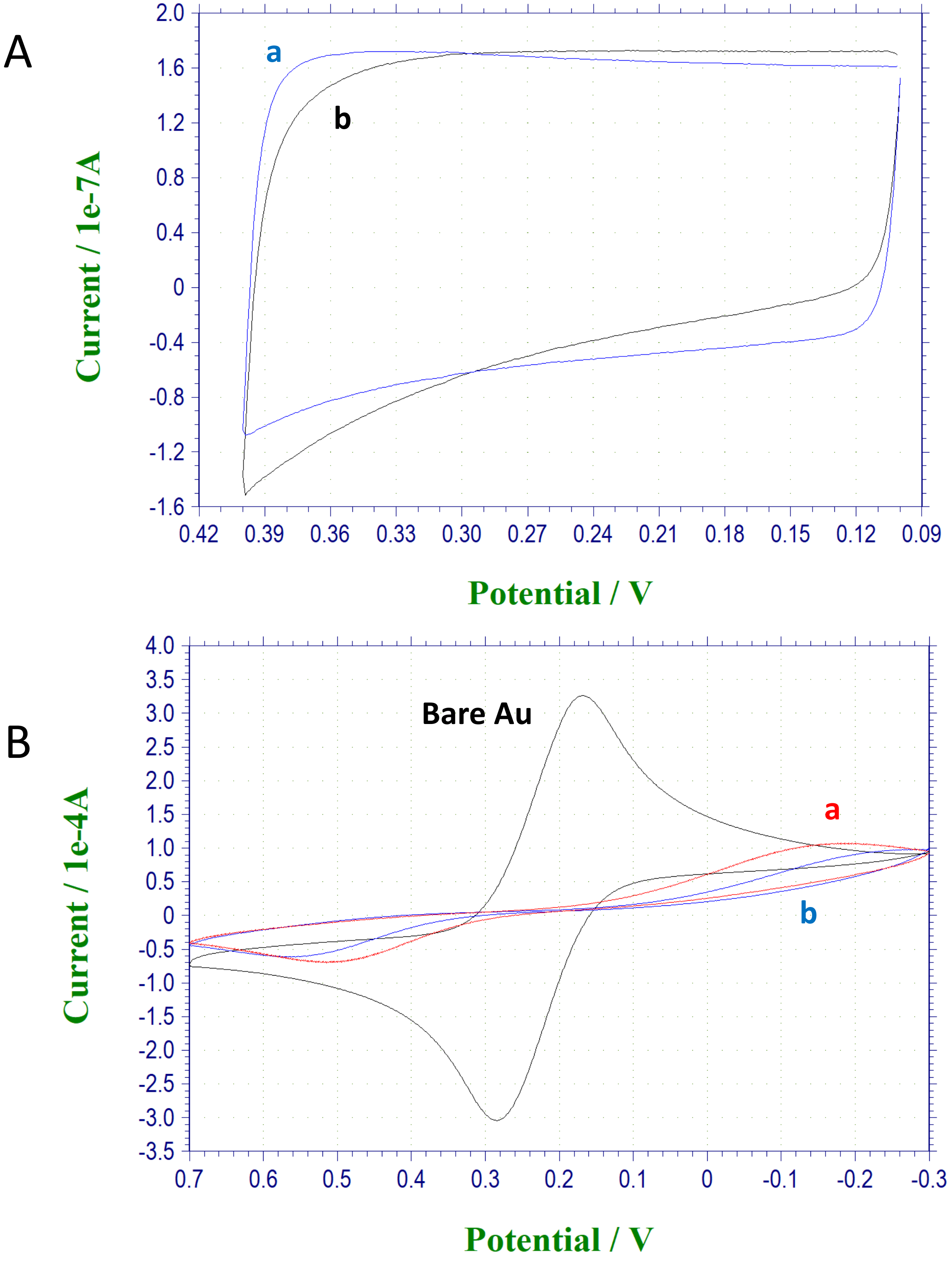

Figure SI-38. (A) Representative CV showing **(A)** C_{dl} and **(B)** 5 mM $K_3Fe(CN)_6$ in 0.5 M KCl at *unf*-MPC film assemblies **(a)** before and **(b)** after exposure to a 5 mM solution of Frag 5A (1-BP) (cyclohexane). Note: Scan rate = 100 mV/sec

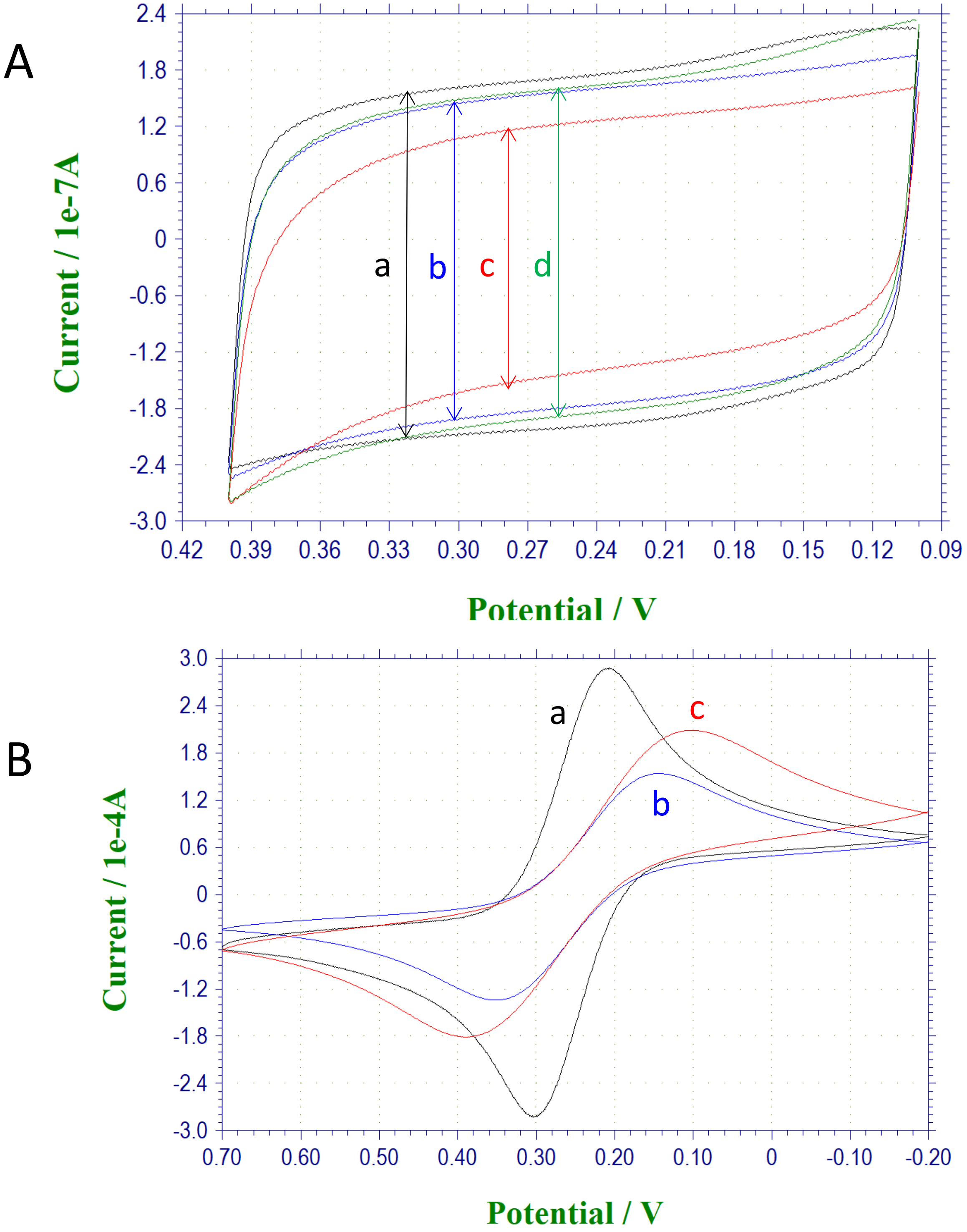

Figure SI-39. (A) Representative CV of C_{dl} (4.4 mM PBS; 100 mV/sec) of a C6/Ligand 1 mixed SAM after **(a)** 0 **(b)** 2.5 and **(c)** 6 hrs of exposure to 5 mM Frag 5A (1-BP) in cyclohexane followed by a thorough rinsing with a mixture of EtOH/MeOH/H_2O which returns the Cdl to the C6/Ligand 1 SAM level prior to Frag 5A exposure; **(B)** CV of 5 mM $K_3Fe(CN)_6$ in 0.5 M KCl at **(a)** bare/clean gold electrode, **(b)** C6/Ligand 2 mixed SAM after exposure to Frag 5A and **(c)** subsequently rinsed with a mixture of EtOH/MeOH/H_2O.

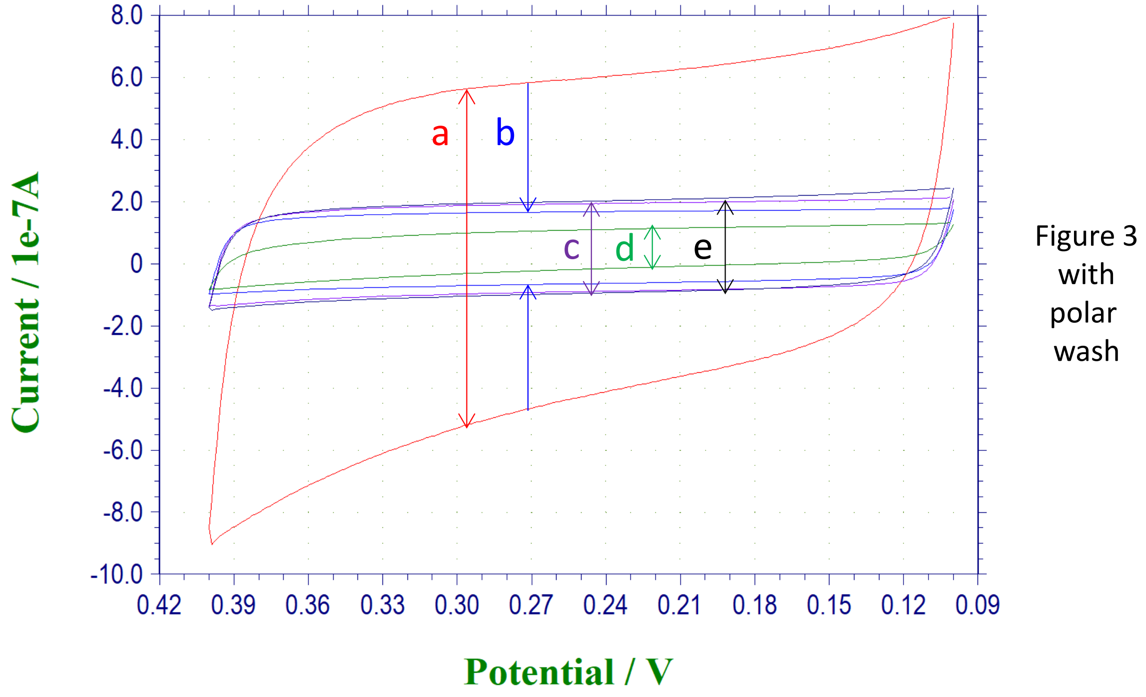

Figure SI-40. Representative CV of C_{dl} (4.4 mM PBS) of **(a)** bare gold; **(b)** C6 SAM modification; **(c)** C6 SAM exchanged with Ligand 1; **(d)** C6/Ligand 1 mixed SAM after exposure to 5 mM Frag 5A (1-BP) solution (cyclohexane) followed by **(e)** the same film rinsed with mixture of MeOH/EtOH/H_2O. Note: Scan rates = 100 mV/sec.

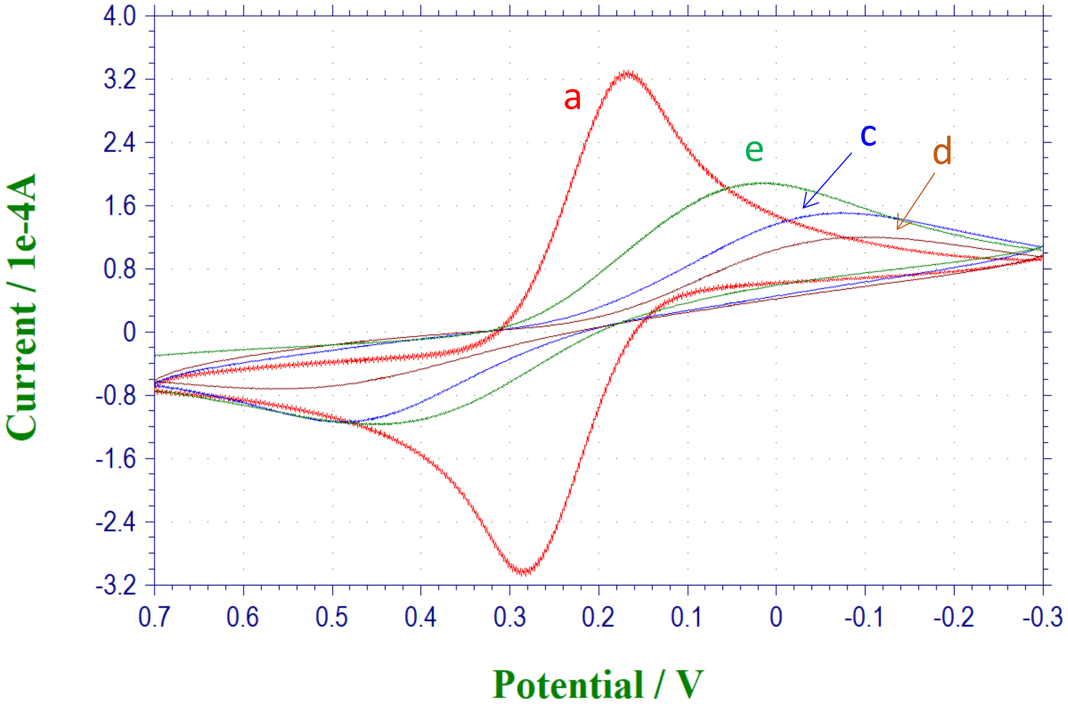

Figure SI-41. Representative CV of 5 mM $K_3Fe(CN)_6$ in 0.5 M KCl at **(a)** bare/clean gold electrode, **(b)** C6 SAM (not shown); **(c)** C6/Ligand 1 mixed SAM before and **(d)** after exposure to Frag 5A and **(e)** subsequently rinsed with a mixture of EtOH/MeOH/H_2O. Note: Scan rates = 100 mV/sec.

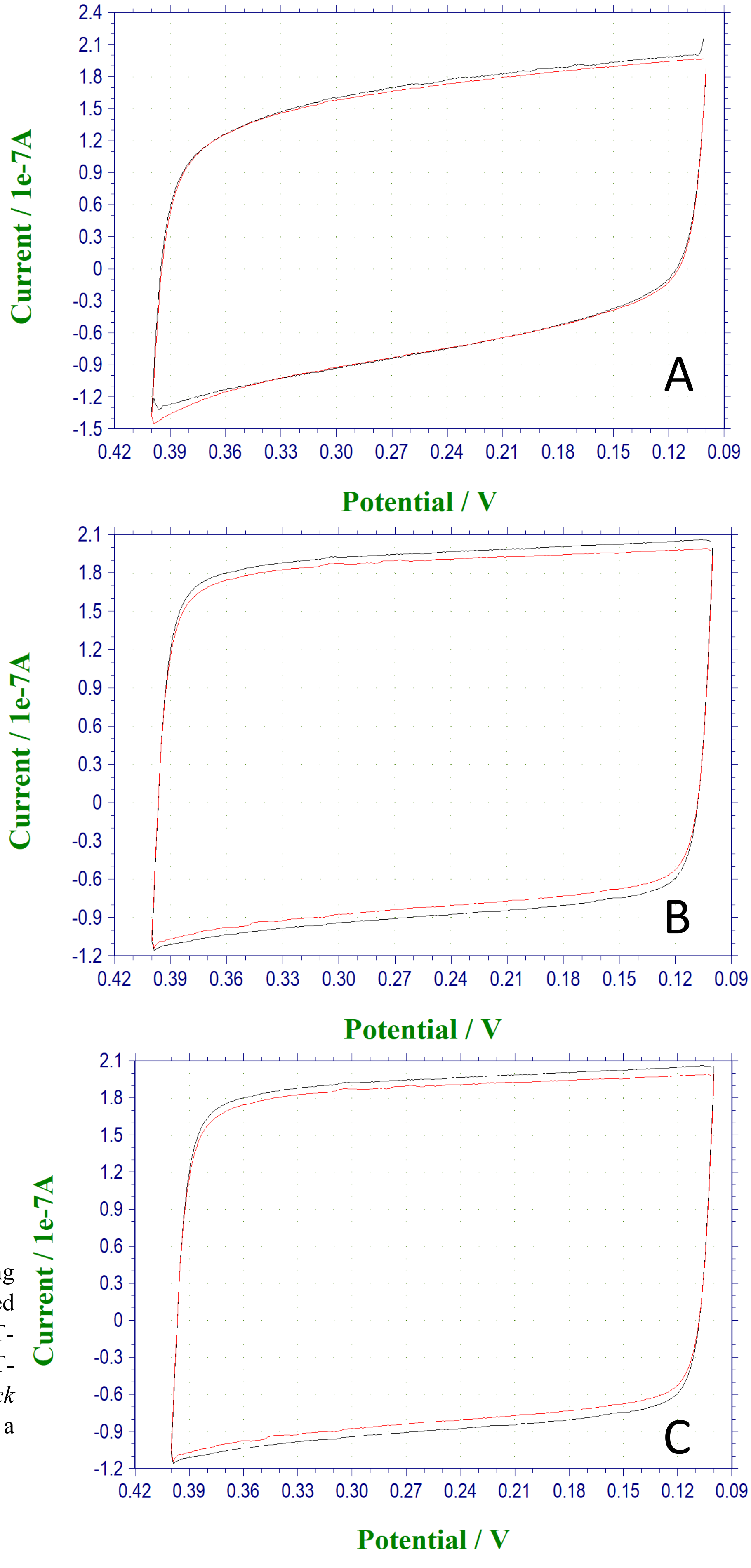

Figure SI-42. Representative CV showing C_{dl} (4.4 mM PBS; 100 mV/sec) of mixed SAMs of **(A)** C6/Ligand 2; **(B)** UDDT-linked *f*-MPC films; and **(C)** UDDT-linked *unf*-MPC films before (*blue/black traces*) and after (*red traces*) exposure to a 5 mM solution of Frag 2 (cyclohexane).
